# *Elymus chaobotryus* (Poaceae), a new species from the north-eastern Qinghai-Tibetan Plateau, China

**DOI:** 10.3897/phytokeys.279.181697

**Published:** 2026-09-24

**Authors:** Yong-Chao Zhang, Sa Xiao, Xin-Yun Ma, Ji-Gui Chen, Yan Qin, Yong Liu, Zhi-Ying Guo, Yu-Qing Zhou, Dian-Zhi Zhao, Qing-Sheng Shan, Hai-Zhang Lu, Xiao-Xing Wei

**Affiliations:** 1 Key Laboratory of the Alpine Grassland Ecology in the Three Rivers Region (Qinghai University) Ministry of Education, Qinghai University (Qinghai Academy of Animal Science and Veterinary), Qinghai Province, Xining, China Key Laboratory of the Alpine Grassland Ecology in the Three Rivers Region (Qinghai University) Ministry of Education, Qinghai University (Qinghai Academy of Animal Science and Veterinary) Xining China; 2 Qinghai Yuancaosheng Agriculture and Animal Husbandry Technology Co., Ltd., Xining, Qinghai Province, China Qinghai Yuancaosheng Agriculture and Animal Husbandry Technology Co., Ltd. Xining China; 3 Museum of Qinghai-Tibet Plateau Biology, Northwest Institute of Plateau Biology, Chinese Academy of Sciences, Xining, China Museum of Qinghai-Tibet Plateau Biology, Northwest Institute of Plateau Biology, Chinese Academy of Sciences Xining China; 4 Haibei Tibetan Autonomous Prefecture Grassland Station, Haibei Tibetan Autonomous Prefecture, Qinghai Province, Xining, China Haibei Tibetan Autonomous Prefecture Grassland Station, Haibei Tibetan Autonomous Prefecture Xining China; 5 Grassland Station, Menyuan Hui Autonomous County, Haibei Tibetan Autonomous Prefecture, Qinghai Province, Xining, China Grassland Station, Menyuan Hui Autonomous County, Haibei Tibetan Autonomous Prefecture Xining China; 6 Hai Bei Demonstration Zone of Plateau Modern Ecological Animal Husbandry Science and Technology, Qinghai Province, Xining, China Hai Bei Demonstration Zone of Plateau Modern Ecological Animal Husbandry Science and Technology Xining China; 7 Natural Resources, Forestry and Grassland Bureau of Gangcha County, Haibei Tibetan Autonomous Prefecture, Qinghai Province, Xining, China Natural Resources, Forestry and Grassland Bureau of Gangcha County, Haibei Tibetan Autonomous Prefecture Xining China; 8 Xining Botanical Garden, Qinghai Province, Xining, China Xining Botanical Garden Xining China

**Keywords:** Morphology, phylogeny, plastome, Poaceae, Triticeae

## Abstract

*Elymus chaobotryus* Y.C. Zhang, sp. nov. (Poaceae, Triticeae) from the north–eastern Qinghai-Tibetan Plateau (Tongren City, southeastern Qinghai, China) is described and illustrated based on detailed morphological comparisons and complete plastome data. The new species is readily distinguished from morphologically similar taxa by its pendulous compound spike 16–19 cm long, bearing 10–15 short lateral branches (2–4 cm) produced from nearly all nodes of the main rachis; the branches become progressively longer toward the base, giving the inflorescence a characteristic grape-like appearance. It further differs in having lanceolate glumes 3–4 mm long with mucros (< 0.9 mm), lanceolate lemmas 5–7 mm long with awns (3–4 mm), and conspicuously narrow leaf blades (2–5 mm wide). Flow-cytometric analysis estimated a genome size of 11.43 Gb, while chromosome and cytogenetic analyses confirmed a hexaploid chromosome complement (2n = 6x = 42) and were consistent with an StHY genomic constitution. In plastome-based phylogenetic analyses, *E. chaobotryus* is placed within the broader *Elymus* complex and is recovered as sister to *E. dahuricus*.

## Introduction

Poaceae is a diverse and economically important flowering plant family, and inflorescence and spikelet morphology have long been important in grass taxonomy (Kellogg et al. 2013; Kellogg 2022). In Triticeae, particularly in *Elymus* s.l., polyploidy, hybridization and reticulate evolution complicate classification, and modern studies increasingly draw on morphological, cytogenetic and molecular phylogenetic data (Liu et al. 2006; Fan et al. 2013; Dong et al. 2015). *Elymus* L. (wheatgrasses or wild ryes) is the largest genus in the wheat tribe Triticeae Dumort. (Poaceae Barnhart), encompassing more than 150 perennial species distributed across temperate regions of Asia, Europe, and the Americas (Liu et al. 2006; Dong et al. 2015). Historically, *Elymus* was primarily delimited using inflorescence morphology, and the number of spikelets per rachis node was commonly used to separate *Elymus* (often with two or more spikelets per node) from *Agropyron* Gaertn. and allied genera with solitary spikelets (including *Sitanion* Raf.) (Nevski 1934). Early taxonomic works varied in their treatment of Asiatic StY-allotetraploids, either placing them in *Elymus* or in allied genera, reflecting the complexity of their morphological diversity (Bentham and Hooker 1883). A paradigm shift occurred in 1984 when Áskell Löve and D.R. Dewey independently proposed classifying Triticeae genera by genomic constitution (Dewey 1984; Löve 1984; Yen et al. 2005). This framework broadened *Elymus* sensu lato to encompass St-genome polyploids and stimulated the recognition of genomically defined genera (e.g., *Pseudoroegneria* (Nevski) Á. Löve for St, *Australopyrum* (Nevski) Á. Löve for W), which were subsequently formalized in North American treatments (Barkworth and Dewey 1985). Within this context, the distinction between *Elymus* and *Roegneria* K. Koch became central: Asiatic StY allotetraploids, typically with a single spikelet per rachis node, were long treated in *Roegneria* (Baum et al. 1991; Yen and Yang 2011), whereas broader circumscriptions retained these taxa in *Elymus* s.l. (Löve 1984; Barkworth and Dewey 1985). Baum et al. (1991) and Yen and Yang (2011) further advocated recognition of *Roegneria* as a separate genus based on genomic evidence, consistent with modern molecular systematics in Triticeae. Throughout these debates, cytogenetic and molecular studies confirmed that *Elymus* species are allopolyploids, each possessing one St haplome (from *Pseudoroegneria*) combined with additional haplomes from *Hordeum* L. (H), *Agropyron* (P), *Australopyrum* (W), and an enigmatic Y haplome (Liu et al. 2006; Fan et al. 2013; Dong et al. 2015). Recent taxonomic and phylogenetic studies have further refined the circumscription and evolutionary framework of *Elymus*. An integrative taxonomic synopsis of *Elymus* in Uzbekistan combined morphological and molecular evidence and resulted in the description of *E. uzbekistanicus* (Alieva et al. 2025). Genome-wide analyses of *Elymus* s.s. (StH) revealed a major differentiation between Eurasian and American lineages and supported geographically differentiated origins within the StH complex (Leo et al. 2025). At the genomic scale, the chromosome-scale genome of *E. nutans* showed that the Y haplome is sister to the lineage comprising the V and Jv haplomes of *Dasypyrum* and *Thinopyrum*, respectively, providing new insight into the reticulate allopolyploid evolution of *Elymus* (Xiong et al. 2025).

Recent syntheses of floristic, phylogenetic and ecological data highlight the Qinghai-Tibetan Plateau (QTP) and the adjacent Himalaya–Hengduan Mountains as a major centre of mountain plant diversity and diversification (Wen et al. 2014; Favre et al. 2015; Liu et al. 2021; Mao et al. 2021). Several species-rich genera with substantial diversification in this region include *Gentiana*, *Pedicularis*, *Primula*, *Rhododendron*, *Saussurea* and *Koenigia*, for which diversification and biogeographic differentiation have been documented in the QTP–Himalaya–Hengduan region (Shrestha et al. 2018; Rana et al. 2023). Recent targeted field work and molecular studies have revealed additional St-genome taxa in the region. *Elymus multiramosus* Y.C. Zhang was recently described from a dry, rocky slope near Delingha in western Qinghai, distinguished by its unique compound-spike inflorescence (Zhang et al. 2024a), and *Roegneria yenchiana* X. Fan & L. N. Sha was reported from the Hengduan Mountains on the southeastern edge of the plateau as a new StY allotetraploid (Sha et al. 2024). These cases suggest that continued exploration and integrative taxonomic work are expected to reveal further unrecognised St-genome taxa in this region. The genomic-constitution framework for Triticeae (Dewey 1984; Löve 1984) is followed here as the operational taxonomic system. For the purposes of this study, *Elymus* is treated in a broad, operational sense (*sensu lato*) for St-containing polyploids. Taxa treated as *Roegneria* in some classifications, notably Asiatic StY allotetraploids, are discussed explicitly, but *Elymus* is retained throughout for consistency with the sampling, nomenclature, and plastome-based analyses.

Grass inflorescences exhibit remarkable diversity, influenced by both genetic and environmental factors (Kellogg et al. 2013; Whipple 2017). The basic unit of the grass inflorescence is the spikelet, and differences in its arrangement and branching patterns lead to a wide variety of inflorescence forms across the Poaceae. Inflorescences can range from simple, unbranched spikes or compound spikes to more complex structures, such as panicles, corymbs, and thyrses (Kellogg et al. 2013; Whipple 2017). In most Triticeae grasses, including typical *Elymus*, the inflorescence is a simple, unbranched spike, with one or a few laterally compressed spikelets per rachis node. The spike is usually terminal, either erect or nodding, and typically bears one or two spikelets per node (Bommert and Whipple 2018). Branching of the spike axis is exceedingly rare in this tribe. In wheat, the ‘Miracle wheat’ exhibits a unique inflorescence structure, producing lateral meristems that sprout mini-spike-like branches from the basal part of the main spike axis. These mini-spikelets can generate additional spikelets in a distichous arrangement, leading to an indeterminate number of spikelets per spike (Poursarebani et al. 2015). Similarly, in rice, mutants have been created to explore the role of primary and secondary branches in shaping inflorescence morphology (Gao et al. 2010). In contrast, *Elymus multiramosus* represents a notable exception to the typical spike architecture seen in *Elymus*. Its spike base produces several lateral branchlets, or extended rachillas, each bearing additional spikelets, resulting in a conspicuously many-branched, compound spike (Zhang et al. 2024a). Previous studies of alpine *Elymus* germplasm from the Qinghai-Tibetan Plateau have further documented diverse branched-inflorescence resources and quantified variation in branch number, branch position, spike structure and seed-yield components (Zhang et al. 2024b; Yu et al. 2024). This rare morphology is highly unusual within *Elymus* and raises intriguing questions about the taxonomic and evolutionary significance of compound spike-like structures in the genus. These findings suggest that both genetic and environmental factors may play a role in shaping the development of such complex inflorescence forms, providing new insights into the evolutionary patterns of spike structure within *Elymus*.

During recent field surveys on the north-eastern QTP, we encountered an unusual *Elymus* population with a conspicuously compound, many-branched spike-like inflorescence in southeastern Qinghai, China. Accordingly, we describe and illustrate this new species and discuss its morphological differentiation and phylogenetic placement.

## Materials and methods

### Herbarium survey

A herbarium survey was conducted to assess whether the compound-spike morphology of *Elymus chaobotryus* falls within the documented morphological variation of *E. dahuricus* s.l. or other morphologically related and regionally occurring *Elymus* taxa. Herbarium records of *E. dahuricus* were screened from Qinghai, Gansu, Xinjiang, Tibet, Inner Mongolia and Sichuan. In total, 660 specimens were examined, including 155 from Qinghai, 123 from Gansu, 67 from Xinjiang, 64 from Tibet, 128 from Inner Mongolia and 123 from Sichuan. Additional specimens of related or regionally occurring *Elymus* taxa from Qinghai were also examined, including *E. nutans* (350 specimens), *E. sibiricus* (242), *E. trachycaulus* (4), *E. breviaristatus* (49) and *E. atratus* (16). Thus, a total of 1,321 herbarium specimens were examined. For each specimen, we recorded whether the inflorescence showed lateral spike-bearing branchlets, secondary rachises or a hierarchical compound-spike organisation. Apparent spike complexity caused by overlapping spikes, awn spreading, flexuous rachises, leaf obstruction, specimen pressing or poor preservation was distinguished from true branched spikes.

### Flow-cytometric estimation of genome size and ploidy

Flow-cytometric estimation of genome size and ploidy was performed using fresh leaf tissues from *Elymus chaobotryus*, *E. multiramosus* and *E. sibiricus*. Nuclei isolated from fresh leaf material were analysed using a SONY SA3800 spectral flow cytometer. Maize inbred line B73 was used as the internal standard for genome-size estimation. For each sample, the mean fluorescence intensity (MFI) of the G0/G1 peak and the coefficient of variation (CV) were recorded. Genome size was calculated using the formula: sample genome size = sample G0/G1 MFI / maize B73 G0/G1 MFI × maize B73 genome size. Ploidy level was inferred from the relative fluorescence intensity and estimated genome size among the *Elymus* samples. The internal-standard estimates based on maize B73 were used as the final genome-size estimates in this study.

### Chromosome counting and FISH/GISH analysis

Chromosome counting and fluorescence in situ hybridization analyses were conducted using root-tip meristematic cells from cultivated plants of *Elymus chaobotryus*. Root tips were collected from actively growing seedlings and used for chromosome preparation. Metaphase chromosomes were stained with DAPI and observed under a fluorescence microscope equipped with a high-resolution CCD imaging system. Genomic in situ hybridization was performed using total genomic DNA of *Pseudoroegneria spicata* as a probe to identify St-genome chromosomes. Sequential fluorescence in situ hybridization was then conducted using Oligo-HvCSR and Oligo-pTa535 probes to distinguish additional chromosome groups.

### Plastome sequencing and genome annotation

Fresh leaf material from ten morphologically typical individuals of *Elymus chaobotryus* from the type population was pooled and used for total genomic DNA extraction. DNA extraction was performed by utilising the TianGen CTAB method kit. Sequencing libraries were prepared and quality-checked by PCR. Libraries were sequenced on an Illumina NovaSeq platform (150 bp paired-end). Reads were assembled with GetOrganelle v1.7.5, and the plastome was annotated using Geneious and GeSeq. The plastome sequence reported in this paper has been deposited in the GenBase at the National Genomics Data Center (CNCB-NGDC Members and Partners 2024), Beijing Institute of Genomics, Chinese Academy of Sciences/China National Center for Bioinformation, under accession number C_AA132757.1 that is publicly accessible at https://ngdc.cncb.ac.cn/genbase.

### Phylogenetic analysis

Complete plastome sequences were obtained for phylogenetic analysis, and the inverted repeat regions were removed prior to sequence alignment. The plastome sequences were aligned using MAFFT, and Gblocks was used to identify and retain conserved alignment blocks.

Following alignment, ModelFinder was used to identify the best-fit substitution model for the dataset. The GTR+F+R3 model was selected as the optimal model. Maximum-likelihood (ML) phylogenetic analysis was conducted in IQ-TREE (Nguyen et al. 2015) under the selected GTR+F+R3 model, with branch support assessed using 1,000 bootstrap replicates.

To determine the phylogenetic position of *Elymus chaobotryus* sp. nov., plastome sequences of 56 taxa were analysed (Suppl. material 2: table SS1). The dataset comprised 55 Poaceae taxa (including *Elymus* and representatives of other related genera) and one Solanaceae taxon (*Solanum tuberosum* L.). *Oryza sativa* L., *Zea mays* L., *Saccharum spontaneum* L., *Sorghum bicolor* (L.) Moench and *Solanum tuberosum* L. were used as outgroups.

## Results

### Taxonomic treatment

#### Elymus chaobotryus

Taxon classificationPlantaePoalesPoaceae

Y.C.Zhang sp. nov.

48D83719-28B7-5D86-B68E-E960A1D40603

urn:lsid:ipni.org:names:77397405-1

Figs 1, 2, 3, 4, 5, 6, 7, 8

##### Type.

China. Qinghai, Tongren City, Guashize Township. 35°29'11"N, 102°16'21"E, 3200 m a.s.l., 17 July 2025, *leg. Yong-Chao Zhang* 2019002 (holotype: HNWP 389653!).

**Figure 1.**
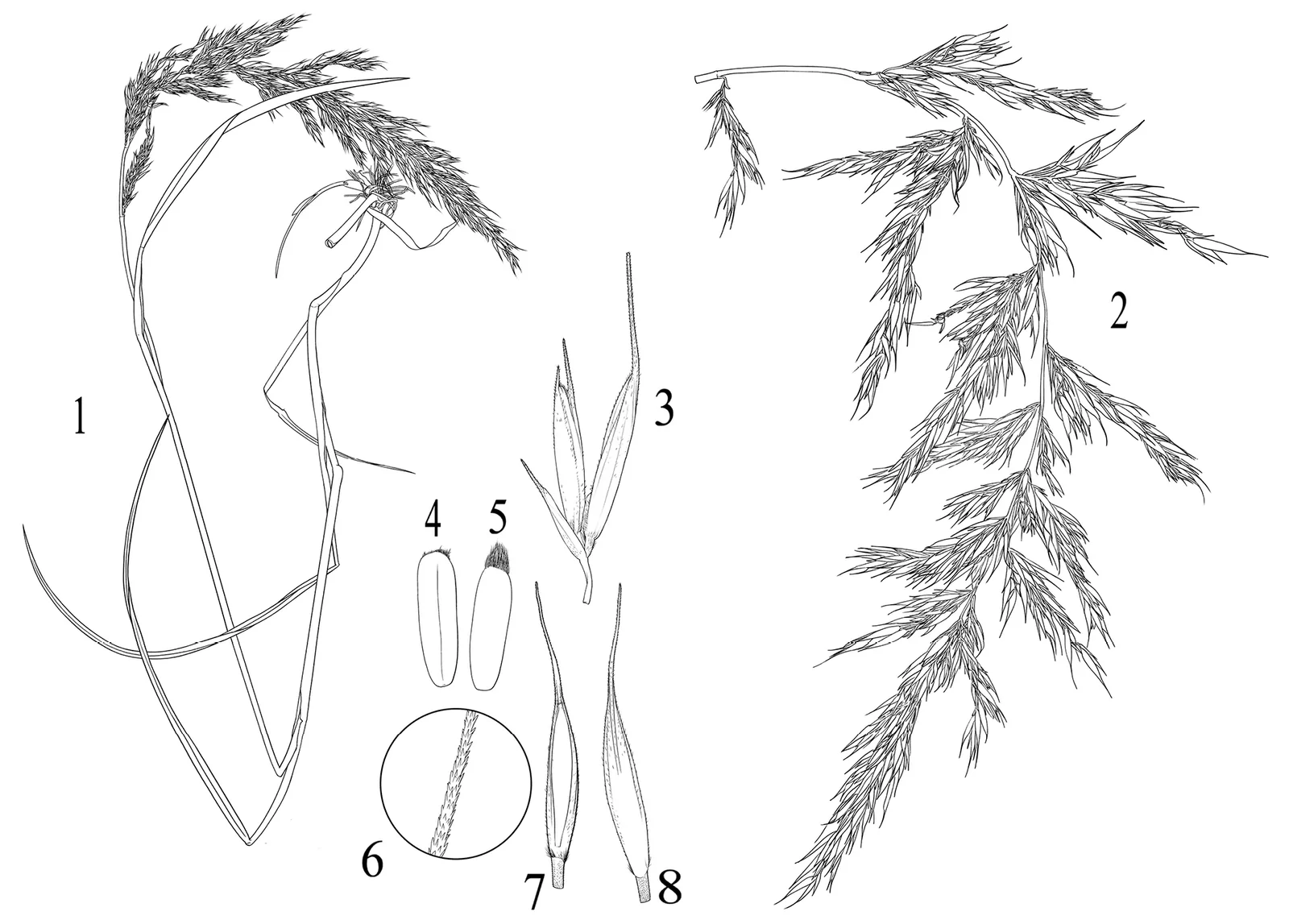
Illustration of *Elymus chaobotryus* Y.C. Zhang, sp. nov. (1) habit; (2) pendulous compound spike showing lateral spike-bearing branchlets; (3) spikelet; (4) caryopsis, dorsal view; (5) caryopsis, ventral view; (6) lemma awn; (7) lemma; (8) palea. Drawn by Yuan Liu.

##### Diagnosis.

*Elymus chaobotryus* resembles several *Elymus* species commonly occurring in the Qinghai–Tibet Plateau and northern China, particularly *E. multiramosus*, *E. sibiricus*, *E. nutans* and *E. dahuricus* var. dahuricus. These species share features such as tufted habit, erect culms, 1 or 2 spikelets per node and lanceolate glumes. *Elymus chaobotryus* can be readily distinguished by its unique pendulous compound spike, in which short lateral branchlets are produced from nearly all nodes of the main rachis and the lateral branches become progressively longer toward the base, giving the inflorescence a characteristic grape-like appearance.

**Figure 2.**
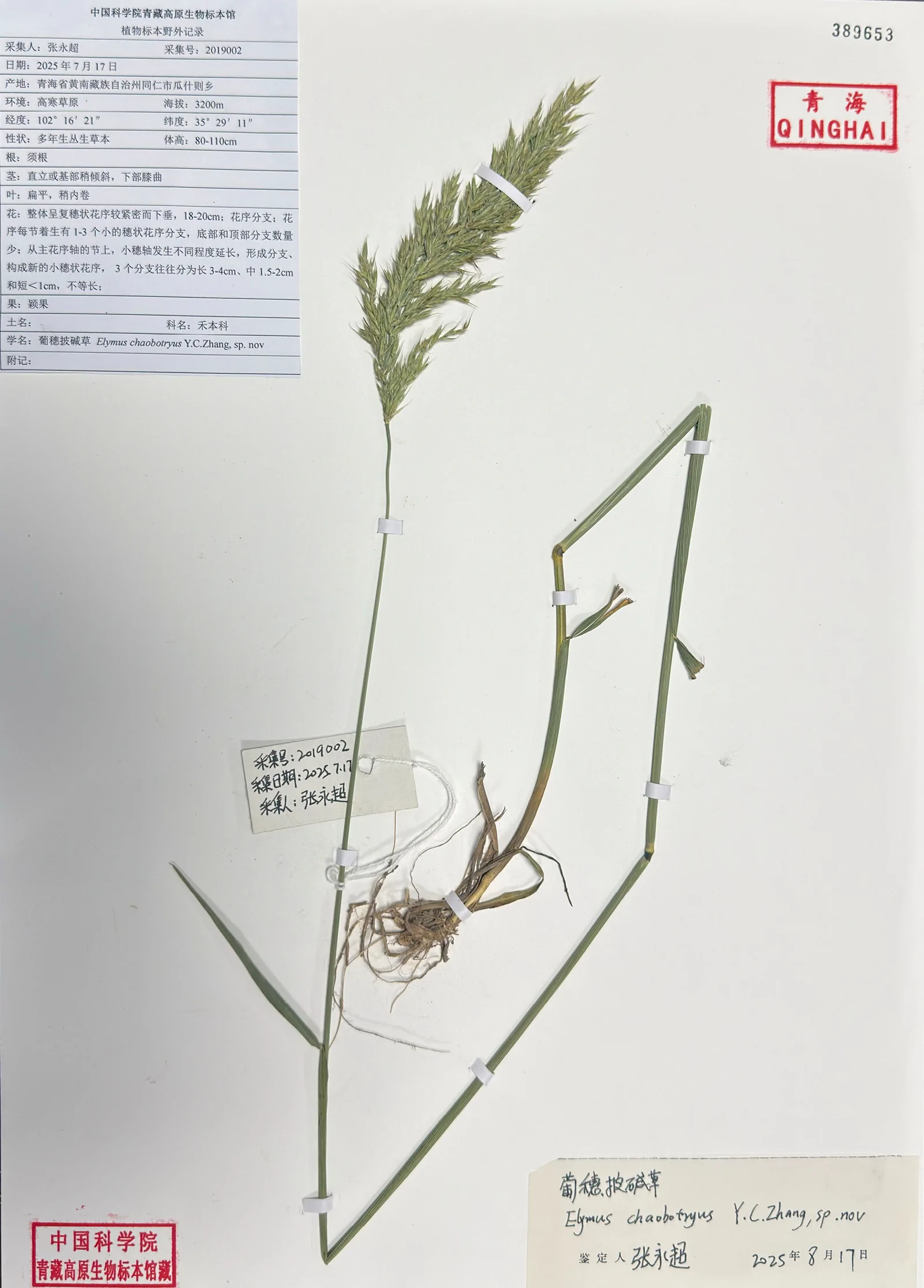
Holotype specimen of *Elymus chaobotryus* (389653), deposited in the Qinghai-Tibetan Plateau Museum of Biology (HNWP).

##### Description.

***Perennials***, tufted, without rhizomes. ***Culms*** 80–130 cm tall, erect, sometimes slightly decumbent at base; ***leaf sheaths*** open to the base, glabrous; ***ligules*** almost obsolete, represented by a very short membranous rim with shortly ciliate margins; ***blades*** 17–23 × 0.2–0.5 cm, flat, linear to narrowly lanceolate, green to grey-green, surfaces smooth or nearly smooth, sometimes slightly scabrous along margins and midrib; ***inflorescence*** 16–19 cm long, a pendulous compound spike; ***rachis*** slender, scabrous on angles, without cilia, rachis nodes dilated; ***branches***: lateral branches produced from nodes along almost the entire main rachis, from the lower part to near the apex. At each node, a single longer lateral branch (2–4 cm long, with 3–7 nodes) is typically associated with one or two shorter lateral branchlets (0.7–1.5 cm long), and/or sessile spikelets lacking branch elongation. The shorter branchlets are ascending and more or less appressed to the main axis, more numerous on the lower and middle nodes and sparser toward the apex; lateral branches become progressively longer from the apex toward the base of the inflorescence, giving a conspicuous grape-like appearance; ***spikelets*** usually 1 per branch node, occasionally 2 at the basal branch nodes, laterally compressed, containing 1–3 florets, disarticulating below the fertile florets at maturity; ***glumes*** 3–4 mm long and 0.4–0.6 mm wide, lanceolate, shorter than the first lemma, 3-veined, glabrous and scabrid along the veins, apices acute, usually bearing a mucro < 0.9 mm long; ***lemmas*** 5–7 mm long, lanceolate, awned, 3-veined (veins obscure near the base), midvein prominently raised, surfaces scabrous or sparsely puberulent toward the apex and margins; ***awns*** 3–4 mm long, slightly shorter than the lemma body, densely covered with small bristles on the surface; ***paleas*** about as long as the lemmas, keeled, ciliolate along both keels, sparsely puberulent between the keels; ***flowers*** perfect, lodicules 2, stamens 3; ***anthers*** 1.1–1.4 mm long, yellow; ***caryopsis*** narrowly rectangular-ellipsoid, 3.8–4.2 × 1.0–1.2 mm (L × W), adherent to lemma and palea; ***embryo*** 0.7–0.9 mm long; ***ventral sulcus*** narrow, shallow, linear, extending nearly the full length.

**Figure 3.**
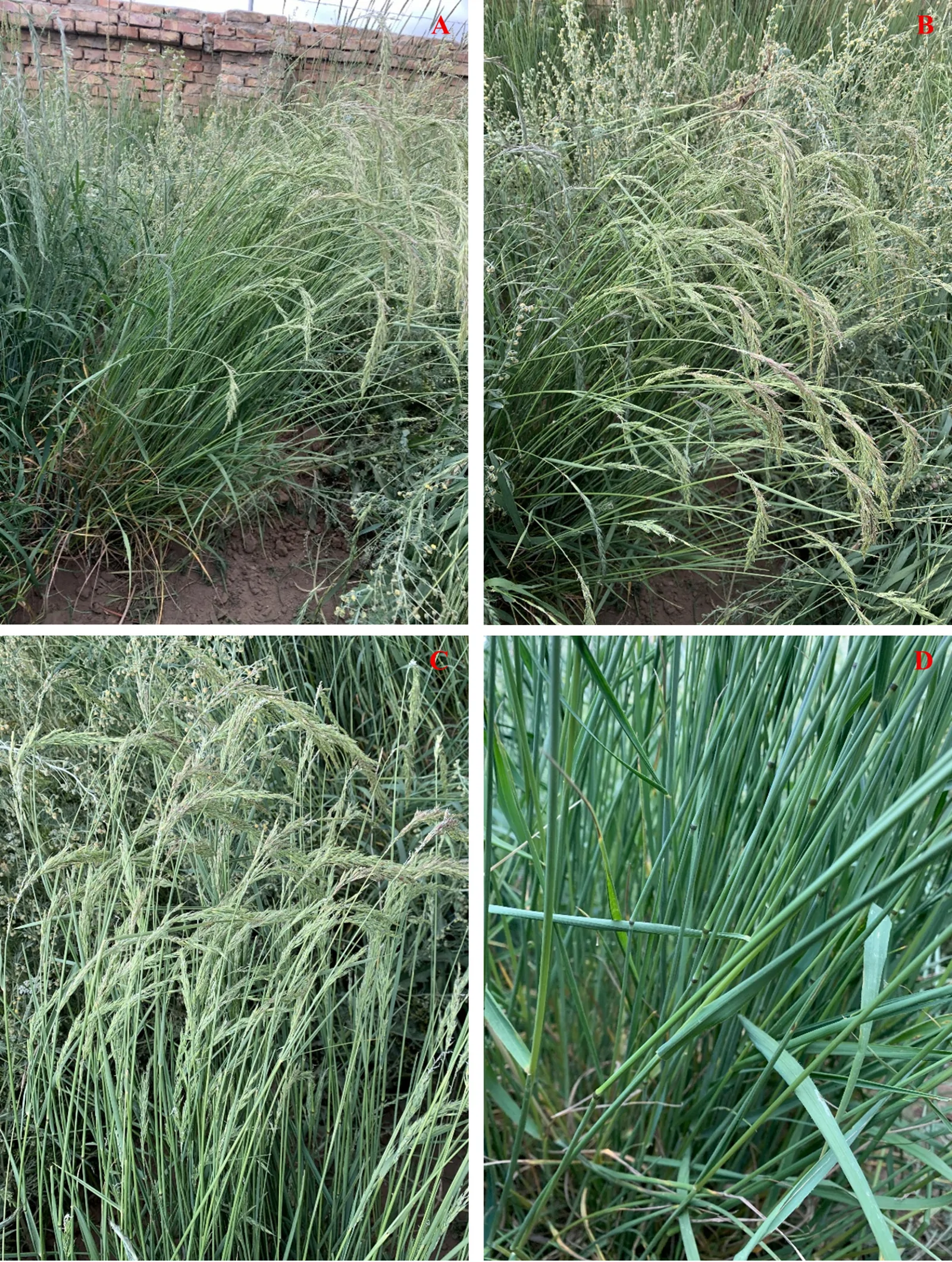
*Elymus chaobotryus* in the wild. **A**. Habit of a clump-forming plant; **B, C**. Close-ups of the pendulous compound spikes, showing short lateral branchlets bearing spikelets; **D**. Culms and leaves. Photographs by Yong-Chao Zhang.

**Figure 4.**
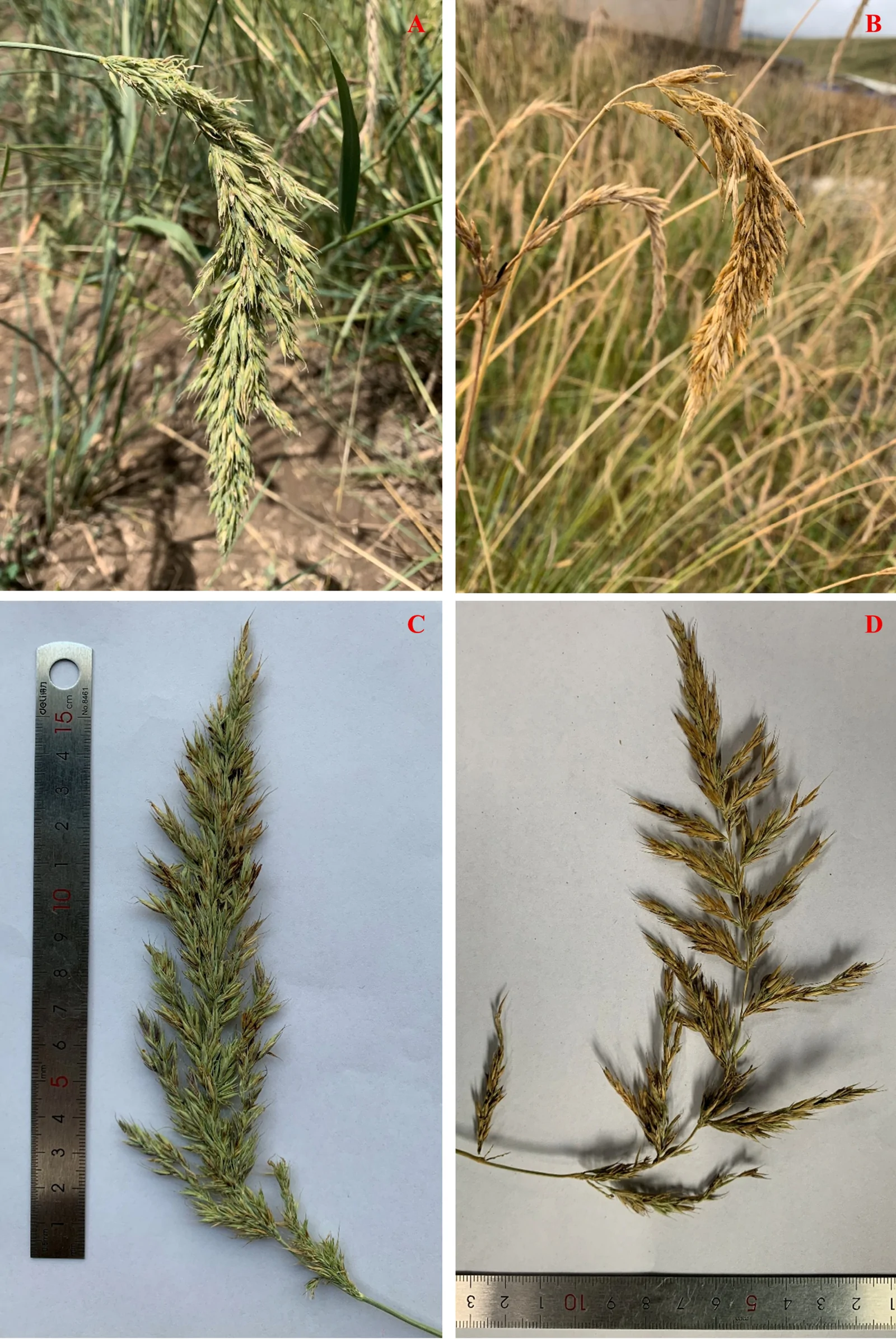
Pendulous compound spikes of *Elymus chaobotryus* from field-grown (cultivated) plants. **A**. Compound spike at anthesis; **B**. Compound spike at maturity; **C**. Excised compound spike with ruler (cm) showing overall size; **D**. Excised compound spike with lateral branchlets gently splayed to display the branching pattern and their insertion along the main rachis (ruler in cm).

##### Phenology.

*Elymus chaobotryus* flowers in late August and fruits in late September.

##### Etymology.

The specific epithet *chaobotryus* is a compound of *chao* (from the given name of the collector, Yongchao Zhang) and *botryus*, a Latinized form of the Greek *botrys* (“a bunch/cluster of grapes”), referring to the branched, grape-like compound spikes bearing numerous spikelets.

**Figure 5.**
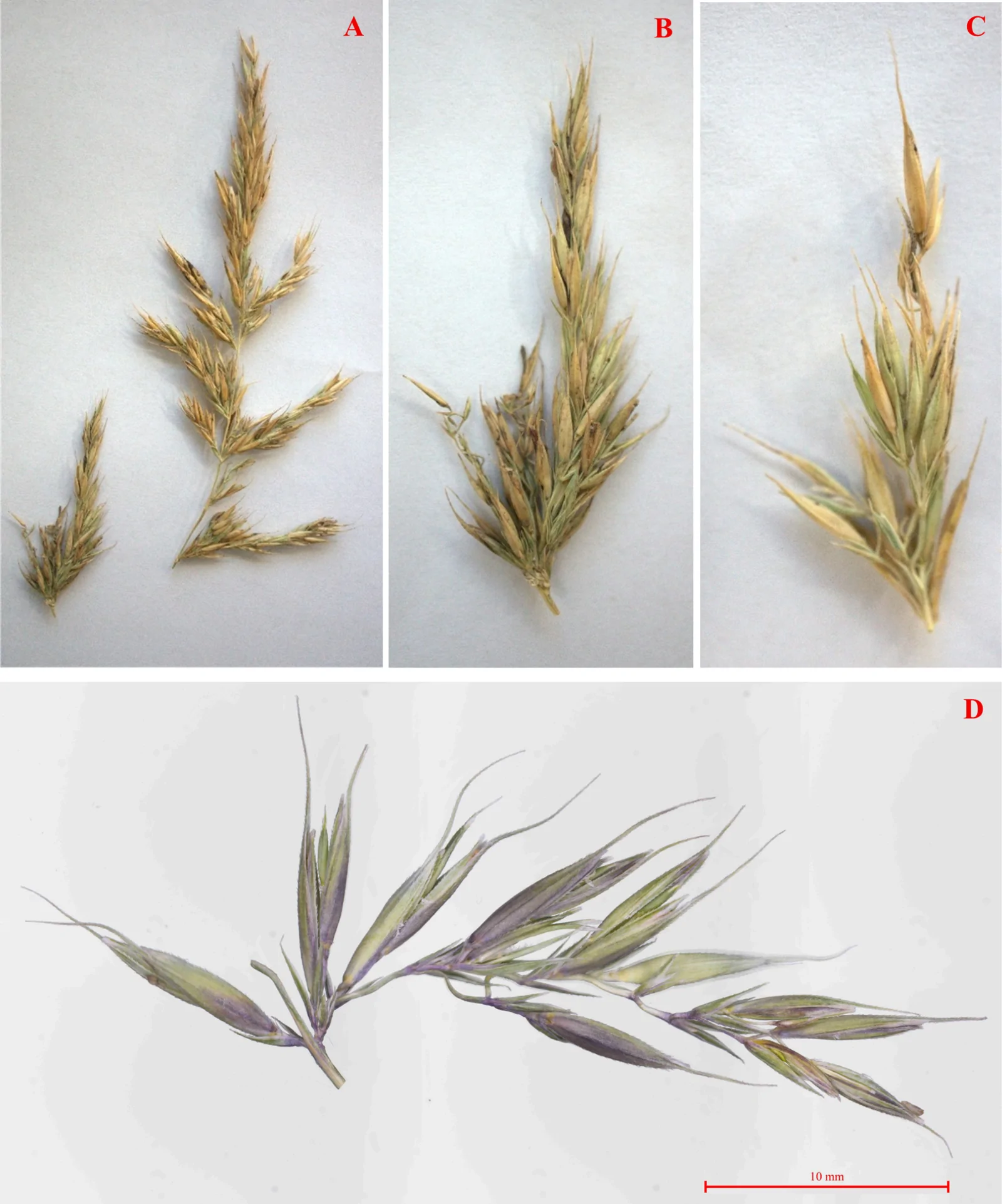
Structure of the compound spike in *Elymus chaobotryus*. **A**. Portion of a compound spike showing a node with a lateral branchlet; **B, C**. Close-up views of lateral branchlets bearing several spikelets; **D**. An excised node from the main rachis, showing one elongated lateral branchlet together with two sessile spikelets that lack branchlet elongation.

##### Vernacular name.

Pú Suì Pī Jiǎn Cǎo (Chinese pronunciation); 葡穗披碱草 (Chinese name).

##### Comparative notes.

***Inflorescence***. Inflorescence pendulous, forming a compound spike 16–19 cm long, with 10–15 short lateral branchlets (2–4 cm long) produced from nearly all nodes of the main rachis; the lateral branches become progressively longer toward the base of the inflorescence, giving the whole spike a slightly lax, characteristic grape-like appearance. In *Elymus multiramosus*, the compound spike has far fewer branches (3–6 per inflorescence) and appears more compact, whereas *E. sibiricus*, *E. nutans* and *E. dahuricus* var. dahuricus bear simple, unbranched spikes that lack any such lateral branchlets (Table 1).

**Figure 6.**
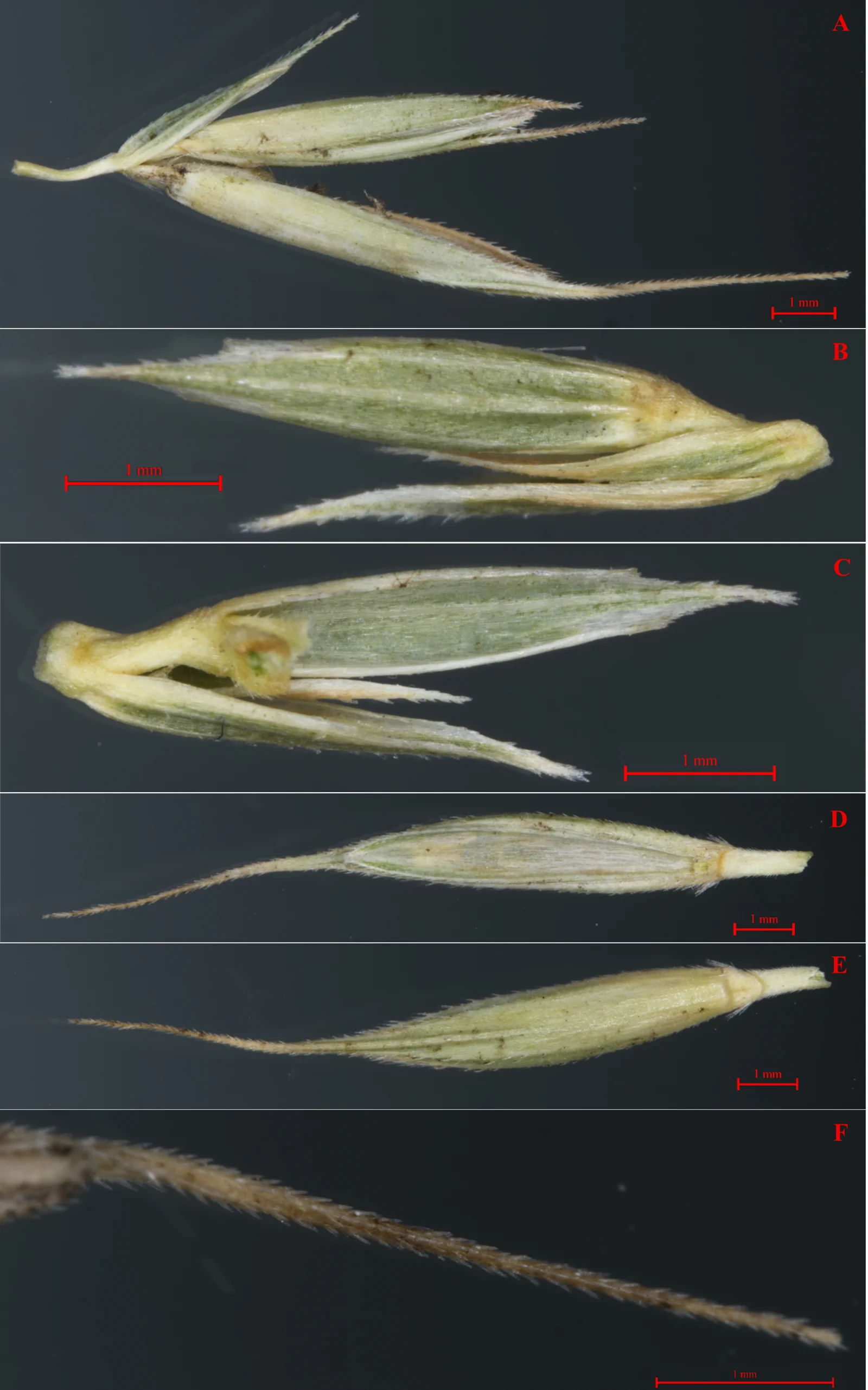
Spikelet morphology of *Elymus chaobotryus*. **A**. Spikelet, lateral view, showing the paired glumes enclosing the florets and an awned lemma; **B**. Glumes, abaxial (outer) faces; **C**. Glumes, adaxial (inner) faces; **D**. Palea; **E**. Lemma with distal awn; **F**. Awn, enlarged detail.

**Figure 7.**
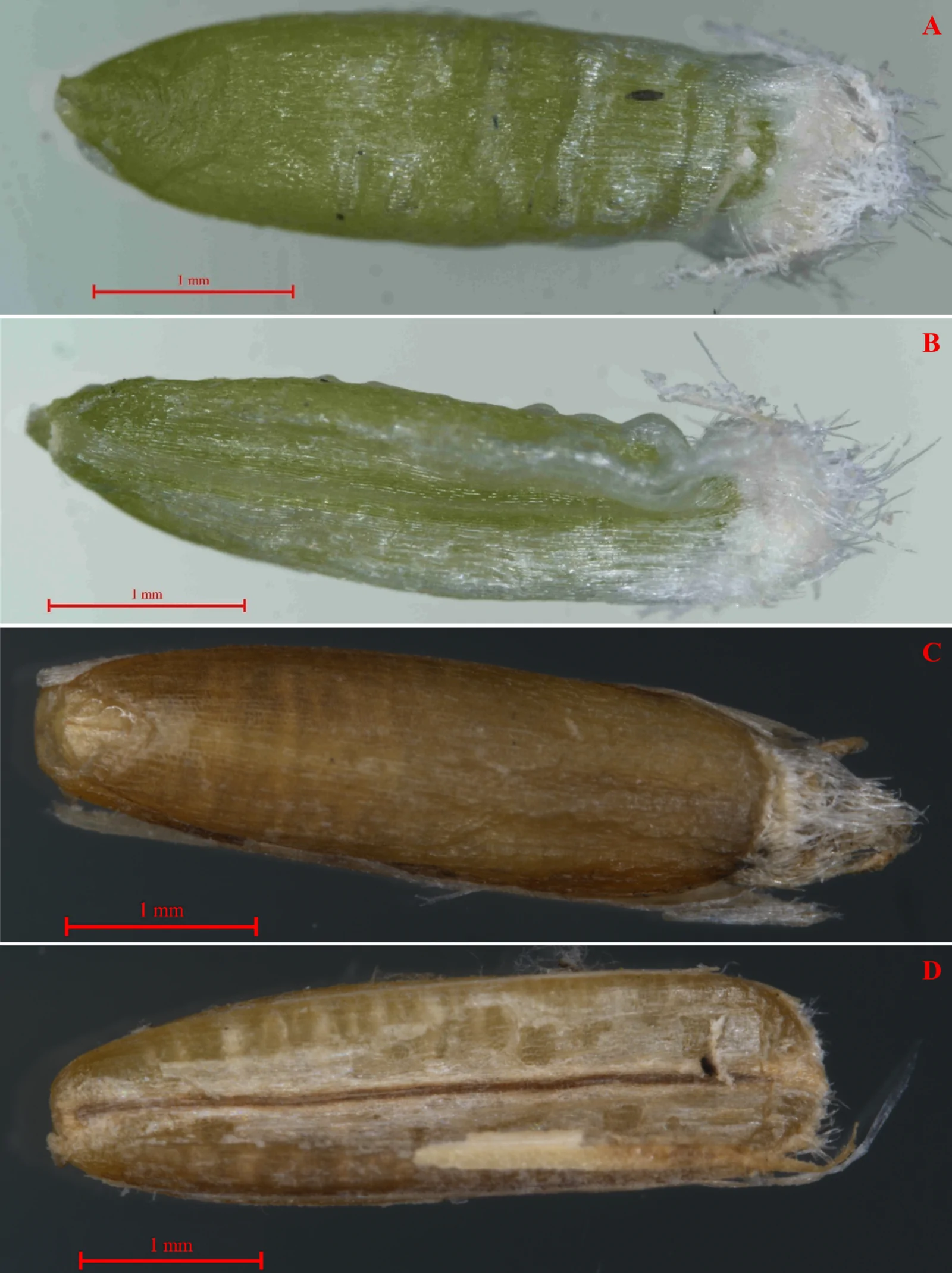
Caryopsis (grain) of *Elymus chaobotryus* at two developmental stages. **A, B**. Grain-filling stage: **A**. Dorsal side; **B**. Ventral side showing the ventral groove; **C, D**. Mature stage: **C**. Dorsal side; **D**. Ventral side with a conspicuous ventral groove.

**Figure 8.**
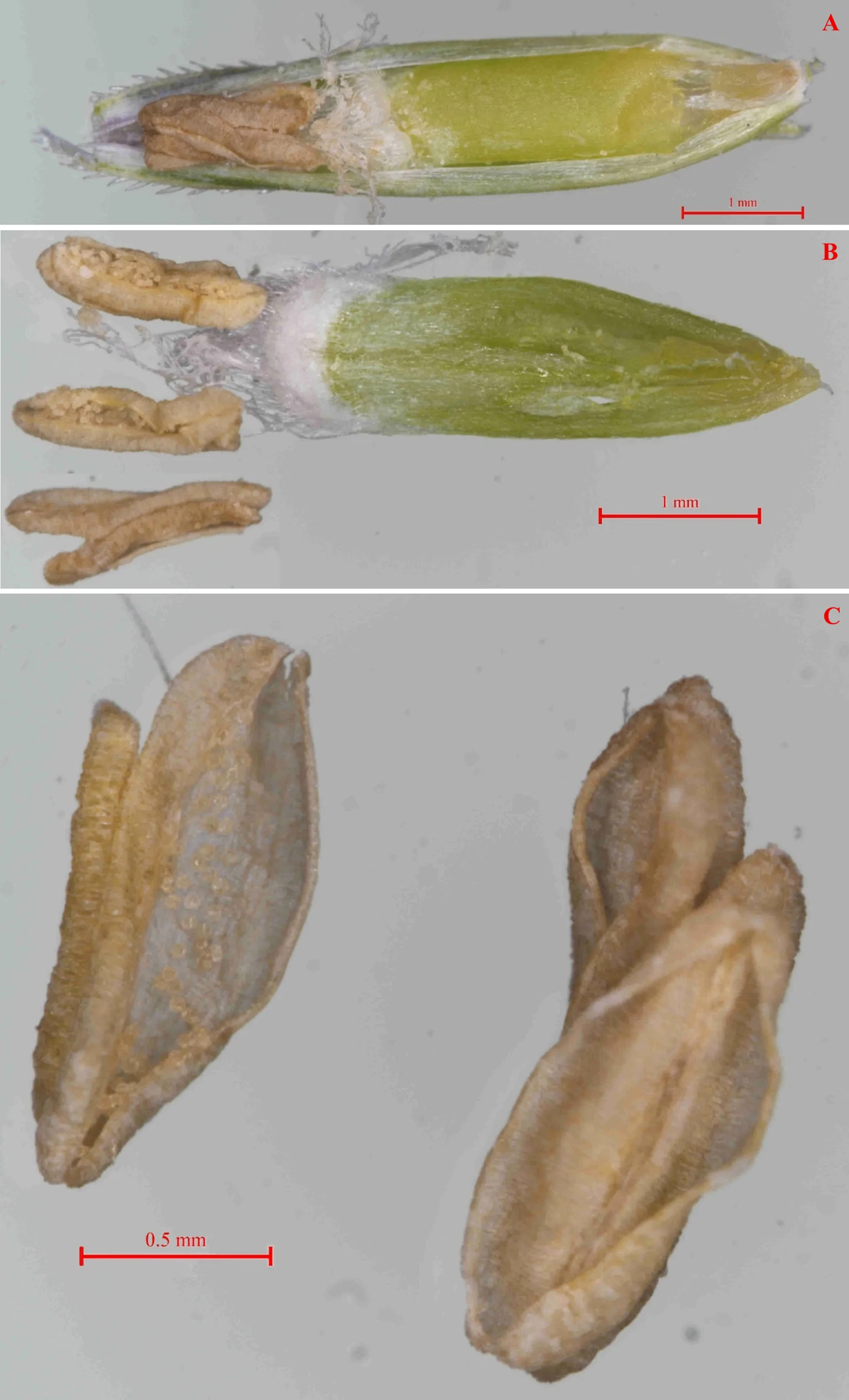
Florets of *Elymus chaobotryus* during grain filling. **A**. Floret with the palea removed (lemma retained), exposing the developing caryopsis (ovary) and basal lodicules; parts of the pistil and stamens visible; **B**. A different floret with both lemma and palea removed, showing the pistil (ovary with two plumose stigmas) and three stamens with slender filaments; **C**. Anther, close-up, dorsifixed and dehiscing by longitudinal slits, with pollen grains visible.

**Table 1.** Comparison of inflorescence characters of *Elymus chaobotryus* and related taxa.

**Species**	**Inflorescence**					
	**Type**	**Tightness**	**Upright or Bent**	**Total Length (cm)**	**Branch number**	**Branch length (cm)**
*Elymus chaobotryus* Y.C. Zhang	compound spike	slightly lax	pendulous	16–19	10–15	2–4
*Elymus multiramosus* Y.C. Zhang	compound spike	slightly lax	pendulous	17–19	3–6	2–4
*Elymus breviaristatus* (Keng) Keng f.	Spike	lax	tender and pendulous	10–15	no	no
*Elymus sinosubmuticus* S. L. Chen	Spike	laxer	curved	3.5–7.5	no	no
*Elymus sibiricus* L.	Spike	laxer	pendulous	15–20	no	no
*Elymus atratus* (Nevski) Hand.-Mazz.	Spike	denser	flexuous, pendulous	5–8	no	no
*Elymus nutans* Griseb.	Spike	denser	flexuous, apex pendulous	5–12	no	no
*Elymus canadensis* L.	Spike	denser	pendulous	12–20	no	no
*Elymus villifer* C. P. Wang & H. L. Yang	Spike	denser	slightly curved	9–12	no	no
*Elymus purpuraristatus* C. P. Wang & H. L. Yang	Spike	denser	erect or slightly curved	8–15	no	no
*Elymus dahuricus* Turcz. var. *violeus* C. P. Wang & H. L. Yang	Spike	denser	curved	18.5–25.5	no	no
*Elymus dahuricus* Turcz. var. dahuricus	Spike	denser	erect	14–18	no	no
*Elymus excelsus* Turcz.	Spike	laxer	erect	15–22	no	no
*Elymus dahuricus* var. *cylindricus* Franch.	Spike	denser	erect	7–14	no	no
*Elymus tangutorum* (Nevski) Hand.-Mazz.	Spike	denser	erect	8–15	no	no
*Elymus dahuricus* Turcz.	Spike	denser	erect	14–18	no	no
*Elymus barystachyus* L. B. Cai	Spike	denser	erect	8–18	no	no
*Elymus ciliaris* (Trin. ex Bunge) Tzvelev	Spike	denser	erect or slightly pendulous	10–20	no	no
*Elymus grandis* (Keng) S. L. Chen	Spike	laxer	erect	20–25	no	no
*Elymus uzbekistanicus* Usupbaev & Alieva	Spike	denser	erect	10–17	no	no
*Elymus nevskii* Tzvelev	Spike	denser	erect	–	no	no
*Elymus fedtschenkoi* Tzvelev	Spike	denser	erect or slightly pendulous	7–16	no	no
*Elymus macrochaetus* (Nevski) Tzvelev	Spike	denser	erect or slightly pendulous	c. 13	no	no
*Elymus burchan-buddae* (Nevski) Tzvelev	Spike	denser	pendulous	6–10	no	no
*Elymus pendulinus* (Nevski) Tzvelev	Spike	denser	pendulous	14–20	no	no
*Elymus pulanensis* (H. L. Yang) S. L. Chen	Spike	laxer	curved	8–10	no	no
*Elymus purpurascens* (Keng) S. L. Chen	Spike	denser	pendulous	13–15	no	no
*Roegneria yenchiana* X.Fan & L.N.Sha	Spike	denser	inclined to nodding	10–26	no	no

***Spikelets and glumes***. Spikelets usually 1 per branch node, occasionally 2 at the basal branch nodes, laterally compressed, containing 1–3 florets, disarticulating below the fertile florets at maturity. Glumes lanceolate, shorter than the first lemma, 3–4 mm long and c. 0.4–0.6 mm wide, 3-veined, glabrous but scabrid along the veins, with acute apices that usually bear a mucro less than 0.9 mm long. In *Elymus multiramosus*, the spikelets are also 1–2 per node, but the glumes are distinctly longer (4–7 mm) and their apical awns are much longer (1.5–2.2 mm). *E. sibiricus* has narrowly lanceolate glumes 4–5 mm long with 3–5 veins and a conspicuous short awn to about 4 mm, whereas in *E. nutans* the glumes are oblong, 3–4 mm long, 3–4-veined, with acuminate or shortly awned apices (awn 1–4 mm). *E. dahuricus* var. dahuricus differs even more in having lanceolate to linear-lanceolate glumes 8–10 mm long with 3–5 veins and well-developed awns about 5 mm long. Thus, the combination in *E. chaobotryus* of lanceolate, relatively short glumes (3–4 mm), consistently 3-veined and with mucros (< 0.9 mm) is unique among these closely related species (Table 2).

**Table 2.** Comparison of spikelet number and glume morphology of *Elymus chaobotryus* and related taxa.

**Species**	**Spikelet per node**	**Glume**				
		**Shape**	**Length (mm)**	**No. of veins**	**Apex morphology**	**Awn/mucro length (mm)**
*Elymus chaobotryus* Y.C. Zhang	1 (occasionally 2)	lanceolate	3–4	3	acute, usually mucronate	< 0.9
*Elymus multiramosus* Y.C. Zhang	1–2	lanceolate	4–7	3	awned	1.5–2.2
*Elymus breviaristatus* (Keng) Keng f.	2	oblong or ovate lanceolate	3–4	1–3	acuminate or mucronate	1
*Elymus sinosubmuticus* S. L. Chen	1–2	oblong	2–3	3	acute or acuminate, not mucronate	absent
*Elymus sibiricus* L.	1–2	narrowly lanceolate	4–5	3–5	acuminate or shortly awned	4
*Elymus atratus* (Nevski) Hand.-Mazz.	2	narrowly oblong or lanceolate	2–4	1–3	acuminate	<1
*Elymus nutans* Griseb.	1–2	oblong	3–4	3–4	acuminate or shortly awned	1–4
*Elymus canadensis* L.	2–3	linear	3–4	3–4	awned	7–18
*Elymus villifer* C. P. Wang & H. L. Yang	1–2	narrowly lanceolate	4.5–7.5	3–4	acuminate to an awned tip	1.5–2.5
*Elymus purpuraristatus* C. P. Wang & H. L. Yang	2	lanceolate to linear-lanceolate	7–10	3	mucronate	1
*Elymus dahuricus* Turcz. var. *violeus* C. P. Wang & H. L. Yang	1–2	lanceolate	7–11	3–5	awned	3–6
*Elymus dahuricus* Turcz. var. dahuricus	1–2	lanceolate or linear-lanceolate	8–10	3–5	awned	5
*Elymus excelsus* Turcz.	2–4	narrowly lanceolate	10–13	5–7	awned	7
*Elymus dahuricus* var. *cylindricus* Franch.	1–2	lanceolate to linear-lanceolate	7–8	3–5	acuminate	4
*Elymus tangutorum* (Nevski) Hand.-Mazz.	1–2	lanceolate to linear-lanceolate	7–10	5	acuminate	1–3
*Elymus dahuricus* Turcz.	1–2	lanceolate to linear-lanceolate	7–10	3–5	acuminate or awned	5
*Elymus barystachyus* L. B. Cai	2	linear-lanceolate	7–10	4–7	acuminate or pointed	1.5
*Elymus ciliaris* (Trin. ex Bunge) Tzvelev	1	oblong-lanceolate	7–9	5–7	acuminate or shortly mucronate	<1
*Elymus grandis* (Keng) S. L. Chen	1	oblong-lanceolate	10–15	5–7	acute	–
*Elymus uzbekistanicus* Usupbaev & Alieva	1	lanceolate	8–11	5–7	acuminate	–
*Elymus nevskii* Tzvelev	1	broadly lanceolate	12–15	7–9	acute, usually 1-dentate	no
*Elymus fedtschenkoi* Tzvelev	1	broadly lanceolate	c. 13	–	acute to acuminate	no
*Elymus macrochaetus* (Nevski) Tzvelev	1	narrowly lanceolate	10–20	–	narrowly acute	to 3
*Elymus burchan-buddae* (Nevski) Tzvelev	1	ovate-lanceolate	3–5	3–5	acute, unawned	no
*Elymus pendulinus* (Nevski) Tzvelev	1	oblong-lanceolate	7–10	5–7	acute to long-acuminate	no
*Elymus pulanensis* (H. L. Yang) S. L. Chen	1	oblong-lanceolate	4.5–7	3–5	awned	1.5–5
*Elymus purpurascens* (Keng) S. L. Chen	1	oblong-lanceolate	5–8	3–5	acute	–
*Roegneria yenchiana* X. Fan & L. N. Sha	1	rectangular	3–8	3–5	acute, usually 1-toothed	–

***Lemmas and paleas***. In *Elymus chaobotryus*, the lemmas are lanceolate, relatively short, 5–7 mm long, 3-veined (the veins obscure near the base), with the midvein prominently raised and the surfaces scabrous or sparsely puberulent toward the apex and along the margins; the awns arise from the lemma apex, 3–4 mm long, slightly shorter than the lemma body and densely covered with fine bristles. Paleas are about as long as the lemmas, keeled, ciliolate along both keels, sparsely puberulent between the keels, with the apex rounded or slightly flattened. In *E. multiramosus* the lemmas are distinctly longer (7–10 mm) but still 3-veined and bear much longer awns (9–12 mm) that clearly exceed the lemma body, while the palea apex is likewise rounded or flattened. *E. sibiricus* has 5-veined lemmas 8–11 mm long with awns 10–15 mm and paleas with a conspicuously 2-lobed apex; *E. nutans* possesses oblong-lanceolate, 5-veined lemmas about 10 mm long with awns 12–20 mm, and its paleas have obtuse-rounded or truncate apices. *E. dahuricus* var. dahuricus differs further in having 5-veined lemmas c. 9 mm long with long awns 10–20 mm and paleas with truncate apices. Thus, the combination in *E. chaobotryus* of comparatively short, 3-veined lemmas (5–7 mm) with short awns (3–4 mm), together with paleas bearing a simple rounded or flattened apex, clearly separates it from these allied species (Table 3).

**Table 3.** Comparison of palea-apex and lemma characters of *Elymus chaobotryus* and related taxa.

**Species**	**Palea apex**	**Lemmas**			
		**Type**	**Vein number**	**First lemma length (mm)**	**Awn length (mm)**
*Elymus chaobotryus* Y.C. Zhang	rounded or flattened	lanceolate	3	5–7	3–4
*Elymus multiramosus* Y.C. Zhang	rounded or flattened	lanceolate	3	7–10	9–12
*Elymus breviaristatus* (Keng) Keng f.	obtuse-rounded or slightly concave	lanceolate	5	8–9	1–5
*Elymus sinosubmuticus* S. L. Chen	obtuse-rounded	lanceolate	5	7–8	2
*Elymus sibiricus* L.	2-lobed	lanceolate	5	8–11	10–15
*Elymus atratus* (Nevski) Hand.-Mazz.	obtuse-rounded	lanceolate	5	7–8	10–17
*Elymus nutans* Griseb.	obtuse-rounded or truncate	oblong-lanceolate	5	10	12–20
*Elymus canadensis* L.	pointed or obtusely rounded and retuse	lanceolate	5	10–17	20–30
*Elymus villifer* C. P. Wang & H. L. Yang	–	oblong-lanceolate	5	7–11	
*Elymus purpuraristatus* C. P. Wang & H. L. Yang	–	oblong-lanceolate		6–9	7–15
*Elymus dahuricus* Turcz. var. *violeus* C. P. Wang & H. L. Yang	–	lanceolate		9–21	9–21
*Elymus dahuricus* Turcz. var. dahuricus	truncate	lanceolate	5	9	10–20
*Elymus excelsus* Turcz.	–		5	8–12	15–40
*Elymus dahuricus* var. *cylindricus* Franch.	obtuse-rounded	lanceolate	5	7–8	6–13
*Elymus tangutorum* (Nevski) Hand.-Mazz.	obtuse-headed	lanceolate	5	8–12	3–11
*Elymus dahuricus Turcz*.	narrowly truncate	lanceolate		7–9	2–20
*Elymus barystachyus* L. B. Cai	–	oblong-lanceolate		7–8	1–2
*Elymus ciliaris* (Trin. ex Bunge) Tzvelev	obtuse-headed	oblong-lanceolate	5	8–9	10–30
*Elymus grandis* (Keng) S. L. Chen	truncate or obtuse-rounded, densely pubescent	lanceolate	5	15	1
*Elymus uzbekistanicus* Usupbaev & Alieva	truncate or obtuse-rounded	lanceolate		8–9	to 25
*Elymus nevskii* Tzvelev	truncate or subobtuse, densely pubescent	lanceolate	–	11–13	1–5
*Elymus fedtschenkoi* Tzvelev	obtuse	lanceolate	–	13–18	20–40
*Elymus macrochaetus* (Nevski) Tzvelev	–	lanceolate	–	c. 11	15–30
*Elymus burchan-buddae* (Nevski) Tzvelev	obtuse	lanceolate	5	c. 10	20–25
*Elymus pendulinus* (Nevski) Tzvelev	–	lanceolate	–	9–11	15–28
*Elymus pulanensis* (H. L. Yang) S. L. Chen	–	lanceolate	5	11	30–40
*Elymus purpurascens* (Keng) S. L. Chen	–	lanceolate	5	9–11	18–28
*Roegneria yenchiana* X. Fan & L. N. Sha	truncate	oblong-lanceolate to oblong	3	9–12	17–25

***Leaf sheaths and blades***. In *Elymus chaobotryus*, the leaf sheaths are completely glabrous and the blades are flat, linear to narrowly lanceolate, 17–23 cm long and 2–5 mm wide, green to grey-green, with surfaces smooth or nearly smooth and only slightly scabrous along the margins and midrib; the ligules are almost obsolete, represented by a very short membranous rim with shortly ciliate margins. In *E. multiramosus* the leaf sheaths are likewise glabrous and the blades are also flat, but they are distinctly broader (18–22 cm × 5–7 mm). *E. sibiricus* has smooth and glabrous sheaths and flattened blades 10–20 cm long and 5–10 mm wide, while in *E. nutans* the blades, although flattened, are shorter and broader (6–8 cm × 3–5 mm) and often sparsely pilose above and scabrous or smooth below. *E. dahuricus* var. dahuricus differs even more in having smooth and glabrous sheaths and long, flattened blades 15–25 cm long and 5–12 mm wide, sometimes becoming slightly involute. Thus, among these allied species, *E. chaobotryus* is characterized by its combination of glabrous sheaths and conspicuously narrower, almost smooth leaf blades (2–5 mm wide) (Table 4).

**Table 4.** Comparison of leaf-sheath and leaf-blade characters of *Elymus chaobotryus* and related taxa.

**Species**	**Leaf-sheath type**	**Leaf-blade morphology**	**Leaf length (cm)**	**Leaf width (mm)**
*Elymus chaobotryus* Y.C. Zhang	glabrous	blade flattened	17–23	2–5
*Elymus multiramosus* Y.C. Zhang	glabrous	blade flattened	18–22	5–7
*Elymus breviaristatus* (Keng) Keng f.	glabrous	blade flattened	4–12	3–5
*Elymus sinosubmuticus* S. L. Chen	glabrous	blade flattened or involute	3–6	1.5–3
*Elymus sibiricus* L.	smooth and glabrous	blade flattened	10–20	5–10
*Elymus atratus* (Nevski) Hand.-Mazz.	smooth and glabrous	blade or involute	3–10	2
*Elymus nutans* Griseb.	glabrous	blade flattened, sparsely pilose above, scabrous or smooth below	6–8	3–5
*Elymus canadensis* L.	glabrous	blade flattened	20–30	7–15
*Elymus villifer* C. P. Wang & H. L. Yang	densely villous	flattened or margins involute	9–15	3–6
*Elymus purpuraristatus* C. P. Wang & H. L. Yang	glabrous	blades often involute	15–25	2.5–4
*Elymus dahuricus* Turcz. var. *violeus* C. P. Wang & H. L. Yang	base densely white villous	blade flattened or drying involute	20–35	8.7–13.6
*Elymus dahuricus* Turcz. var. dahuricus	smooth and glabrous	blade flattened, sparsely involute	15–25	5–12
*Elymus excelsus* Turcz.	glabrous	flattened	20–30	10–16
*Elymus dahuricus* var. *cylindricus* Franch.	glabrous	blade flattened	5–12	5
*Elymus tangutorum* (Nevski) Hand.-Mazz.	smooth	blade flattened	10–20	6–14
*Elymus dahuricus* Turcz.	glabrous, or densely pilose at base	blade glaucous, flat, rarely rolled	5–25	5–12
*Elymus barystachyus* L. B. Cai	glabrous	blade glabrous on both surfaces	7–22	4–8
*Elymus ciliaris* (Trin. ex Bunge) Tzvelev	glabrous, rarely shortly pilose at base	blade flattened	10–20	3–10
*Elymus grandis* (Keng) S. L. Chen	glabrous	blade flattened, subcoriaceous, smooth or slightly scabrous above	20–30	to 10
*Elymus uzbekistanicus* Usupbaev & Alieva	densely pilose	blades convolute or more or less flat, densely long-hairy on both surfaces	–	1–4
*Elymus nevskii* Tzvelev	glabrous, sometimes lower sheaths softly pilose	blade flattened, sparsely hairy; adaxial surface scabrous, abaxial surface glabrous or scabrous	–	7–11
*Elymus fedtschenkoi* Tzvelev	glabrous or hairy	blade flattened, green, adaxially glabrous or with scattered long hairs along ribs	–	3–10
*Elymus macrochaetus* (Nevski) Tzvelev	scabrous	blade flattened, adaxially densely hairy	–	3–9
*Elymus burchan-buddae* (Nevski) Tzvelev	glabrous	blade flattened or margins involute	5–9	1.5–3
*Elymus pendulinus* (Nevski) Tzvelev	lower sheaths retrorsely hairy, upper glabrous	blade flattened, glabrous or sparsely pilose above	10–20	5–9
*Elymus pulanensis* (H. L. Yang) S. L. Chen	–	blade flattened or involute	7–10	1.5–3
*Elymus purpurascens* (Keng) S. L. Chen	glabrous	blades involute, firm	11–22	3–6
*Roegneria yenchiana* X. Fan & L. N. Sha	glabrous	blade flattened	27–40	4–6

***Culms***. *Elymus chaobotryus* is perennial and tufted, without rhizomes; the culms are erect, sometimes slightly decumbent at the base and 80–130 cm tall. *E. multiramosus* shares the tufted habit and culms that are erect with a slightly decumbent base, but the plants are generally shorter, with culm height only 82–95 cm. In *E. sibiricus*, the culms are solitary or sparsely tufted, erect or with the base slightly inclined and 60–90 cm tall, whereas *E. nutans* has tufted culms 50–70 cm tall with the bases slightly geniculate rather than decumbent. In *E. dahuricus* var. dahuricus and its allied varieties the culms are erect and reach 70–140 cm, but the tufts are usually more sparsely developed. Some other related species, such as *E. purpuraristatus* and *E. dahuricus* var. *violeus*, are much taller, with stout, erect culms up to 160–225 cm. Thus, the combination in *E. chaobotryus* of a tufted, non-rhizomatous habit with culms erect, only slightly decumbent at the base and of intermediate height (80–130 cm) distinguishes it from both the shorter, weakly tufted species and the markedly taller robust species in the complex (Table 5).

**Table 5.** Comparison of culm morphology of *E. chaobotryus* and related taxa.

**Species**	**Growth habit**	**Culm type**	**Culm height (cm)**
*Elymus chaobotryus* Y.C. Zhang	tufted	erect, base slightly decumbent	80–130
*Elymus multiramosus* Y.C. Zhang	tufted	erect, base slightly decumbent	82–95
*Elymus breviaristatus* (Keng) Keng f.	sparsely tufted	erect or basally geniculate, short, decurrent rhizomes	70
*Elymus sinosubmuticus* S. L. Chen	tufted	erect or base slightly geniculate, weak	25–45
*Elymus sibiricus* L.	solitary or sparsely tufted	erect or base slightly inclined	60–90
*Elymus atratus* (Nevski) Hand.-Mazz.	sparsely tufted	erect, weak	40–60
*Elymus nutans* Griseb.	tufted	erect, base slightly geniculate	50–70
*Elymus canadensis* L.	sparsely tufted	erect or base slightly geniculate	100
*Elymus villifer* C. P. Wang & H. L. Yang	sparsely tufted	erect	60–75
*Elymus purpuraristatus* C. P. Wang & H. L. Yang	tufted	erect, stout	160
*Elymus dahuricus* Turcz. var. *violeus* C. P. Wang & H. L. Yang	sparsely tufted	erect	145–225
*Elymus dahuricus* Turcz. var. dahuricus	sparsely tufted	erect	70–140
*Elymus excelsus* Turcz.	tufted	erect, robust	140
*Elymus dahuricus* var. *cylindricus* Franch.	tufted	erect, weak	40–80
*Elymus tangutorum* (Nevski) Hand.-Mazz.	tufted	erect, tall and stout, base geniculate	120
*Elymus dahuricus* Turcz.	tufted	erect, base geniculate	40–140
*Elymus barystachyus* L. B. Cai	laxly tufted or solitary	erect, base geniculate	50–80
*Elymus ciliaris* (Trin. ex Bunge) Tzvelev	solitary or sparsely tufted	erect, base geniculate	40–80
*Elymus grandis* (Keng) S. L. Chen	solitary or sparsely tufted	erect, with creeping rhizomes	80–100
*Elymus uzbekistanicus* Usupbaev & Alieva	densely tufted	erect, densely long-hairy	50–100
*Elymus nevskii* Tzvelev	–	erect, base geniculate	50–120
*Elymus fedtschenkoi* Tzvelev	–	erect, base slightly geniculate	50–100
*Elymus macrochaetus* (Nevski) Tzvelev	tufted	erect, densely shortly hairy below nodes	80–120
*Elymus burchan-buddae* (Nevski) Tzvelev	tufted	erect, partly slightly ascending	45–60
*Elymus pendulinus* (Nevski) Tzvelev	tufted	erect	60–80
*Elymus pulanensis* (H. L. Yang) S. L. Chen	sparsely tufted	erect	30–50
*Elymus purpurascens* (Keng) S. L. Chen	solitary or sparsely tufted	erect, base slightly inclined, rigid	60–90
*Roegneria yenchiana* X. Fan & L. N. Sha	tufted	erect	50–100

##### Distribution and habitat.

The species is presently known only from a small area in Guashize Township, Tongren City, southeastern Qinghai Province (35°29'11"N, 102°16'21"E), on the north-eastern Qinghai-Tibetan Plateau.

Field surveys documented seven local populations totaling ca. 36 individuals. Individuals were strongly tufted and typically produced more than 30 tiller**s**. All observed plants bore the distinctive pendulous compound spike, with short lateral branchlets present along most nodes of the main rachis.

*Elymus chaobotryus* was discovered in the corner of the walled courtyard of a herder’s house, at an elevation of 3200 m a.s.l., and surrounded by extensive alpine steppe. The herder family had temporarily left the homestead for seasonal pasture rotation. The enclosed courtyard may have provided a small refuge where the species could persist and reproduce, whereas in the open steppe its populations are likely subject to intense grazing pressure, which may help explain why its unusual branched inflorescences had long escaped notice.

Other plant species in the vicinity include *Carex parvula* O. Yano, *Carex alatauensis* S. R. Zhang, *Carex capillifolia* (Decne.) S. R. Zhang, *Elymus dahuricus* Turcz., *Dasiphora fruticosa* (L.) Rydb., *Poa araratica* Trautv., *Festuca sinensis* Keng ex E. B. Alexeev.

##### Additional observations.

The taxon was first noticed in the field in 2019, when seeds were collected. Since 2020, plants raised from these seeds have been grown continuously in a forage germplasm nursery near Qinghai Lake on the Qinghai-Tibet Plateau (ca. 3200 m a.s.l.). Across multiple years of cultivation, the diagnostic inflorescence architecture (a pendulous compound spike with lateral branchlets, producing a grape-like outline) has remained stable, and a seed stock has been built up. In 2025, replicated field plots were established in Haibei Tibetan Autonomous Prefecture (three counties; three replicates per county; 3 × 5 m per plot) using a sowing rate of 22.5 kg ha^–1^. The diagnostic inflorescence architecture remained stable in both nursery-grown and field-grown plants (Suppl. material 1).

##### Herbarium survey of related and co-occurring *Elymus* taxa.

None of the examined specimens of *E. dahuricus* or the other surveyed *Elymus* taxa possessed the compound-spike architecture observed in *E. chaobotryus* (Suppl. material 3: table SS2). Several specimens showed lax, flexuous, pendulous, or superficially branched-looking spikes; however, in all such cases the inflorescence retained a single continuous main rachis and lacked lateral spike-bearing branchlets and secondary rachises. The apparent branching was associated with loose spikelet arrangement, spreading awns, overlapping culms or spikes, leaf obstruction, or specimen pressing. Accordingly, no surveyed specimen showed the hierarchical compound-spike organisation characteristic of *E. chaobotryus*.

##### Genome size and ploidy estimation.

Flow-cytometric analysis yielded estimated genome sizes of 11.43 Gb for *Elymus chaobotryus*, 11.32 Gb for *E. multiramosus*, and 7.10 Gb for *E. sibiricus* (Table 6). Based on relative fluorescence intensity and genome-size comparison among the *Elymus* samples, *E. chaobotryus* and *E. multiramosus* were inferred to be hexaploid (6x), whereas *E. sibiricus* was inferred to be tetraploid (4x).

**Table 6.** Flow-cytometric estimates of genome size and inferred ploidy of *Elymus chaobotryus* and two related *Elymus* species using maize B73 as an internal standard.

**Germplasm**	**Reference**	**Fluorescence intensity of reference**	**Fluorescence intensity of germplasm**	**Ratio**	**Genome size (Gb)**	**Ploidy**
*Elymus chaobotryus*	maize B73	17530	84184	4.80	11.43	6x
*Elymus multiramosus*	maize B73	17497	83236	4.76	11.32	6x
*Elymus sibiricus*	maize B73	17617	52520	2.98	7.10	4x

##### Chromosome number and genome constitution.

**Figure 9.**
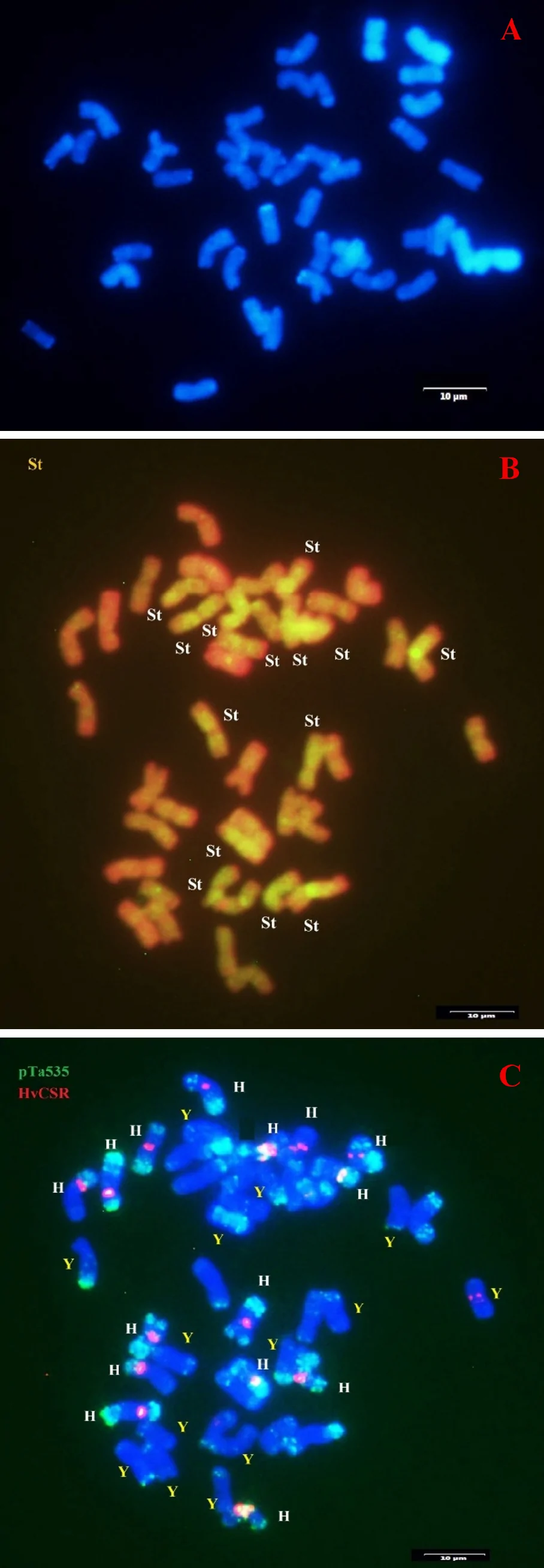
Chromosome counting, GISH and FISH analyses of *Elymus chaobotryus*. **A**. DAPI staining showing 2n = 42; **B**. GISH identifying St-genome chromosomes; **C**. Sequential FISH distinguishing chromosome groups interpreted as H and Y genomes. Scale bars: 10 μm.

Chromosome counts from DAPI-stained metaphase cells showed that the examined *Elymus chaobotryus* sample had 2n = 42 chromosomes (Fig. 9A). The chromosomes were approximately 5.0–8.0 μm long and were predominantly metacentric to submetacentric. Genomic in situ hybridization (GISH) using total genomic DNA of *Pseudoroegneria spicata* as a probe identified 14 St-genome chromosomes (Fig. 9B). Sequential FISH with Oligo-HvCSR and Oligo-pTa535 revealed distinct hybridization patterns among the remaining chromosomes, allowing them to be assigned to two groups interpreted as representing the H and Y genomes, with 14 chromosomes in each group (Fig. 9C). Together, these results support a hexaploid chromosome complement (2n = 6x = 42) in *E. chaobotryus* and are consistent with an StHY genomic constitution.

### Plastome characteristics

**Figure 10.**
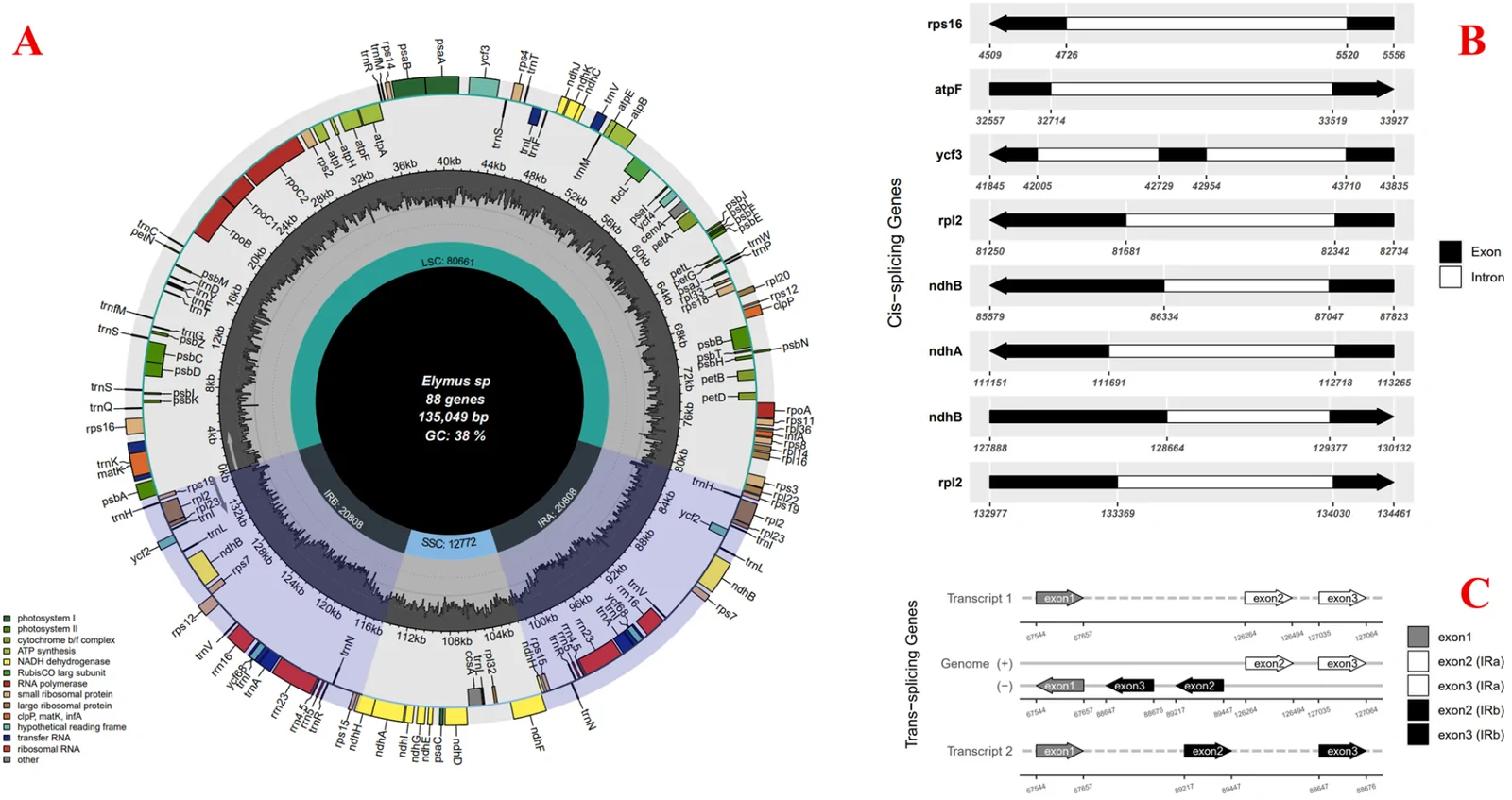
Overview of the plastome of *Elymus chaobotryus* (*135,049 bp*; *GC 38%; 88 genes*). **A**. Circular plastome map. The quadripartite structure comprises LSC 80,661 bp, SSC 12,772 bp, and two IRs of 20,808 bp each. From inner to outer tracks: GC-content plot and genes colour-coded by functional categories (legend); arrows indicate transcription orientation, with outer-ring and inner-ring genes transcribed clockwise and counter-clockwise, respectively; **B**. Cis-splicing genes showing exon-intron organisation and genomic coordinates: rps16, atpF, ycf3 (two introns), rpl2, ndhA, ndhB; genes located in the IRs (rpl2, ndhB) are displayed twice; **C**. Trans-splicing gene rps12: exon 1 in the LSC, exons 2–3 duplicated in IRa/IRb; two representative transcripts are illustrated.

The plastome of *Elymus chaobotryus* is 135,049 bp in length and has an overall GC content of 38%. It exhibits the typical quadripartite architecture, comprising an LSC region (80,661 bp), an SSC region (12,772 bp), and a pair of inverted repeats (IRa and IRb; 20,808 bp each) (Fig. 10A). A total of 88 genes were annotated (color-coded by functional category in the circular map); genes on the outer and inner tracks are transcribed clockwise and counter-clockwise, respectively. Cis-splicing genes include *rps16*, *atpF*, *rpl2*, *ndhA and ndhB*, with *ycf3* harbouring two introns; *rpl2* and *ndhB* are duplicated in the IRs (Fig. 10B). The trans-splicing gene rps12 has exon 1 in the LSC, whereas exons 2 and 3 are duplicated in IRa/IRb, with two representative transcripts illustrated (Fig. 10C).

### Phylogenetic analysis

**Figure 11.**
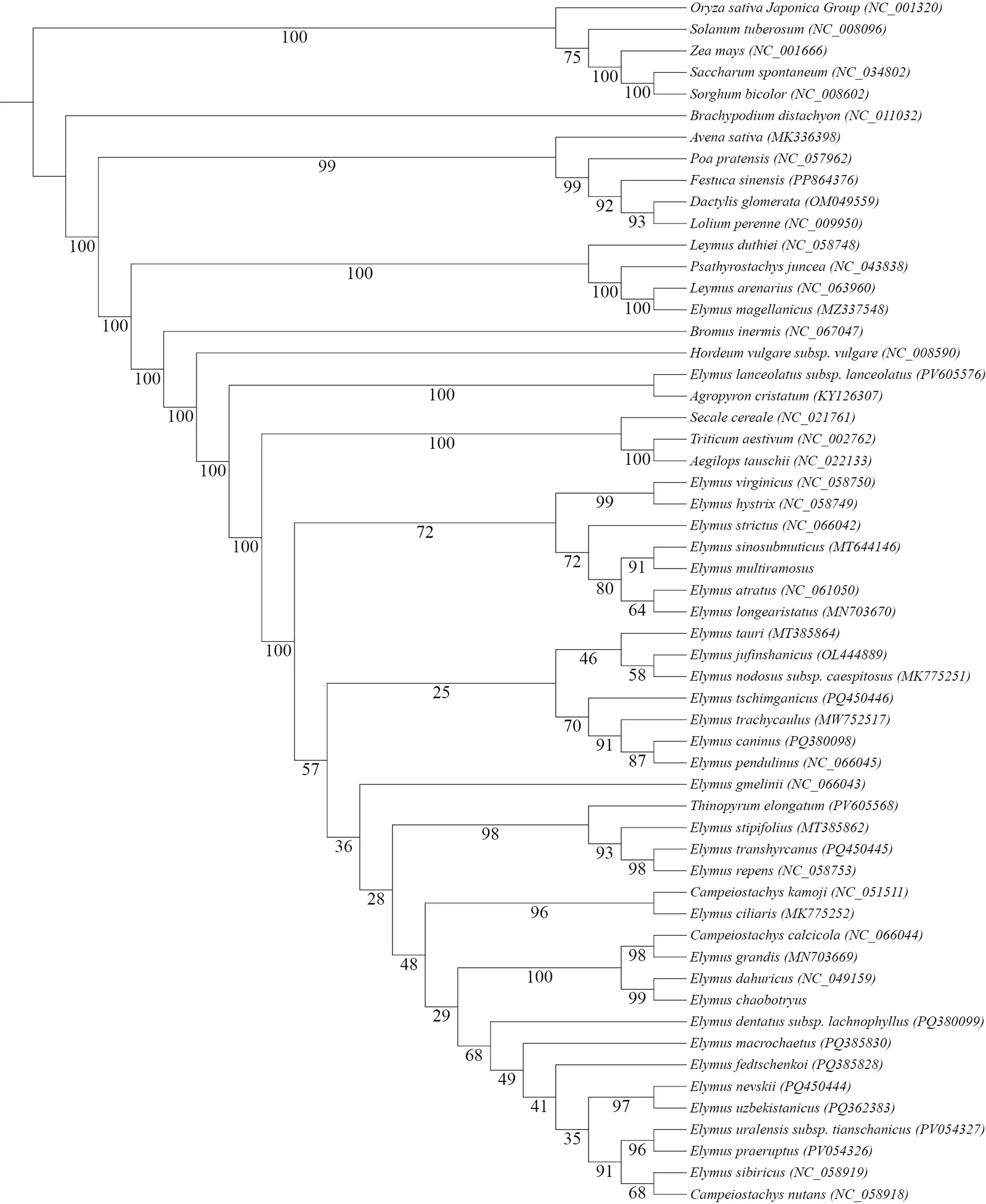
Maximum-likelihood (ML) plastome phylogeny of *Elymus* and related taxa, including *Elymus chaobotryus*, inferred using IQ-TREE under the GTR+F+R3 model from plastome sequences after removal of the inverted repeat regions. Branch support was assessed using 1,000 bootstrap replicates, and bootstrap support values (%) are shown at the nodes. *Oryza sativa*, *Zea mays*, *Saccharum spontaneum*, *Sorghum bicolor*, and *Solanum tuberosum* were used as outgroups.

*Elymus chaobotryus* is recovered as sister to *Elymus dahuricus*, and this pair is sister to a strongly supported clade comprising *E. grandis* and *Campeiostachys calcicola*. This assemblage is embedded within a broader plastome lineage that also includes *Campeiostachys kamoji* + *E. ciliaris*, *E. dentatus* subsp. *lachnophyllus*, *E. macrochaetus*, *E. fedtschenkoi*, *E. nevskii*, *E. uzbekistanicus*, *E. uralensis* subsp. *tianschanicus*, *E. praeruptus*, *E. sibiricus* and *Campeiostachys nutans*, although relationships along the backbone of this lineage receive only weak to moderate support. By contrast, *E. multiramosus* is placed in a different plastome lineage and forms a well-supported sister pair with *E. sinosubmuticus*; this pair is sister to the *E. atratus* and *E. longearistatus* pair, and together they form a clade with *E. strictus*. This larger clade is, in turn, sister to the *E. virginicus* and *E. hystrix* pair, with moderate support. Thus, *E. chaobotryus* and *E. multiramosus* are recovered in different plastome lineages rather than as a single plastome clade (Fig. 11).

## Discussion

### Taxonomic remarks

*Elymus chaobotryus* is a newly recognized member of *Elymus* s.l. from the north-eastern Qinghai-Tibetan Plateau (QTP). In overall habit, it resembles regional congeners compared here such as *E. multiramosus*, *E. sibiricus*, *E. nutans* and *E. dahuricus* var. dahuricus, but it can be readily distinguished by its inflorescence architecture in combination with a consistent set of associated morphological characters.

The most striking diagnostic feature of *E. chaobotryus* is its pendulous compound spike, in which numerous lateral spike-bearing branchlets arise from most nodes of the main rachis and form a clear hierarchical branching pattern. This distinctive architecture provides an immediately recognizable character that separates *E. chaobotryus* from the simple-spiked congeners considered here.

Among the taxa considered here, *E. multiramosus* provides the most direct morphological comparison because it also possesses a compound spike. However, *E. multiramosus* has far fewer branches and a more compact spike (Zhang et al. 2024a), whereas *E. chaobotryus* produces lateral branches from nearly all rachis nodes. By contrast, *E. sibiricus*, *E. nutans* and *E. dahuricus* var. dahuricus possess simple, unbranched spikes lacking lateral branchlets. These differences are pronounced, readily observable in the field, and stable across individuals, making inflorescence architecture a major diagnostic character for the new species.

Floral and vegetative characters provide additional support for the recognition of *Elymus chaobotryus*. Compared with *E. multiramosus*, *E. chaobotryus* is characterized by shorter, consistently 3-veined glumes with very short apical mucros, shorter 3-veined lemmas with relatively short awns, and narrower, nearly smooth leaf blades (Zhang et al. 2024a). These differences complement the distinctive compound-spike architecture and provide diagnostic information from multiple morphological structures. Thus, the recognition of *E. chaobotryus* does not rely on spike branching alone, but on a consistent combination of inflorescence, floral and vegetative characters that separate it from *E. multiramosus* and other morphologically similar *Elymus* taxa.

### Cytogenetic evidence

The agreement between flow-cytometric estimates and chromosome counts supports a hexaploid interpretation for *Elymus chaobotryus*. The GISH and sequential FISH patterns further provide cytogenetic evidence consistent with an StHY genomic constitution. Together, these results clarify the ploidy level and genomic constitution of *E. chaobotryus* and provide an additional cytogenetic perspective on its taxonomic characterization within *Elymus*.

### Plastome phylogeny

Plastome phylogenomics provides an independent perspective on the maternal evolutionary history of *Elymus chaobotryus*. Its placement within the broader *Elymus* complex and its close relationship to *E. dahuricus* are consistent with its taxonomic assignment based on morphology. However, plastome phylogeny reflects only the maternal lineage and therefore cannot by itself fully resolve the reticulate evolutionary history of polyploid *Elymus* (Mason-Gamer 2004; Fan et al. 2013). Particularly noteworthy is that *E. chaobotryus* and *E. multiramosus*, despite both possessing strongly branched inflorescences, are recovered in different plastome lineages. This pattern indicates that spike branching is not necessarily associated with close maternal relatedness and may have arisen independently in different *Elymus* lineages. Thus, spike branching remains informative for morphological diagnosis but should be interpreted cautiously in phylogenetic inference.

### Implications for inflorescence diversification and character use

Inflorescence architecture has long been important in Triticeae systematics and evolutionary interpretation (Benlloch et al. 2007; Kellogg 2022). Hypotheses in Triticeae and grasses more broadly suggest that the unbranched spike may represent a derived condition resulting from reduction of a more highly branched ancestral inflorescence (Vegetti and Anton 1995; Kellogg et al. 2013; Poursarebani et al. 2020). In this context, *E. multiramosus* (Zhang et al. 2024a) and *E. chaobotryus* provide two well-documented examples of strongly compound, extensively branched inflorescences in *Elymus* from the Qinghai-Tibetan Plateau. Together with previous studies of branched-inflorescence germplasm in alpine *Elymus* (Zhang et al. 2024b; Yu et al. 2024), these observations indicate that pronounced spike branching represents a recurrent component of inflorescence variation in Qinghai-Tibetan Plateau *Elymus*. Its taxonomic value is therefore greatest when used in combination with floral and vegetative characters, whereas its evolutionary interpretation requires independent phylogenetic and developmental evidence. Further investigation of the initiation and differentiation of lateral spike-bearing branchlets may help clarify the developmental basis and evolutionary origin of compound-spike architecture in *Elymus*.

Taken together, these findings refine the diagnostic and phylogenetic context of *Elymus chaobotryus*. When inflorescence branching is ignored, specimens run to the *E. sibiricus* couplet in *Flora Reipublicae Popularis Sinicae* (Tomus 9(3)), *Flora Qinghaiica* (Vol. 4) and *Flora Xizangica* (Vol. 5) (Kuo 1987: 6–15; Wu 1987: 168–175; Liu 1999: 97–103). Under the full character set adopted here, however, *E. chaobotryus* is most readily distinguished by the combination of its hierarchical compound-spike architecture and associated floral and vegetative characters, whereas plastome phylogenomics provides complementary evidence concerning its maternal phylogenetic placement. Accordingly, compound-spike architecture should be explicitly scored and incorporated into identification keys for *Elymus*.

## Supplementary Material

XML Treatment for Elymus chaobotryus

## Additional information

### Artificial Intelligence (AI) use

The authors accept full responsibility for the content of the manuscript, including the disclosure of any use of AI.

The following AI tools were used in the preparation of this manuscript:

**AI tools**: ChatGPT.

**Description**: We used AI-assisted language tools (ChatGPT) solely for English-language support, including improving grammar, spelling, punctuation, wording, and sentence structure, and for checking formatting consistency (e.g., italics of scientific names and abbreviation style).

### Author ORCIDs

Y.-C. Zhang https://orcid.org/0000-0003-4656-5608

### Data availability

All of the data that support the findings of this study are available in the main text or Supplementary Information.

## Supplementary materials

Supplementary material 1 Field cultivation of *Elymus chaobotryus* showing repeated expression of the diagnostic compound-spike phenotype under cultivated conditions Data type png Explanation note **A**. General view of a 3 × 5 m cultivated plot established from seed stock derived from the original field collection; the red outline indicates the cultivated *E. chaobotryus* plot at the flowering stage; **B–D**. Close-up views of field-grown *E. chaobotryus* plants, showing repeated occurrence of pendulous compound spikes with lateral spike-bearing branchlets. The plot was established at a sowing rate of 22.5 kg ha^–1^. https://binary.pensoft.net/file/1788969This dataset is made available under the Open Database License (http://opendatacommons.org/licenses/odbl/1.0/). The Open Database License (ODbL) is a license agreement intended to allow users to freely share, modify, and use this Dataset while maintaining this same freedom for others, provided that the original source and author(s) are credited. Yong-Chao Zhang, Sa Xiao, Xin-Yun Ma, Ji-Gui Chen, Yan Qin, Yong Liu, Zhi-Ying Guo, Yu-Qing Zhou, Dian-Zhi Zhao, Qing-Sheng Shan, Hai-Zhang Lu, Xiao-Xing Wei

Supplementary material 2 Taxa included in the plastome phylogenetic analysis of *Elymus chaobotryus* and related taxa Data type docx https://binary.pensoft.net/file/1788970This dataset is made available under the Open Database License (http://opendatacommons.org/licenses/odbl/1.0/). The Open Database License (ODbL) is a license agreement intended to allow users to freely share, modify, and use this Dataset while maintaining this same freedom for others, provided that the original source and author(s) are credited. Yong-Chao Zhang, Sa Xiao, Xin-Yun Ma, Ji-Gui Chen, Yan Qin, Yong Liu, Zhi-Ying Guo, Yu-Qing Zhou, Dian-Zhi Zhao, Qing-Sheng Shan, Hai-Zhang Lu, Xiao-Xing Wei

Supplementary material 3 Herbarium specimens examined for comparative assessment of *Elymus chaobotryus* Data type docx https://binary.pensoft.net/file/1788971This dataset is made available under the Open Database License (http://opendatacommons.org/licenses/odbl/1.0/). The Open Database License (ODbL) is a license agreement intended to allow users to freely share, modify, and use this Dataset while maintaining this same freedom for others, provided that the original source and author(s) are credited. Yong-Chao Zhang, Sa Xiao, Xin-Yun Ma, Ji-Gui Chen, Yan Qin, Yong Liu, Zhi-Ying Guo, Yu-Qing Zhou, Dian-Zhi Zhao, Qing-Sheng Shan, Hai-Zhang Lu, Xiao-Xing Wei
