# Supplementary material for: Elymus chaobotryus (Poaceae), a new species from the north-eastern Qinghai-Tibetan Plateau, China
Source: PhytoKeys. 2026 Sep 24;279:215–44. doi: 10.3897/phytokeys.279.181697 (PMC13631565; doi:10.3897/phytokeys.279.181697)
Supplement: Supplementary file 1 [file phytokeys-279-215_article-181697__-s002.docx]

**Supplementary Table S1**. Taxa and plastome sequences used in the phylogenetic analysis. Publicly available plastome sequences were obtained from NCBI GenBank and GenBase of the National Genomics Data Center (NGDC); accession numbers are provided in the table.

| Family | Genus | Species | Accession number | Origin |
| --- | --- | --- | --- | --- |
| Solanaceae | *Solanum* | *Solanum tuberosum* L. | NC008096 | Italy, Turin |
| Poaceae | *Oryza* | *Oryza sativa* L. | NC_001320.1 | Japan |
| Poaceae | *Zea* | *Zea mays* L. | NC001666 | USA |
| Poaceae | *Saccharum* | *Saccharum spontaneum* L. | NC034802 | China, Guangxi |
| Poaceae | *Sorghum* | *Sorghum bicolor* (L.) Moench | NC008602 | USA, Florida |
| Poaceae | *Brachypodium* | *Brachypodium distachyon* Roem. & Schult. | NC_011032.1 | USA |
| Poaceae | *Avena* | *Avena sativa* L. | MK336398.1 | China |
| Poaceae | *Poa* | *Poa pratensis* L. | NC_057962.1 | China, Qinghai |
| Poaceae | *Dactylis* | *Dactylis glomerata* L. | OM049559.1 | Russia, Moscow |
| Poaceae | *Lolium* | *Lolium perenne* L. | NC_009950.1 | USA |
| Poaceae | *Leymus* | *Leymus duthiei* (Stapf ex Hook.f.) Y.H.Zhou & H.Q.Zhang | NC_058748.1 | China |
| Poaceae | *Psathyrostachys* | *Psathyrostachys juncea* (Fisch.) Nevski | NC_043838.1 | China |
| Poaceae | *Leymus* | *Leymus arenarius* (L.) Hochst. | NC_063960.1 | China |
| Poaceae | *Elymus* | *Elymus magellanicus* (É.Desv.) Á.Löve | MZ337548.1 | China, Henan |
| Poaceae | *Bromus* | *Bromus inermis* Leyss. | NC_067047.1 | Russia, Moscow |
| Poaceae | *Hordeum* | *Hordeum vulgare* L. | NC_008590.1 | USA |
| Poaceae | *Elymus* | *Elymus lanceolatus* subsp. *lanceolatus* (Scribn. & J.G. Sm.) Gould | PV605576 | China, Inner Mongolia |
| Poaceae | *Agropyron* | *Agropyron cristatum* (L.) Gaertn. | KY126307.1 | China |
| Poaceae | *Secale* | *Secale cereale* L. | NC_021761.1 | USA |
| Poaceae | *Aegilops* | *Aegilops tauschii* Coss. | NC_022133.1 | Switzerland |
| Poaceae | *Triticum* | *Triticum aestivum* L. | NC_002762.1 | USA |
| Poaceae | *Elymus* | *Elymus virginicus* L. | NC_058750.1 | China, Sichuan |
| Poaceae | *Elymus* | *Elymus hystrix* L. | NC_058749.1 | China, Sichuan |
| Poaceae | *Elymus* | *Elymus longearistatus* (Boiss.) Tzvelev | MN703670.1 | China, Sichuan |
| Poaceae | *Elymus* | *Elymus atratus* (Nevski) Hand.-Mazz. | MT610373.1 | China, Sichuan |
| Poaceae | *Elymus* | *Elymus sinosubmuticus* S. L. Chen | MT644146.1 | China, Sichuan |
| Poaceae | *Elymus* | *Elymus multiramosus* Y.C. Zhang | C_AA070531 | China, Qinghai |
| Poaceae | *Elymus* | *Elymus tauri* (Boiss. & Balansa) Melderis | MT385864.1 | China, Qinghai |
| Poaceae | *Elymus* | *Elymus nodosus* subsp. *caespitosus* (K.Koch) Melderis | MK775251.1 | China, Sichuan |
| Poaceae | *Elymus* | *Elymus jufinshanicus* (C.P.Wang & H.L.Yang) S.L.Chen | OL444889 | China, Inner Mongolia |
| Poaceae | *Elymus* | *Elymus strictus* (Keng) S. L. Chen | MZ736600.1 | China, Sichuan |
| Poaceae | *Elymus* | *Elymus tschimganicus* Czrep. | PQ450446 | Uzbekistan |
| Poaceae | *Elymus* | *Elymus trachycaulus* (Link) Gould ex Shinners | MW752517.1 | China, Qinghai |
| Poaceae | *Elymus* | *Elymus caninus* L. | PQ380098 | Uzbekistan, Tashkent |
| Poaceae | *Elymus* | *Elymus pendulinus* (Nevski) Tzvelev | NC_066045.1 | China, Sichuan |
| Poaceae | *Elymus* | *Elymus sibiricus* L. | MK775250.1 | China |
| Poaceae | *Elymus* | *Elymus nutans* Griseb. | NC_058918.1 | China |
| Poaceae | *Elymus* | *Elymus uralensis* subsp. *tianschanicus* (Drob.) Tzvelev | PV054327 | Uzbekistan |
| Poaceae | *Elymus* | *Elymus praeruptus* Tzvelev | PV054326 | Uzbekistan |
| Poaceae | *Elymus* | *Elymus gmelinii* (Ledeb.) Tzvelev | NC_066043.1 | China |
| Poaceae | *Thinopyrum* | *Thinopyrum elongatum* (Host) D.R.Dewey | PV605568.1 | China, Hohhot |
| Poaceae | *Elymus* | *Elymus stipifolius* (Trautv.) Melderis | MT385862 | China |
| Poaceae | *Elymus* | *Elymus transhyrcanus* (Nevski) Tzvelev | PQ 450445 | Uzbekistan |
| Poaceae | *Elymus* | *Elymus repens* (L.) Gould | NC_058753.1 | China |
| Poaceae | *Elymus* | *Elymus dahuricus* Turcz. ex Griseb. | NC_049159.1 | China |
| Poaceae | *Elymus* | *Elymus grandis* (Keng) S. L. Chen | MN703669.1 | China |
| Poaceae | *Festuca* | *Festuca sinensis* Keng ex E. B. Alexeev | PP864376.1 | China |
| Poaceae | *Campeiostachys* | *Campeiostachys calcicola* (Keng) Y.H.Zhou, H.Q.Zhang & M.Q.Deng | NC_066044.1 | China |
| Poaceae | *Campeiostachys* | *Campeiostachys kamoji* (Ohwi) B.R.Baum, J.L.Yang & C.Yen | NC_051511.1 | China |
| Poaceae | *Elymus* | *Elymus ciliaris* (Trin.) Tzvelev | MK775252.1 | China |
| Poaceae | *Elymus* | *Elymus dentatus* subsp. *lachnophyllus* (Ovcz. & Sidor.) Tzvelev | PQ380099 | Uzbekistan |
| Poaceae | *Elymus* | *Elymus macrochaetus* (Nevski) Tzvelev | PQ385830 | Uzbekistan |
| Poaceae | *Elymus* | *Elymus fedtschenkoi* Tzvelev | PQ385828 | Uzbekistan |
| Poaceae | *Elymus* | *Elymus nevskii* Tzvelev | PQ450444 | Uzbekistan |
| Poaceae | *Elymus* | *Elymus uzbekistanicus* Usupbaev & K.B. Alieva | PQ362383 | Uzbekistan |
