# Supplementary material for: Elymus chaobotryus (Poaceae), a new species from the north-eastern Qinghai-Tibetan Plateau, China
Source: PhytoKeys. 2026 Sep 24;279:215–44. doi: 10.3897/phytokeys.279.181697 (PMC13631565; doi:10.3897/phytokeys.279.181697)
Supplement: Supplementary file 2 [file phytokeys-279-215_article-181697__-s003.docx]

**Supplementary Table S2**. Herbarium specimens examined for comparative assessment of *Elymus chaobotryus*

| ID | Taxon label | Region | Herbarium/database | Branched spike | Similarity to E. c. | Transitional morphology | notes |
| --- | --- | --- | --- | --- | --- | --- | --- |
| PE02005508 | *Elymus dahuricus* Turcz. | Xinjiang | PE | No | No | No | Typical unbranched spike architecture; no lateral branchlets or compound spike tendency observed |
| PE01664856 | *Elymus dahuricus* Turcz. | USSR / Mongolia | PE | No | Partial | Weakly suggestive | Loose spike architecture with minor irregularity, but no lateral branchlets or compound spike framework observed |
| PE01521671 | *Elymus dahuricus* Turcz. | Shanxi, China | PE | No | Partial | Weakly suggestive | Loosely arranged spikelets and slightly irregular rachis architecture, but no secondary rachis or compound spike framework observed |
| PE01027483 | *Elymus dahuricus* Turcz. | Sichuan, China | PE | No | No | No | Single-axis, relatively lax spike; spikelets arranged along a single rachis; no lateral branchlets, secondary rachis, or compound spike organization observed. |
| PE00489142 | *Elymus dahuricus* Turcz. | Tibet, China | PE | No | Partial | Weakly suggestive | Relatively lax and elongated single-axis spike with minor irregularity in spikelet arrangement, but no lateral branchlets, secondary rachis, or compound spike organization observed. |
| PE00489132 | *Elymus dahuricus* Turcz. | Tibet, China | PE | No | Partial | Weakly suggestive | Flexuous and partially flattened single-axis spike with locally irregular spikelet arrangement, but lacking distinct lateral branchlets, secondary rachis, or hierarchical compound spike organization. |
| PE00489123 | *Elymus dahuricus* Turcz. | Qinghai, China | PE | No | No | No | Very slender and lax single-axis spike with widely spaced spikelets, but lacking lateral branchlets, secondary rachis, or compound spike organization. |
| PE00489120 | *Elymus dahuricus* Turcz. | Yunnan, China | PE | No | No | No | Typical unbranched single-axis spike with orderly spikelet arrangement; no lateral branchlets, secondary rachis, or compound spike organization observed. |
| PE00489119 | *Elymus dahuricus* Turcz. | Yunnan, China | PE | No | Partial | No | Strongly elongated and flexuous single-axis spike; apparent terminal separation likely caused by overlapping leaf tissue and specimen compression. No distinct lateral branchlets, secondary rachis, or compound spike hierarchy observed. |
| PE00489117 \| | *Elymus dahuricus* Turcz. | Yunnan, China | PE | No | No | No | Compact to moderately contracted single-axis spike; spikelets densely arranged toward the upper portion of the rachis, but no lateral branchlets, secondary rachis, or compound spike organization observed. |
| PE00489097 | *Elymus dahuricus* Turcz. | Yunnan, China | PE | No | No | No | Elongated and flexuous single-axis spike with slightly spreading spikelets; overall architecture remains unbranched, lacking lateral branchlets, secondary rachis, or hierarchical compound spike organization. |
| PE00489087 | *Elymus dahuricus* Turcz. | Sichuan, China | PE | No | No | No | Lax and pendulous single-axis spike with elongated awns and somewhat spreading spikelets; overall architecture remains strictly unbranched, with no lateral branchlets, secondary rachis, or compound spike framework observed. |
| PE00489084 | *Elymus dahuricus* Turcz. | Sichuan, China | PE | No | No | No | Extremely lax and slender single-axis spike with widely spaced spikelets and depauperate appearance; despite strong reduction and elongation, no lateral branchlets, secondary rachis, or hierarchical branching structure observed. |
| PE00489083 | *Elymus dahuricus* Turcz. | Sichuan, China | PE | No | Partial | Weakly suggestive | Compact and relatively robust single-axis spike with densely arranged spikelets; although superficially more crowded in appearance, no lateral branchlets, secondary rachis, or hierarchical branching structure observed. |
| PE00489069 | *Elymus dahuricus* Turcz. | Sichuan, China | PE | No | No | Weakly | Extremely elongated and flexuous spike with lax spikelet arrangement and strongly relaxed rachis architecture; however, all spikelets remain directly inserted on a single continuous rachis, with no evidence of lateral branch formation or secondary axes. Leaf overlap partially obscures the inflorescence and may create a false impression of branching. |
| PE00489062 | *Elymus dahuricus* Turcz. | Sichuan, China | PE | No | No | No | Dense and thickened single-axis spike with compact spikelet arrangement. All spikelets are directly attached to a continuous primary rachis; no lateral spike-bearing branches or secondary axes observed. |
|  | *Elymus dahuricus* Turcz. |  |  |  |  |  |  |
| PE00489048 | *Elymus dahuricus* Turcz. | Xinjiang, China | PE | No | No | Moderate | Flexuous and lax single-axis spike with partially disorganized spike apex and irregular spikelet orientation. The apical region gives a pseudo-branched appearance due to overlapping structures and compression artifacts, but all spikelets remain attached to a continuous primary rachis, with no secondary axes or true lateral branches observed. |
| PE00489045 | *Elymus dahuricus* Turcz. | Xinjiang, China | PE | No | Partial | Weakly suggestive | Single spike with slightly loose spikelets; basal portion shows minor deviation in lemma morphology; overall retains typical unbranched spike architecture |
| PE 00489015 | *Elymus dahuricus* Turcz. | Qinghai, China | PE | No | Partial | Weakly suggestive | Spike loosely arranged, with minor bending; spikelets not tightly packed; no lateral branchlets observed |
| PE00489006 | *Elymus dahuricus* Turcz. | Gansu,China | PE | No | Partial | Weakly suggestive | Spikelets loosely arranged, a few nodes exhibiting intermediate morphology between simple and compound spikes; lateral branchlets or compound spike structures absent. |
| PE00489004 | *Elymus dahuricus* Turcz. | Gansu, China | PE | No | None | None | Spike unbranched; no lateral branchlets or compound spike structures observed; typical single-type spike architecture |
| PE00488950 | *Elymus dahuricus* Turcz. | Shaanxi, China | PE | No | Partial | Weakly suggestive | Loosely arranged spikes; minor irregularities in spikelets; no lateral branchlets or compound spike framework observed |
| WUK0491433 | *Elymus dahuricus* Turcz. | Shaanxi, China | WUK | No | Low | No | Spike slender and strictly linear throughout; spikelets regularly sessile along the rachis without lateral elongation, clustered branch-like fascicles, or secondary branch formation. Overall architecture consistent with a typical simple spike of Elymus dahuricus. |
| HNWP 0212781 | *Elymus dahuricus* Turcz. | Qinghai, China | HNWP | No | Weakly suggestive | Weakly suggestive | Spikes generally linear and unbranched, but several spikelets are loosely spaced and slightly divergent from the main rachis axis, producing a weakly irregular spike outline. No lateral branchlets, secondary axes, or clustered branch systems observed. |
| HNWP 105101 | Elymus dahuricus Turcz. | Qinghai, China | HNWP | No | Weakly suggestive | Weakly suggestive | Spike generally linear and unbranched; several spikelets in the middle portion are slightly lax and somewhat displaced from the rachis axis, producing weak local irregularity in spike organization. No definite lateral branchlets, secondary spike axes, or compound branching structures observed. Leaf overlap partially obscures the upper-middle portion of the spike. |
| HNWP 130986 | Elymus dahuricus Turcz. | Qinghai, China | HNWP | No | Weakly suggestive | Weakly suggestive | Spike narrow and predominantly linear, but several spikelets are irregularly spaced and slightly displaced from the rachis axis, producing a somewhat flexuous spike outline. No lateral branchlets, secondary spike axes, or clustered branch systems observed. |
| HNWP 153281 | Elymus dahuricus Turcz. | Qinghai, China | HNWP | No | Low | No | Spike slender, compact, and uniformly linear; spikelets regularly arranged along the rachis without displacement, lax spacing, or local clustering. No lateral branchlets, secondary spike axes, or compound branching tendencies observed. Overall architecture consistent with a typical simple spike of Elymus dahuricus. |
| HNWP 185435 | Elymus dahuricus Turcz. | Qinghai, China | HNWP | No | Low | No | Spike compact and condensed with strongly appressed spikelets; the apparent fan-shaped outline is caused by long awns spreading outward rather than by branching of the spike axis. Spikelets remain directly attached to a single rachis, and no lateral branchlets, secondary spike axes, or clustered branch systems are observed. |
| HNWP 78647 | Elymus dahuricus Turcz. | Gansu, China | HNWP | No | Weakly suggestive | Weakly suggestive | Spike elongated and narrowly cylindrical with a slightly uneven outline; several spikelets are loosely arranged and somewhat divergent from the rachis, producing localized relaxation of the spike contour. Awns are relatively long and spreading, giving parts of the spike a weakly plumose appearance, but all spikelets remain attached directly to a single unbranched rachis without formation of lateral branch systems. |
| HNWP 98221 | Elymus dahuricus Turcz. | Qinghai, China | HNWP | No | Weakly suggestive | Weakly suggestive | Spike elongated and flexuous with uneven spacing of spikelets along the rachis; several spikelets are loosely arranged and slightly displaced outward from the main axis, creating localized widening and partial relaxation of the spike contour. Long spreading awns produce a somewhat plumose appearance in portions of the spike, but all spikelets remain sessile on a single continuous rachis without true lateral branch formation. |
| HNWP 97747 | *Elymus dahuricus* Turcz. | Qinghai, China | HNWP | No | No suggestive | No suggestive | Spike generally linear, slight irregularities; some spikelets show minor deviation toward intermediate traits, but no lateral branchlets observed. Two spikes are visible in the second image, one partially overlapped by leaf; careful observation shows linear form maintained. |
| HNWP 00000141 | *Elymus dahuricus* Turcz. | Qinghai, China | HNWP | No | Weakly suggestive | Weakly suggestive | Spike shows linear to slightly curved form; spikelets partially detached, giving impression of minor irregularities. The overall axis is preserved; no true lateral branches observed. Leaf arrangement and overlapping in the first image obscure portions of spikelets, requiring careful inspection to confirm spike linearity. |
| SZ 02030288 | *Elymus dahuricus* Turcz. | Sichuan, China | SZ | No | Weakly suggestive | Weakly suggestive | Spike mostly linear, slight curvature; some spikelets show minor deviation toward intermediate traits. Spike is fully visible in the second image; note some small flowers have detached. Overall linear architecture maintained, no lateral branchlets observed. |
| IFP 15855003x0030 | Elymus dahuricus Turcz. | Inner Mongolia, China | IFP | No | Weakly suggestive | Weakly suggestive | Spike mostly linear, some slight curvature in basal portion; spikelets aligned along central rachis, no lateral branches observed. Some leaves and stems partially obscure the spike, but the spike’s linearity is maintained. Minor irregularities in spikelet orientation, but no clear deviation toward lateral branching. |
| IFP 15855003x0027 | Elymus dahuricus Turcz. | Inner Mongolia, China | IFP | No | Weakly suggestive | Weakly suggestive | Spike linear, slightly irregular; spikelets generally aligned along the rachis; no lateral branchlets observed. The spike shows minor zigzag bending at the base, but the overall linear structure is maintained. |
| IFP 15855003x0030 | Elymus dahuricus Turcz. | Inner Mongolia, China | IFP | No | Weakly suggestive | Weakly suggestive | Spike generally linear, slight irregularities; spikelets show minor deviation toward intermediate traits, but no lateral branchlets observed. Spike apex slightly bent; spikelets mostly intact, some disarticulated. |
| N 019106432 | *Elymus dahuricus* Turcz. | China | N | No | Weakly suggestive | Weakly suggestive | Spike generally linear, slight irregularities; spikelets compact, no lateral branchlets observed. The single spike is well-preserved, showing typical *E. dahuricus* linear form, slightly curved at apex. |
| N 019106442 | *Elymus dahuricus* Turcz. | Qinghai, China | N | No | No suggestive | No suggestive | Spike generally linear, slight irregularities; spikelets mostly intact, minor bending observed; no lateral branchlets. Spike appears slightly compressed and flattened due to mounting; tip and basal part well preserved. |
| N 019106449 | *Elymus dahuricus* Turcz. | Inner Mongolia, China | N | No | Weakly suggestive | Weakly suggestive | Spike linear, well-formed; slight curvature at the base but no lateral branchlets observed. Spikelets generally aligned along the rachis, with minor deviations in orientation. The overall spike morphology is consistent with typical E. dahuricus, with no clear evidence of branching or transitional forms. |
| N 019106464 | Elymus dahuricus Turcz. | Sichuan, China | N | No | Weakly suggestive | Weakly suggestive | Spike linear, slightly curved, minor irregularities in spikelet alignment; no lateral branchlets observed. Two spikes visible, linear form maintained overall. |
| BNU 0031572 | *Elymus dahuricus* Turcz. ex Griseb. | Qinghai, China | BNU | No | Weakly suggestive | Weakly suggestive | Spike overall linear and erect, minor irregularities in spikelet arrangement; no lateral branchlets observed. Spike tip slightly curved but maintains linear form. |
| HNWP 003485 | Elymus dahuricus Turcz. | Qinghai, China | HNWP | No | Weakly suggestive | Weakly suggestive | Spike generally linear, minor irregularities visible; some spikelets slightly divergent but no clear lateral branchlets. Two spikes visible, overlapping in part with other stems; linear form maintained. |
| HNWP 003498 | *Elymus dahuricus* Turcz. | Qinghai, China | HNWP | No | Weakly suggestive | Weakly suggestive | Spike mostly linear; slight unevenness observed; some spikelets show minor deviation toward intermediate forms, but no true lateral branchlets are present. Two spikes are visible, one slightly overlapping leaf, careful observation shows linear form maintained. |
| HNWP 106474 | *Elymus dahuricus* Turcz. | Qinghai, China | HNWP | No | Weakly suggestive | Weakly suggestive | Slight deviation in spikelet orientation in middle spike. No lateral branchlets. Consistent with *E. dahuricus*. |
| HNWP 157474 | *Elymus dahuricus* Turcz. | Qinghai, China | HNWP | No | Weakly suggestive | Weakly suggestive | Spike linear form maintained; spikelets slightly appressed; no lateral branchlets. Some leaves overlap spike base. |
| HNWP 73398 | *Elymus dahuricus* Turcz. | Qinghai, China | HNWP | No | Weakly suggestive | Weakly suggestive | pike shows slight curvature and minor irregularities in spikelet alignment, but maintains a generally linear form. No lateral branches are visible. The image shows two spikes positioned close to each other, with slight overlapping of basal leaves; careful observation confirms each spike maintains linear architecture. |
| HNWP 73428 | *Elymus dahuricus* Turcz. | Qinghai, China | HNWP | No | Very weakly suggestive | Not clearly suggestive | The specimen shows several elongated spikes placed close together, with partial overlap among awns and spikelets. However, each visible spike appears to maintain a linear, unbranched rachis. No true lateral spike branch arising from the main rachis is visible. The apparent complexity is mainly caused by multiple spikes lying parallel or overlapping, plus dense awns and slightly irregular spikelet orientation. Therefore, this specimen should not be interpreted as having a branched spike. |
| HNWP 97633 | *Elymus dahuricus* Turcz. | Qinghai, China | HNWP | No | Not suggestive | Not suggestive | The visible spike is narrow, slightly curved or drooping, and densely awned, but it retains a single linear spike axis. No lateral branch arising from the main rachis can be observed. The apparent thickness and irregular outline are mainly caused by the dense awns, overlapping spikelets, and the pressed condition of the specimen. |
| HNWP 98186 | *Elymus dahuricus* Turcz. | Qinghai, China | HNWP | No | Not suggestive | Not suggestive | The visible spike is slender, linear, and slightly curved, with spikelets arranged along a single continuous rachis. No lateral spike branch arising from the main axis is visible. Some leaf blades and culms cross over the inflorescence, and there may be more than one separate spike on the sheet, but these are separate structures rather than branches of one spike. The spikelet arrangement is somewhat loose and slightly irregular, but this remains within ordinary unbranched *E. dahuricus* morphology. |
| HNWP 98219 | *Elymus dahuricus* Turcz. | Qinghai, China | HNWP | No | Weakly suggestive | Weakly suggestive | The spike appears compact and somewhat irregular. However, no clear lateral branch arising from a single main rachis can be confirmed. |
| HNWP 98236 | *Elymus dahuricus* subsp. *cylindricus* (Franch.) N. R. Cui | Qinghai, China | HNWP | No | Not suggestive | Not suggestive | The specimen shows a single, narrow, erect to slightly curved spike with a continuous linear rachis. Spikelets are appressed and arranged closely along the main axis, giving the spike a slender cylindrical appearance. No lateral spike branch or secondary axis can be observed. This specimen represents a clearly unbranched spike condition. |
| HNWP 98238 | *Elymus dahuricus* Turcz. | Qinghai, China | HNWP | No | Not suggestive | Not suggestive | The specimen shows a single elongated spike that is slightly curved, with spikelets arranged along one continuous rachis. The awns project outward and make the spike outline somewhat loose, but there is no visible lateral branch or secondary spike axis. The spike maintains a typical unbranched Elymus dahuricus architecture. The curvature and awn spreading should not be overinterpreted as transitional branched morphology. |
| HNWP 000000408 | *Elymus dahuricus* Turcz. | Qinghai, China | HNWP | No | Not suggestive | Not suggestive | The specimen shows a very slender, elongated, slightly curved to drooping spike. The spikelets are arranged singly along one continuous rachis and appear closely appressed to the axis. No lateral spike branch or secondary rachis is visible. |
| N 019106472 | *Elymus dahuricus* Turcz. | Qinghai, China | N | No | Not suggestive | Not suggestive | The two visible spikes are close to each other but remain independent, linear, unbranched spike structures. Each spike has a single continuous rachis, with spikelets arranged along the main axis. No lateral branch arising from a main spike axis is visible. The slight looseness and awn spreading may create minor visual irregularity, but this should not be interpreted as branched or transitional inflorescence morphology. |
| PE 00489014 | *Elymus dahuricus* Turcz. | Qinghai, China | PE | No | Not suggestive | Not suggestive | The visible spikes remain separate linear structures, each with a single continuous rachis. No lateral spike branch arising from a main rachis can be confirmed. This specimen should therefore be treated as an unbranched *Elymus dahuricus* form. |
| HNWP003484 | *Elymus dahuricus* Turcz. | Qinghai, China | HNWP | No | Weakly suggestive | Weakly suggestive | Each visible spike maintains a linear, unbranched architecture, with spikelets arranged along one continuous axis. |
| HNWP 101188 | *Elymus dahuricus* Turcz. | Qinghai, China | HNWP | No | Not suggestive | Not suggestive | The specimen shows a narrow, elongated, drooping spike with spikelets arranged along a single continuous rachis. The spike is slightly dense and awned, but no lateral branch or secondary spike axis is visible. |
| HNWP 104931 | *Elymus dahuricus* Turcz. | Qinghai, China | HNWP | No | Weakly suggestive | Weakly suggestive | The visible structures of the spikes are better interpreted as multiple independent linear spikes lying parallel and overlapping, rather than lateral branches arising from one main rachis. No clear point of branching from a single spike axis can be confirmed. |
| HNWP 11749 | *Elymus dahuricus* Turcz. | Qinghai, China | HNWP | No | Not suggestive | Not suggestive | The specimen shows a single terminal spike on a long, strongly curved culm. The spike is narrow, drooping, and slightly curved, with spikelets arranged along one continuous rachis. No lateral branch or secondary spike axis is visible. The inflorescence is therefore clearly unbranched and does not show transitional morphology toward the branched-spike condition of *E. chaobotryus*. |
| HNWP 120457 | *Elymus dahuricus* subsp. *cylindricus* (Franch.) N. R. Cui | Qinghai, China | HNWP | No | Not suggestive | Not suggestive | The specimen shows a narrow, drooping spike with spikelets arranged along a single continuous rachis. In the enlarged image, the spike is partly overlapped by long culms or leaves, but no lateral spike branch or secondary rachis can be observed. The spike remains consistent with an unbranched *Elymus dahuricus* subsp. *cylindricus* morphology. |
| HNWP 120752 | *Elymus dahuricus* Turcz. | Qinghai, China | HNWP | No | Not suggestive | Not suggestive | The specimen shows elongated spikes with relatively large, appressed spikelets arranged along a single continuous rachis. The spike is slightly curved and the spikelets are somewhat spreading, but no lateral branch or secondary spike axis is visible. The specimen therefore represents an unbranched *Elymus dahuricus* spike condition. |
| HNWP 12261 | *Elymus dahuricus* Turcz. | Qinghai, China | HNWP | No | Not suggestive | Not suggestive | The two spikes are separate terminal spikes, each with its own continuous rachis. No lateral branch arising from a single main spike axis can be confirmed. |
| HNWP 130523 | *Elymus dahuricus* Turcz. | Qinghai, China | HNWP | No | Not suggestive | Not suggestive | The specimen shows a single elongated spike that is slightly curved, with spikelets arranged along one continuous rachis. The spike is somewhat dense and awned, but no lateral branch or secondary spike axis is visible. |
| HNWP 130658 | *Elymus dahuricus* Turcz. | Qinghai, China | HNWP | No | Not suggestive | Not suggestive | The visible spike is narrow, slightly curved, and arranged along a single continuous rachis. The spike is partly overlapped by culms or leaves, but no lateral branch or secondary spike axis can be confirmed. |
| HNWP 134220 | *Elymus dahuricus* Turcz. | Qinghai, China | HNWP | No | Not suggestive | Not suggestive | The specimen shows a single narrow, drooping spike with spikelets arranged along one continuous rachis. The spike is linear and slightly dense, with awns projecting outward, but no lateral branch or secondary spike axis is visible. |
| HNWP 152550 | *Elymus dahuricus* Turcz. | Qinghai, China | HNWP | No | Not suggestive | Not suggestive | The specimen shows a single slender, slightly curved spike with spikelets arranged along one continuous rachis. The spike is narrow and somewhat loose, with awns projecting outward, but no lateral branch or secondary spike axis is visible. |
| HNWP 153000 | *Elymus dahuricus* Turcz. | Qinghai, China | HNWP | No | Not suggestive | Not suggestive | The specimen shows at least two visible spikes on the sheet, but they appear to be separate terminal spikes rather than parts of a branched inflorescence. The spike is narrow, slightly curved, and densely awned, with spikelets arranged along a single continuous rachis. No lateral branch or secondary spike axis can be observed. |
| HNWP 153003 | *Elymus dahuricus* Turcz. | Qinghai, China | HNWP | No | Not suggestive | Not suggestive | The specimen shows more than one spike on the sheet, but the visible spikes appear to be separate terminal spikes rather than components of a branched inflorescence. The spike is slender, slightly curved, and awned, with spikelets arranged along a single continuous rachis. No lateral branch or secondary spike axis is visible. |
| HNWP 154704 | *Elymus dahuricus* Turcz. | Qinghai, China | HNWP | No | Not suggestive | Not suggestive | No clear branching point from a single main spike axis can be confirmed. |
| HNWP 185251 | *Elymus dahuricus* Turcz. | Qinghai, China | HNWP | No | Weakly suggestive | Weakly suggestive | The specimen indicates that the visible spikes are borne on separate culms or separate axes, rather than lateral branches arising from a single main spike rachis. Each spike appears to maintain its own continuous linear axis. No clear branching point from one spike rachis can be confirmed. |
| HNWP 22814 | *Elymus dahuricus* Turcz. | Qinghai, China | HNWP | No | Very weakly suggestive | Not clearly suggestive | The spike is elongated, slightly curved, and awned, with spikelets arranged along a single continuous rachis. No clear lateral branch arising from the main spike axis is visible. |
| HNWP 73288 | *Elymus dahuricus* Turcz. | Qinghai, China | HNWP | No | Weakly suggestive | Weakly suggestive | The two spike-like structures are better interpreted as separate spikes that overlap or cross in the mounted specimen. Each visible spike appears to retain a linear, unbranched rachis. No clear branching point from a single main rachis can be confirmed. |
| HNWP 73293 | *Elymus dahuricus* subsp. *cylindricus* (Franch.) N. R. Cui | Qinghai, China | HNWP | No | Not suggestive | Not suggestive | The specimen shows two visible terminal spikes on separate culms. The enlarged spike is slender, slightly curved, and loosely awned, with spikelets arranged along a single continuous rachis. No lateral branch or secondary spike axis is visible. |
| HNWP 76930 | *Elymus dahuricus* Turcz. | Qinghai, China | HNWP | No | Weakly suggestive | Weakly suggestive | No clear lateral branch arising from a single main rachis can be confirmed. The visible structures are better interpreted as separate spikes or spike portions overlapping in the mounted specimen. Each spike retains a linear, unbranched architecture. |
| HNWP 98229 | *Elymus dahuricus* Turcz. | Qinghai, China | HNWP | No | Weakly suggestive | Weakly suggestive | In the specimen, the left and right spikes are visually close and partly aligned, which gives a weak superficial impression of a branched or compound inflorescence. However, no clear lateral branch arising from a single main rachis can be confirmed. Each visible spike appears to maintain its own linear, unbranched axis. |
| HNWP 00002442 | *Elymus dahuricus* Turcz. | Qinghai, China | HNWP | No | Not suggestive | Not suggestive | The specimen shows elongated, slightly curved spikes with spikelets arranged along a single continuous rachis. In the enlarged image, the main visible spike is linear and unbranched; the apparent unevenness is caused by spikelet orientation, awns, and nearby overlapping culms or leaves. No lateral spike branch or secondary rachis can be observed. |
| N 019106508 | *Elymus dahuricus* var. *sinensis* | Qinghai, China | N | No | Not suggestive | Not suggestive | The specimen shows a slender, linear spike with spikelets arranged along a single continuous rachis. In the enlarged image, the spike is partly crossed by culms or leaves, but no lateral spike branch or secondary rachis is visible. The apparent crossing structures are separate vegetative axes, not branches of the inflorescence. |
| N 019106509 | *Elymus dahuricus* Turcz. | Qinghai, China | N | No | Not suggestive | Not suggestive | The specimen shows a single curved, somewhat loose spike with spikelets arranged along one continuous rachis. In the enlarged image, the spikelets are slightly spreading and the rachis is curved, creating some visual irregularity, but no lateral branch or secondary spike axis is visible. |
| BJFC00004727 | *Elymus dahuricus* var. *cylindricus* Franch. | Qinghai, China | BJFC | No | Not suggestive | Not suggestive | The specimen shows a slender, pale, slightly curved spike with spikelets arranged along a single continuous rachis. In the enlarged image, the spike is partly crossed near its base by a culm or leaf, creating a minor visual intersection, but this structure is not part of the spike axis. No lateral spike branch or secondary rachis can be confirmed. |
| PE 00489129 | *Elymus dahuricus* Turcz. | Qinghai, China | PE | No | Not suggestive | Not suggestive | The specimen shows several elongated spikes positioned close together near the upper-right part of the sheet. In the enlarged image, one curved spike lies close to a straighter adjacent spike, producing a weak superficial impression of a branched or compound inflorescence. However, no clear lateral branch arising from a single main rachis can be confirmed. The visible spikes appear to be separate terminal spikes on separate culms rather than branches of one spike axis. |
| PE 00489016 | *Elymus dahuricus* Turcz. | Qinghai, China | PE | No | Weakly suggestive | Weakly suggestive | The specimen shows several slender, curved to drooping spikes mounted close together. In the enlarged image, some spikes are partly overlapping or positioned at different angles, which creates a weak superficial impression of a compound or branched inflorescence. However, no clear lateral branch arising from a single main rachis can be confirmed. Each visible spike appears to retain its own linear axis, and the apparent complexity is more likely caused by multiple independent spikes, loose spikelet arrangement, and specimen pressing. |
| HNWP 003497 | *Elymus dahuricus* Turcz. | Qinghai, China | HNWP | No | Not suggestive | Not suggestive | The specimen shows a single narrow, drooping spike with spikelets arranged along one continuous rachis. In the enlarged image, the spike is linear and somewhat compact, with awns projecting outward, but no lateral branch or secondary spike axis is visible. The short exposed segment above the fixing strip appears to be part of the same terminal axis or mounting artifact, not a true branch. |
| HNWP 003512 | *Elymus dahuricus* subsp. *cylindricus* (Franch.) N. R. Cui | Qinghai, China | HNWP | No | Not suggestive | Not suggestive | The specimen shows a single slender, erect to slightly inclined spike with spikelets arranged along one continuous rachis. In the enlarged image, the spike is narrow and compact, with appressed spikelets and only limited awn spreading. No lateral spike branch or secondary rachis is visible. |
| HNWP 0212797 | *Elymus dahuricus* Turcz. | Qinghai, China | HNWP | No | Very weakly suggestive | Very weakly suggestive | The specimen shows several long culms and more than one spike-bearing axis on the sheet, with some culms and leaves strongly curved or overlapping. In the enlarged image, the visible spike is slender, slightly curved, and awned, with spikelets arranged along a single continuous rachis. No lateral spike branch or secondary spike axis can be confirmed. The weak visual complexity comes from curved culms, separate spikes, and mounting overlap, not from true branching of one inflorescence. |
| HNWP 105667 | *Elymus dahuricus* subsp. *cylindricus* (Franch.) N. R. Cui | Qinghai, China | HNWP | No | Weakly suggestive | Not clearly suggestive | The specimen shows several narrow, drooping spikes positioned close to each other in the upper part of the sheet. This clustered arrangement can create a weak superficial impression of a compound or branched inflorescence. The spikes appear to be borne on separate culms or separate terminal axes, not as lateral branches arising from a single main rachis. Each visible spike maintains a linear, unbranched structure. No clear branching point from one spike axis can be confirmed. |
| HNWP 120738 | *Elymus dahuricus* Turcz. | Qinghai, China | HNWP | No | Weakly suggestive | Weakly suggestive | The specimen shows two to three long, drooping, densely awned spike-like structures positioned very close together. No clear lateral branch arising from a single main rachis can be confirmed. The visible spikes are better interpreted as separate terminal spikes or overlapping spike portions, each maintaining its own linear axis. |
| HNWP 190407 | *Elymus dahuricus* Turcz. | Qinghai, China | HNWP | No | Weakly suggestive | Weakly suggestive | The specimen shows several spike-bearing culms, with some spikes positioned close together and partly overlapping. In the enlarged image, two elongated spike-like structures are adjacent and nearly parallel. No clear lateral branch arising from a single main rachis can be confirmed. The visible structures are better interpreted as separate spikes or overlapping spike-bearing axes. Each spike appears to retain a linear, unbranched rachis. |
| HNWP 73424 | *Elymus dahuricus* Turcz. | Qinghai, China | HNWP | No | Weakly suggestive | Weakly suggestive | The specimen shows two elongated spikes positioned close to each other in the upper part of the sheet. In the enlarged image, both spikes are linear, slightly curved, and awned, each with spikelets arranged along a single continuous rachis. No lateral branch arising from one main rachis can be confirmed. The two spikes are separate terminal spikes on separate axes. |
| HNWP 98232 | *Elymus dahuricus* Turcz. | Qinghai, China | HNWP | No | Very weakly suggestive | Very weakly suggestive | The specimen shows two elongated spikes positioned near each other in the upper part of the sheet. In the enlarged image, both spikes are linear and awned, each maintaining its own continuous rachis. Their close placement may create a very weak superficial impression of a compound or branched inflorescence, but no lateral branch arising from a single main rachis can be confirmed. The central leaf or culm crossing between the spikes is a separate vegetative structure and should not be interpreted as part of a branched spike. |
| HNWP 13179 | *Elymus dahuricus* Turcz. | Qinghai, China | HNWP | No | Not suggestive | Not supported | The specimen shows a single narrow, slightly curved to drooping spike with spikelets arranged along one continuous rachis. In the enlarged image, the spike is partly covered by a fixing strip, and a brown culm or leaf crosses behind it, but no lateral spike branch or secondary rachis is visible. |
| HNWP 13840 | *Elymus dahuricus* Turcz. | Qinghai, China | HNWP | No | Not suggestive | Not supported | The specimen shows a single narrow, drooping spike with spikelets arranged along one continuous rachis. In the enlarged image, the spike is slightly curved and somewhat loose, with awns projecting outward, but no lateral branch or secondary spike axis is visible. |
| HNWP 00000643 | *Elymus dahuricus* Turcz. | Qinghai, China | HNWP | No | Not suggestive | Not supported | The specimen shows a single visible, slender, drooping to slightly curved spike. In the enlarged image, spikelets are arranged along one continuous rachis, with no lateral spike branch or secondary rachis visible. |
| HNWP00012580 | *Elymus dahuricus* var. *cylindricus* Franch. | Qinghai, China | HNWP | No | Not suggestive | Not supported | The specimen shows several culms and more than one spike-bearing axis, but the visible spikes appear to be separate terminal spikes rather than branches of a single inflorescence. In the enlarged image, the spike is narrow, linear to slightly curved, and relatively compact, with spikelets arranged along one continuous rachis. No lateral spike branch or secondary rachis is visible. |
| N 019106439 | *Elymus dahuricus* Turcz. | Qinghai, China | N | No | Not suggestive | Not supported | The specimen shows more than one spike-bearing culm on the sheet, but the enlarged inflorescence maintains a single linear spike axis. Spikelets are arranged along one continuous rachis, with awns spreading outward. No secondary rachis or clear branching point from the main spike axis can be confirmed. |
| BJFC00004729 | *Elymus dahuricus* var. *cylindricus* Franch. | Qinghai, China | BJFC | No | Not suggestive | Not supported | The specimen shows a single very slender, curved to drooping spike at the end of a long culm. In the enlarged image, the spike appears narrow and somewhat loose, with spikelets arranged along one continuous rachis. No lateral spike branch or secondary rachis is visible. |
| SAUT 201400534 | *Elymus dahuricus* Turcz. ex Griseb. | Qinghai, China | SAUT | No | Not suggestive | Not supported | The specimen shows several elongated spikes positioned close to culms and leaves. In the enlarged image, the right spike has a curved main axis, and a short spike-like structure near its lower portion creates a weak superficial impression of branching. No clear lateral branch arising directly from the main spike rachis can be confirmed. |
| KUN 1481019 | *Elymus dahuricus* var. *cylindricus* Franch. | Qinghai, China | KUN | No | Not suggestive | Not supported | The specimen shows a slender, drooping spike with nearby spike-like material closely appressed in the enlarged image. No clear lateral rachis arising from a single main spike axis can be confirmed. |
| KUN 337236 | *Elymus dahuricus* Turcz. | Qinghai, China | KUN | No | Not suggestive | Not supported | The specimen shows a single narrow, erect spike with spikelets arranged along one continuous rachis. In the enlarged image, the spike is compact and linear, with no visible lateral spike branch or secondary rachis. |
| HNWP 0212782 | *Elymus dahuricus* Turcz. | Qinghai, China | HNWP | No | Not suggestive | Not supported | The specimen shows several elongated, awned spikes positioned close together near the upper part of the sheet. No clear lateral branch arising from a single main rachis can be confirmed. The visible spikes are separate terminal spikes on different culms or overlapping spike-bearing axes. |
| HNWP 105726 | *Elymus dahuricus* subsp. *cylindricus* (Franch.) N. R. Cui | Qinghai, China | HNWP | No | Not suggestive | Not supported | The specimen shows several elongated, awned spikes positioned close together. In the enlarged image, a long curved spike lies near a shorter spike and adjacent culm-like axes, no clear lateral rachis arising from a single main spike axis can be confirmed. |
| HNWP 130445 | *Elymus dahuricus* Turcz. | Qinghai, China | HNWP | No | Not suggestive | Not supported | The specimen shows a relatively dense, elongated spike with several culms and leaves crossing around the inflorescence. No clear lateral rachis arising directly from the main spike axis can be confirmed. |
| HNWP 13148 | *Elymus dahuricus* Turcz. | Qinghai, China | HNWP | No | Not suggestive | Not supported | The specimen shows a single visible, narrow spike positioned on the right side of the sheet. In the enlarged image, the spike is slightly curved and awned, with spikelets arranged along one continuous rachis. No lateral spike branch, secondary rachis, or branching point can be confirmed. |
| HNWP 152548 | *Elymus dahuricus* Turcz. | Qinghai, China | HNWP | No | Not suggestive | Not supported | The specimen shows more than one spike-bearing culm on the sheet, but the enlarged spike is clearly a single, narrow, linear to slightly curved inflorescence. Spikelets are arranged along one continuous rachis, and no lateral spike branch or secondary rachis is visible. |
| HNWP 153171 | *Elymus dahuricus* Turcz. | Qinghai, China | HNWP | No | Not suggestive | Not supported | The specimen shows several spike-bearing culms, but the visible spikes are separate terminal spikes rather than branches of one inflorescence. Each spike maintains a single continuous rachis with spikelets arranged linearly. No lateral spike branch, secondary rachis, or definite branching point can be confirmed. |
| HNWP 154706 | *Elymus dahuricus* Turcz. | Qinghai, China | HNWP | No | Not suggestive | Not supported | The specimen shows two visible spike-like inflorescences positioned close together among crossing culms and leaves. Each visible spike appears to have its own continuous rachis, and no lateral spike branch arising from a single main rachis can be confirmed. |
| HNWP 157022 | *Elymus dahuricus* Turcz. | Qinghai, China | HNWP | No | Not suggestive | Not supported | The specimen shows more than one spike-bearing culm on the sheet, but the visible inflorescences are separate terminal spikes rather than branches of one spike. In the enlarged image, the examined spike is narrow, drooping, and slightly curved, with spikelets arranged along a single continuous rachis. |
| HNWP 158012 | *Elymus dahuricus* Turcz. | Qinghai, China | HNWP | No | Not suggestive | Not supported | The specimen shows a single narrow, drooping terminal spike. In the enlarged image, the spikelets are arranged along one continuous rachis, with no visible lateral rachis, secondary spike, or true branching node. |
| HNWP 178324 | *Elymus dahuricus* Turcz. | Qinghai, China | HNWP | No | Not suggestive | Not supported | The specimen bears two visible spike-like inflorescences placed close to each other near the upper part of the sheet. However, the enlarged image shows that these are separate simple spikes on separate culm/peduncle portions, not lateral branches arising from nodes of a single main rachis. Each spike is slender, drooping to slightly arcuate, with spikelets arranged along a continuous unbranched rachis. |
| HNWP 73294 | *Elymus dahuricus* Turcz. | Qinghai, China | HNWP | No | Not suggestive | Not supported | The specimen shows two visible spike-like inflorescences, but they are borne on separate culms or separate peduncle portions rather than arising as lateral branches from a common main rachis. The enlarged inflorescence is a simple, slender, slightly curved spike with spikelets arranged along a single continuous axis. No secondary spike-bearing branch, nodal rachis branch, or repeated branched spike architecture is visible. |
| HNWP 98226 | *Elymus dahuricus* subsp. *cylindricus* (Franch.) N. R. Cui | Qinghai, China | HNWP | No | Not suggestive | Not supported | The specimen shows several slender, drooping spike-like inflorescences, but the visible structures represent separate simple spikes rather than a branched spike system.No clearly observable common main rachis giving rise to lateral spike-bearing branches. The spikelets are arranged along single continuous axes. |
| HNWP 98227 | *Elymus dahuricus* Turcz. | Qinghai, China | HNWP | No | Not suggestive | Not supported | The specimen shows more than one slender, lax, drooping spike, but the visible inflorescences appear to be separate simple spikes rather than branches of a single spike system. No clear common main rachis with lateral spike-bearing branches is visible. |
| HNWP 0233290 | *Elymus dahuricus* Turcz. | Qinghai, China | HNWP | No | Not suggestive | Not supported | The specimen shows two visible spike-like inflorescences near the left side of the sheet. Both appear to be simple spikes borne on separate culms or peduncles. There is no evidence of a shared main rachis producing lateral spike-bearing branches. |
| HNWP 26230 | *Elymus dahuricus* Turcz. | Qinghai, China | HNWP | No | Not suggestive | Not supported | The specimen shows a single clearly visible spike-like inflorescence on the left side of the sheet. The spike is elongated, slightly curved, and moderately lax, with awned spikelets giving a somewhat feathery outline. No node on the rachis shows a distinct side branch comparable to the branched-spike architecture of *E. chaobotryus*. |
| N 019106436 | *Elymus dahuricus* Turcz. | Qinghai, China | N | No | Not suggestive | Not supported | The specimen shows two visible spike-like inflorescences near the upper part of the sheet. They are slender, somewhat lax, and arranged close to one another. Each visible spike is borne on its own slender peduncle or culm segment. There is no clear shared main rachis from which a lateral spike-bearing branch arises. |
| N 019106440 | *Elymus dahuricus* Turcz. | Qinghai, China | N | No | Weak superficial resemblance only | Not supported | The specimen shows a long, pendent to strongly curved spike, with a loose and somewhat feathery outline caused by awned spikelets. In the enlarged image, the inflorescence is organized along a single continuous rachis. The spikelets are inserted sequentially along that axis; no node shows a distinct lateral spike-bearing branch. |
| N 019106460 | *Elymus dahuricus* Turcz. | Qinghai, China | N | No | Weak superficial resemblance only | Not supported | he specimen shows a slender, pendent to curved spike with long awns, giving the inflorescence a loose and somewhat feathery appearance. In the enlarged image, the spikelets are arranged along a single continuous rachis. No node shows a distinct side branch bearing secondary spikelets or mini-spike-like units. |
| BNU 0031602 | *Elymus dahuricus* Turcz. ex Griseb. | Qinghai, China | BNU | No | Weak superficial resemblance only | Not supported | The specimen shows a single slender, curved to slightly flexuous spike with long awns, producing a loose and somewhat feathery outline. The spikelets remain arranged along one continuous rachis. There is no clear lateral spike-bearing branch, no repeated secondary mini-spike-like units, and no basal elongation of rachillae comparable to the branched-spike architecture of *E. chaobotryus*. The apparent angular change is best interpreted as curvature or mounting deformation of a simple spike. |
| BJFC00004728 | *Elymus dahuricus* var. *cylindricus* Franch. | Qinghai, China | BJFC | No | Weak superficial resemblance only | Not supported | The specimen shows a slender culm with a terminal, short to moderately elongated, curved spike. The enlarged image shows a compact awned spike with spikelets arranged along a single continuous rachis. The spike outline is slightly curved and feathery because of the long awns, but there is no clear lateral spike-bearing branch, no basal branch cluster, and no secondary mini-spike-like unit. |
| PE 00489019 | *Elymus dahuricus* Turcz. | Qinghai, China | PE | No | Weak superficial resemblance only | Not supported | The specimen shows a long, curved to pendulous spike with relatively dense spikelets and long awns, giving the inflorescence a loose, feathery outline. In the enlarged image, the spikelets are still arranged along a single continuous rachis. No node shows a secondary rachis, mini-spike-like branch, or basal branching unit comparable to the branched-spike condition of *E. chaobotryus*. |
| PE 00489013 | *Elymus dahuricus* Turcz. | Qinghai, China | PE | No | Weak superficial resemblance only | Not supported | The specimen contains several culms with separate, curved, awned spikes. In the enlarged image, the inflorescences are close to each other and partly overlapping. Each visible spike is borne on its own peduncle or culm, and the spikelets appear arranged along individual continuous rachises. |
| KUN 1446826 | *Elymus dahuricus* Turcz. ex Griseb. | Qinghai, China | KUN | No | Weak superficial resemblance only | Not supported | The specimen shows two visible fertile culms with terminal, erect to slightly curved, relatively dense awned spikes. The enlarged image is important: the right spike is clearly a single continuous spike, with spikelets arranged along one rachis. The left spike has a small overlapping structure near its lower part, but this does not resolve into a true lateral spike-bearing branch; it is more consistent with overlap, a damaged/pressed fragment, or adjacent vegetative material. There is no repeated secondary mini-spike-like unit, no clear branch axis arising from a rachis node, and no basal cluster of elongated rachillae comparable to the compound-spike condition of *E. chaobotryus*. |
| HNWP 0212775 | *Elymus dahuricus* Turcz. | Qinghai, China | HNWP | No | Weak superficial resemblance only | Not supported | The specimen shows several fertile culms with terminal, relatively dense, awned spikes. In the enlarged image, the three visible spikes are separate inflorescences, each apparently borne on its own peduncle or culm. The left spike is curved, while the middle and right spikes are more erect and compact. No structural evidence is visible for a main rachis producing lateral spike-bearing branches, and no secondary mini-spike-like units arise from rachis nodes. |
| HNWP 0212794 | *Elymus dahuricus* Turcz. | Qinghai, China | HNWP | No | Weak superficial resemblance only | Not supported | The specimen shows a single visible fertile culm with a terminal, curved to pendulous, awned spike. In the enlarged image, the spikelets are arranged along one continuous rachis. The slight change in direction near the upper part and the compressed appearance around the paper strip do not indicate a true branch; they are more consistent with bending, pressing, and partial concealment of the same spike. No lateral spike-bearing branch arises from a rachis node, and no secondary mini-spike-like unit is visible. |
| HNWP 11774 | *Elymus dahuricus* Turcz. | Qinghai, China | HNWP | No | Weak superficial resemblance only | Not supported | The specimen shows a single fertile culm with a terminal, curved to slightly pendulous, awned spike. In the enlarged image, the spikelets appear arranged along one continuous rachis. The spike is somewhat lax and feathery because of the long awns, but there is no visible lateral spike-bearing branch arising from the rachis. |
| HNWP 130657 | *Elymus dahuricus* Turcz. | Qinghai, China | HNWP | No | Weak superficial resemblance only | Not supported | The specimen shows several slender culms bearing terminal, narrow, awned spikes. No main rachis is seen producing lateral spike-bearing branches, and no secondary mini-spike-like units arise from rachis nodes. |
| HNWP 157925 | *Elymus dahuricus* Turcz. | Qinghai, China | HNWP | No | Weak superficial resemblance only | Not supported | The specimen shows several slender culms with terminal, narrow, awned spikes. In the enlarged image, the main visible spike is curved to pendulous and somewhat lax. The spikelets of the main inflorescence remain arranged along a single continuous rachis. No main rachis producing lateral spike-bearing branches is visible, and no secondary mini-spike-like units arise from rachis nodes. |
| HNWP 164052 | *Elymus dahuricus* subsp. *cylindricus* (Franch.) N. R. Cui | Qinghai, China | HNWP | No | Weak superficial resemblance only | Not supported | The specimen shows multiple culms with terminal, narrow, awned spikes. In the enlarged image, two spike-like units are close together and partly gathered near the same mounting strip, which may superficially suggest a forked or clustered condition. No main rachis gives rise to lateral spike-bearing branches, and no secondary mini-spike-like units are present at rachis nodes. The two visible spikes should therefore be interpreted as separate simple terminal spikes from different culms or peduncles. |
| HNWP 98195 | *Elymus dahuricus* subsp. *cylindricus* (Franch.) N. R. Cui | Qinghai, China | HNWP | No | Weak superficial resemblance only | Not supported | The specimen consists of several culms bearing terminal, narrow, awned spikes. The enlarged inflorescence shows a single continuous spike with regularly arranged spikelets attached directly to the rachis. No branching pattern is evident. The inflorescence is therefore interpreted as a typical simple spike rather than a compound or branched spike. |
| HNWP 98228 | *Elymus dahuricus* Turcz. | Qinghai, China | HNWP | No | Weak superficial resemblance only | Not supported | The specimen bears a single terminal, dense, awned spike. The enlarged image shows a continuous, unbranched rachis with spikelets inserted directly along the main axis. No lateral spike-bearing branches are visible, and no secondary mini-spike-like units arise from rachis nodes. |
| HNWP 98234 | *Elymus dahuricus* Turcz. | Qinghai, China | HNWP | No | Weak superficial resemblance only | Not supported | The specimen bears two closely adjacent, awned spike-like structures. No main rachis is visible producing lateral spike-bearing branches, and no secondary mini-spike-like units arise from rachis nodes. Each spike maintains its own independent axis throughout its length. |
| HNWP 00000140 | *lymus dahuricus* Turcz. | Qinghai, China | HNWP | No | Very low | Not supported | The specimen consists of a single tufted individual bearing one terminal inflorescence. The enlarged image shows a compact, strongly nodding spike with long awns, which may appear irregular because several spikelets are compressed and partially overlapping after drying and mounting. The inflorescence remains a single unbranched spike. All spikelets are attached directly along one central axis. |
| HNWP 00000755 | *Elymus dahuricus* Turcz. | Qinghai, China | HNWP | No | Very low | Not supported | The specimen consists of a solitary culm bearing a single terminal spike. The inflorescence is clearly a simple spike. All visible spikelets are inserted directly along one continuous central rachis. No lateral spike-bearing branches, branch initiation points, or secondary mini-spike-like units are present. |
| N 019106495 | *Elymus dahuricus* subsp. *cylindricus* (Franch.) N. R. Cui | Qinghai, China | N | No | Very low | Not supported | The specimen bears a single elongated terminal spike that is strongly curved and partly obscured by leaves and mounting materials. The inflorescence consists of one continuous rachis with spikelets attached directly along its axis. No lateral spike-bearing branches, branch insertion points, or secondary mini-spike-like units are visible. |
| N 019106444 | *Elymus dahuricus* Turcz. | Qinghai, China | N | No | Very low | Not supported | This specimen contains several detached culms and inflorescences mounted on the same sheet. The visible structures represent separate simple spikes belonging to different culms rather than branches arising from a common rachis.  Each spike is borne at the apex of an individual culm or peduncle, and no specimen shows a main rachis producing lateral spike-bearing branches. Likewise, no secondary mini-spike-like units are present. |
| N 019106462 | *Elymus dahuricus* Turcz. | Qinghai, China | N | No | None to very low | Not supported | This specimen includes several culms mounted together, with multiple spikes concentrated in the lower-right portion of the sheet. The spikes belong to separate culms and are merely brought together by specimen preparation and mounting. No common central rachis is visible, and there is no evidence that any spike arises laterally from another spike-bearing axis. Likewise, no secondary mini-spike-like units are present. |
| BNU 0038769 | *Elymus dahuricus* Turcz. | Qinghai, China | BNU | No | None to very low | Not supported | The specimen consists of a single culm bearing one terminal spike. The spike is narrow, compact, and unbranched throughout its length. A continuous central rachis with spikelets attached directly along the axis. No lateral spike-bearing branches, secondary spike units, or branching points are visible. The entire inflorescence conforms to a simple spike architecture typical of ordinary *Elymus dahuricus*. |
| PE 01608144 | *Elymus dahuricus* Turcz. | Qinghai, China | PE | No | None to very low | Not supported | The specimen consists of several culms, but only one clearly preserved inflorescence is visible. The spike is long, narrow, and strongly drooping, with spikelets inserted directly along a single central rachis. A continuous unbranched spike axis extending from the base to the apex. No lateral spike-bearing branches, secondary spike units, or branching nodes are visible. |
| PE 00489012 | *Elymus dahuricus* Turcz. | Qinghai, China | PE | No | None to very low | Not supported | The specimen contains several culms with two visible inflorescences. Both inflorescences are borne on separate culms and terminate independently. The enlarged image shows elongated, somewhat lax spikes with spikelets attached directly to a single rachis.  Although the lower spike is strongly curved and partially folded, the inflorescence remains a continuous unbranched axis. No branching node, lateral spike-bearing branch, or secondary spike unit is visible. |
| HNWP 105304 | *Elymus dahuricus* Turcz. | Qinghai, China | HNWP | No | None to very low | Not supported | The specimen consists of two fertile culms, each terminating in a single dense, cylindrical to narrowly lanceolate spike. The spikes are relatively compact and straight, with spikelets inserted directly along a single rachis. No evidence of a primary rachis producing lateral spike-bearing branches is visible.  The inflorescences represent ordinary simple terminal spikes. No compound architecture, branching node, or secondary spike system can be detected. |
| HNWP 105702 | *Elymus dahuricus* subsp. *cylindricus* (Franch.) N. R. Cui | Qinghai, China | HNWP | No | Weak superficial resemblance only | Not supported | The specimen consists of several slender culms, each terminating in a single narrow spike. All visible spikes appear to arise independently from separate culms. No primary rachis bearing lateral spike-producing branches can be identified. Each inflorescence is a simple terminal spike with spikelets attached directly along a single axis. No structural evidence of branched-spike architecture is visible. |
| HNWP 120427 | *Elymus dahuricus* Turcz. | Qinghai, China | HNWP | No | Weak superficial resemblance only | Not supported | The spikelets are inserted directly along one continuous rachis. No primary rachis giving rise to lateral spike-bearing branches can be identified. The apparent side structure visible near the upper portion of the spike is a leaf or vegetative organ crossing the inflorescence rather than a true branch. |
| HNWP 17600 | *Elymus dahuricus* Turcz. | Qinghai, China | HNWP | No | None to very low | Not supported | The specimen consists of several densely tufted culms, each terminating in a separate spike. Each spike is borne on its own culm, and no common primary rachis connecting the spikes can be observed. The spikes are independent reproductive units rather than lateral branches of a compound inflorescence. No branching node, secondary spike axis, or spike-bearing lateral branch is visible. |
| HNWP 33708 | *Elymus dahuricus* Turcz. | Qinghai, China | HNWP | No | None to very low | Not supported | The specimen consists of a single fertile culm bearing one terminal spike. The spike is narrow, cylindrical, and slightly tapering toward the apex. No branching structures are visible. The inflorescence terminates directly at the culm apex and there is no evidence of lateral spike-bearing branches, secondary axes, or compound architecture. |
| HNWP 64315 | *Elymus dahuricus* subsp. *cylindricus* (Franch.) N. R. Cui | Qinghai, China | HNWP | No | None | Not supported | The specimen is represented by a single culm bearing one terminal spike. The spike is narrow, elongate, and slightly flexuous, with spikelets inserted directly along the main rachis. No lateral spike-bearing branches, secondary rachises, or compound inflorescence structures are visible. The inflorescence remains a simple, uninterrupted spike from base to apex. |
| HNWP 64322 | *Elymus dahuricus* Turcz. | Qinghai, China | HNWP | No | None to very low | Not supported | The specimen contains several fertile culms, each terminating in an independent spike. All visible spikes are simple, unbranched, and borne singly at the apex of their respective culms. No lateral spike-bearing branches, secondary rachises, or compound inflorescence structures are present. The spikes vary slightly in length and density, but all share the same basic architecture: spikelets arranged directly along a single rachis. |
| HNWP 73291 | *Elymus dahuricus* Turcz. | Qinghai, China | HNWP | No | None | Not supported | The specimen preserves several fertile culms, each terminating in an individual spike. All spikes are simple, unbranched, and composed of spikelets attached directly to a single rachis. No lateral spike-bearing branches, secondary rachises, or compound inflorescence structures are visible. |
| HNWP 73546 | *Elymus dahuricus* Turcz. | Qinghai, China | HNWP | No | None | Not supported | The specimen consists of several fertile culms, each terminating in a single spike. The spikes are elongate, narrowly cylindrical, and structurally simple.  Spikelets are inserted directly along a single uninterrupted rachis. No lateral spike-bearing branches, secondary rachises, or branch nodes are visible.  The longest spike is relatively slender and somewhat lax, whereas the shorter spike is denser, but both share the same simple-spike architecture. |
| HNWP 98194 | *Elymus dahuricus* subsp. *cylindricus* (Franch.) N. R. Cui | Qinghai, China | HNWP | No | None to very weak | Not supported | The specimen consists of a dense tuft producing several fertile culms. Each culm terminates in a single spike. The spikes are slender, elongate, and moderately lax. The best-preserved spikes clearly show spikelets attached directly to a single rachis. No branch nodes, secondary rachises, or lateral spike-bearing axes are visible. Spike morphology is consistent across the specimen. |
| HNWP 243587 | *Elymus dahuricus* Turcz. | Qinghai, China | HNWP | No | None to very weak | Not supported | The specimen consists of a tuft producing several fertile culms. Three well-preserved spikes are visible. Each spike is borne terminally on an independent culm and exhibits a simple, unbranched architecture.  The spikes are relatively narrow, dark-colored, and moderately dense. Spikelets are inserted directly along a single rachis. No lateral spike-bearing branches, secondary rachises, or branching nodes can be observed. |
| N 019106438 | *Elymus dahuricus* Turcz. | Qinghai, China | N | No | None | Not supported | The specimen consists of several tufted culms bearing terminal spikes. Three spikes are preserved and clearly visible. Each spike is borne at the apex of an independent culm.  The spikes are slender, somewhat lax, and slightly nodding due to the long flexible peduncle. All spikelets are attached directly to a single rachis. No branching nodes, secondary rachises, or lateral spike-bearing structures are present. |
| SAUT 201400540 | *Elymus dahuricus* Turcz. ex Griseb. | Qinghai, China | SAUT | No | None | Not supported | The specimen preserves a single well-developed fertile spike together with vegetative shoots. The spike is elongate, narrow, and clearly unbranched. All spikelets are attached directly to a single continuous rachis. No branching nodes, lateral spike-bearing branches, secondary rachises, or compound spike structures are visible. |
| PE00489143 | *Elymus dahuricus Turcz.* | Xizang (Tibet), China | PE | No | None | Not supported | The specimen exhibits a single narrow, elongate spike with spikelets borne directly on a continuous central rachis. No branching nodes, secondary axes, or compound-spike structures are present. Apparent structural complexity results from post-maturation deformation rather than true morphological branching, and no evidence supports affinity with *Elymus chaobotryus*. |
| PE00489139 | *Elymus dahuricus* Turcz. | Tibet, China | PE | No | None | Not supported | The specimen exhibits several slender, lax, and elongate spikes with spikelets borne directly on a single continuous rachis. No branching nodes, secondary axes, or compound-spike structures are present. The observed lax spike morphology represents intraspecific variation within *Elymus dahuricus*, with no evidence of affinity to *Elymus chaobotryus*. |
| PE 00489136 | *Elymus dahuricus* Turcz. | Tibet, China | PE | No | None | Not supported | The specimen exhibits several slender, unbranched spikes with spikelets borne directly on a single continuous rachis. No evidence of branching, secondary axes, or compound spike organization is present. The morphology fits within the intraspecific variability of *Elymus dahuricus*, and no affinity with *Elymus chaobotryus* is supported. |
| PE 00489134 | *Elymus dahuricus* Turcz. | Tibet, China | PE | No | None | Not supported | The specimen presents multiple elongated, unbranched spikes arising from arching culms. Each spike consists of spikelets attached directly to a single continuous rachis without any branching or secondary axis formation. The observed complexity is geometric rather than structural. The morphology fully fits the variability of *Elymus dahuricus*, with no evidence supporting affinity to *Elymus chaobotryus*. |
| 00489131 | *Elymus dahuricus* Turcz. | Tibet, China | PE | No | None | Not supported | The specimen shows multiple elongated, unbranched spikes arising from separate culms. Each spike consists of spikelets directly inserted on a single continuous rachis without any evidence of branching, secondary axes, or compound organization. Observed heterogeneity in spike density reflects continuous morphological variation rather than structural differentiation. The specimen fits within the morphological range of *Elymus dahuricus*, and no evidence supports affinity with *Elymus chaobotryus*. |
| KUN1401490 | *Elymus dahuricus* Turcz. ex Griseb. | Tibet, China | KUN | No | None | Not supported | The specimen shows multiple tufted culms bearing elongated, unbranched spikes. Each spike consists of spikelets inserted directly on a single continuous rachis without any evidence of branching or secondary axis formation. Observed variability in density and apparent structural complexity arises from vegetative tufting and spatial overlap rather than inflorescence branching. The specimen conforms to the morphological range of *Elymus dahuricus*, and no evidence supports affinity with *Elymus chaobotryus*. |
| KUN 337222 | *Elymus dahuricus* Turcz. | Tibet, China | KUN | No | None | Not supported | The specimen exhibits multiple elongated, unbranched spikes borne on slender culms. Each spike consists of spikelets arranged sequentially along a single continuous rachis without any evidence of branching or secondary axis formation. Apparent structural complexity arises from culm curvature and spatial overlap rather than developmental branching. The morphology conforms to *Elymus dahuricus*, with no evidence supporting affinity with *Elymus chaobotryus*. |
| KUN 67032 | *Elymus dahuricus* Turcz. | Tibet, China | KUN | No | None | Not supported | The specimen consists of several basal culms bearing elongated, unbranched spikes. Each spike shows spikelets inserted directly along a single continuous rachis without any evidence of branching, secondary axis formation, or compound inflorescence architecture. Apparent curvature reflects mechanical deformation rather than developmental patterning. The specimen conforms to the morphological range of *Elymus dahuricus*, and no evidence supports affinity with *Elymus chaobotryus*. |
| HNWP 67236 | *Elymus dahuricus* Turcz. | Tibet, China | HNWP | No | None | Not supported | The specimen consists of tufted culms bearing elongated, unbranched spikes with spikelets inserted linearly along a single continuous rachis. No evidence of branching, secondary axis formation, or compound inflorescence structure is present. Observed curvature is attributable to post-collection deformation rather than developmental morphology. The specimen conforms to the typical morphological range of *Elymus dahuricus*, and no morphological evidence supports affinity with *Elymus chaobotryus*. |
| N019106429 | *Elymus dahuricus* Turcz. | Tibet, China | N | No | None | Not supported | The specimen consists of tufted culms bearing multiple elongated spikes, each characterized by a single continuous rachis with linearly arranged spikelets. Strong curvature and apparent looping of spikes are caused by mechanical deformation during pressing and drying rather than developmental branching. No evidence of compound inflorescence structure, secondary axes, or spike branching is observed. The specimen conforms to the typical morphological range of *Elymus dahuricus*, with no morphological support for affinity to *Elymus chaobotryus*. |
| FGC0002283 | *Elymus dahuricus* Turcz. | Tibet, China | FGC | No | None | Not supported | The specimen consists of tufted culms bearing elongated, unbranched spikes with spikelets arranged linearly along a single continuous rachis. Apparent curvature at the spike apex results from post-collection mechanical deformation rather than developmental architecture. No evidence of compound inflorescence formation, branching rachis structures, or intermediate branched-spike morphology is observed. The specimen is fully consistent with *Elymus dahuricus*, and no morphological support exists for affinity with *Elymus chaobotryus*. |
| PE 00489153 | *Elymus dahuricus* Turcz. | Tibet, China | PE | No | None | Not supported | The specimen consists of a tufted grass individual bearing a single elongated, slightly curved spike with spikelets arranged linearly along an unbranched rachis. Reduced spike density reflects developmental and environmental variation rather than structural modification. No evidence of compound inflorescence, branching rachis formation, or intermediate branched-spike morphology is observed. The specimen conforms to the morphological range of *Elymus dahuricus*, and no morphological support exists for affinity with *Elymus chaobotryus*. |
| PE 00489149 | *Elymus dahuricus* Turcz. | Tibet, China | PE | No | None | Not supported | The specimen consists of a tufted grass individual bearing a single slender, slightly flexuous spike with spikelets arranged linearly along an unbranched rachis. Apparent structural complexity is primarily caused by overlapping and folded vegetative leaves that partially obscure the reproductive axis. No evidence of compound inflorescence formation, branching rachis structures, or transitional branched-spike morphology is observed. The specimen conforms to the morphological range of *Elymus dahuricus*, with no support for affinity to *Elymus chaobotryus*. |
| PE 00489144 | *Elymus dahuricus* Turcz. | Tibet, China | PE | No | None | Not supported | The specimen consists of a tufted perennial grass bearing a single, slender, and flexuous spike with spikelets arranged linearly along an unbranched rachis. The apparent geometric complexity is entirely caused by strongly folded and angularly displaced vegetative leaf blades that overlap and cross the mounting surface. No anatomical evidence supports any branching inflorescence structure or transitional morphology toward a compound spike condition. The specimen is fully consistent with *Elymus dahuricus*, and no evidence supports affinity with *Elymus chaobotryus* or any intermediate form. |
| PE 00489138 | *Elymus dahuricus* Turcz. | Tibet, China | PE | No | None | Not supported | The specimen exhibits a typical tufted perennial grass habit with a single, elongate, slightly flexuous spike bearing spikelets arranged along an unbranched central rachis. Although vegetative leaves create partial visual occlusion and structural clutter, the reproductive axis remains morphologically simple and uninterrupted. No evidence supports branching, compound inflorescence formation, or transitional morphology toward *Elymus chaobotryus*. The specimen is fully consistent with the unbranched spike condition of *Elymus dahuricus*. |
| KUN 1401488 | *Elymus dahuricus* Turcz. ex Griseb. | Tibet, China | KUN | No | None | Not supported | The specimen exhibits a well-preserved, single, unbranched fertile spike arising from a tufted perennial grass base. Spikelets are continuously arranged along an intact central rachis without evidence of branching, compound organization, or transitional structural features. Despite minor curvature of the inflorescence, the architectural signal remains unequivocally uniaxial. The specimen is fully consistent with the canonical unbranched spike morphology of *Elymus dahuricus*, with no morphological support for affinity with *Elymus chaobotryus*. |
| HNWP 66514 | *Elymus dahuricus* Turcz. | Tibet, China | HNWP | No | None | Not supported | The specimen exhibits a tufted perennial grass with multiple independent fertile culms, each bearing a single, unbranched spike with a continuous central rachis. Although the presence of several spikes increases visual structural complexity, this does not correspond to inflorescence branching. All reproductive axes remain morphologically simple and uniaxial. No evidence supports compound spike formation or affinity with *Elymus chaobotryus*. |
| 67103 | *Elymus dahuricus* subsp. *cylindricus* (Franch.) N. R. Cui | Tibet, China | HNWP | No | None | Not supported | The specimen consists of a tufted perennial grass bearing multiple independent, cylindrical spikes, each formed by densely packed spikelets arranged along a single unbranched rachis. Although spike density produces a compact cylindrical appearance consistent with the subspecific epithet *cylindricus*, no structural evidence supports inflorescence branching or compound spike formation. All reproductive axes remain uniaxial, and no morphological indication of affinity with *Elymus chaobotryus* is observed. |
| KUN 1568542 | *Elymus dahuricus* Turcz. ex Griseb. | Tibet, China | KUN | No | None | Not supported | The specimen represents a small, weakly developed tufted grass individual bearing a single, slender fertile spike. The spike is unbranched and organized along a continuous central rachis, with no evidence of secondary axes or compound inflorescence formation. Although developmental limitation reduces overall morphological richness, the fundamental spike architecture remains consistent with the unbranched condition of *Elymus dahuricus*, with no support for affinity to *Elymus chaobotryus*. |
| KUN 1401486 | *Elymus dahuricus* Turcz. ex Griseb. | Tibet, China | KUN | No | None | Not supported | The specimen is a well-preserved tufted perennial grass bearing a clearly developed, moderately dense fertile spike. The spike is unbranched, with spikelets arranged continuously along a single central rachis. Although vegetative leaves are abundant and vertically oriented, they do not interfere with the interpretation of reproductive architecture. No evidence of branching, compound inflorescence formation, or transitional morphology is observed. The specimen conforms to the standard unbranched spike morphology of *Elymus dahuricus*. |
| PE 00489057 | *Elymus dahuricus* Turcz. | Xinjiang Uygur Autonomous Region, China | PE | No | None | Not supported | The specimen contains several mature fertile spikes that are narrow, elongate, and structurally simple. All spikelets are borne directly on a single central rachis, without lateral spike-bearing branches or secondary axes. No transitional morphology toward the branched-spike condition of *Elymus chaobotryus* is observed. The specimen conforms to the typical unbranched spike morphology of *Elymus dahuricus*. |
| PE00489056 | Elymus dahuricus | Xinjiang, China | PE | No | None | Not supported | The specimen contains mature fertile spikes with a simple, unbranched architecture. Spikelets are borne directly on a single central rachis, and no lateral spike-bearing branches or secondary axes are present. The specimen conforms to the typical morphology of *Elymus dahuricus* and shows no transitional features toward the branched-spike condition of *Elymus chaobotryus*. |
| PE00489053 | *Elymus dahuricus* Turcz. | Xinjiang Uygur Autonomous Region, China | PE | No | None | Not supported | The specimen contains mature fertile spikes with a simple and unbranched architecture. Spikelets are attached directly to a single central rachis without lateral spike-bearing branches or secondary axes. The specimen conforms to the typical morphology of *Elymus dahuricus* and shows no transitional features toward the branched-spike condition of *Elymus chaobotryus*. |
| PE 00489033 | *Elymus dahuricus* Turcz. | Xinjiang Uygur Autonomous Region, China | PE | No | None | Not supported | The specimen contains mature fertile individuals of *Elymus dahuricus* with typical simple spike inflorescences. The spikelets are arranged directly along a single continuous rachis, without lateral spike-bearing branches, secondary axes, or compound spike organization.  The specimen conforms to the typical morphology of *Elymus dahuricus* and provides no evidence of transitional morphology toward the branched-spike architecture characteristic of *Elymus chaobotryus*. |
| PE 00489023 | *Elymus dahuricus* Turcz. | Xinjiang Uygur Autonomous Region, China | PE | No | None | Not supported | The specimen contains fertile individuals of *Elymus dahuricus* with typical simple spike inflorescences. The spikelets are arranged directly along a single continuous rachis without lateral spike-bearing branches, secondary axes, or compound spike organization.  The specimen conforms to the typical morphology of *Elymus dahuricus* and shows no transitional features toward the branched-spike architecture characteristic of *Elymus chaobotryus*. |
| KUN337227 | *Elymus dahuricus* Turcz. | Xinjiang, China | KUN | No | None | Not supported | The specimen contains fertile individuals of *Elymus dahuricus* with a typical simple spike inflorescence. The spikelets are arranged directly along a single continuous rachis, without lateral spike-bearing branches, secondary axes, or compound spike organization.  No true rachis branching is observed.  The specimen conforms to the typical morphology of *Elymus dahuricus* and shows no transitional features toward the branched-spike architecture characteristic of *Elymus chaobotryus*. |
| HNWP 45211 | *Elymus dahuricus* Turcz. | Xinjiang Uygur Autonomous Region, China | HNWP | No | None | Not supported | The specimen contains mature fertile individuals of *Elymus dahuricus* with typical simple spike inflorescences. Spikelets are arranged directly along a single continuous rachis without lateral spike-bearing branches, secondary axes, or compound spike organization. The specimen conforms to the typical morphology of *Elymus dahuricus* and shows no transitional features toward the branched-spike architecture characteristic of *Elymus chaobotryus*. |
| BJFC00004723 | *Elymus dahuricus* var. *cylindricus* Franch. | Xinjiang Uygur Autonomous Region, China | BJFC | No | None | Not supported | This specimen preserves a well-developed cylindrical spike of *Elymus dahuricus* var. *cylindricus*. The inflorescence consists of a single continuous rachis bearing spikelets directly along the main axis, with no lateral spike-bearing branchlets or secondary rachis axes. The absence of rachis-derived lateral spike units confirms that this specimen does not approach the branched-spike architecture characteristic of *Elymus chaobotryus*. |
| PE00489051 | *Elymus dahuricus* Turcz. | Xinjiang Uygur Autonomous Region, China | PE | No | None | Not supported | This specimen preserves several mature flowering culms of *Elymus dahuricus*, including clearly visible simple spikes. Although multiple spike-bearing structures occur on the herbarium sheet, each spike represents an independent unbranched inflorescence arising from a separate culm. The preserved spikes maintain a single continuous rachis with spikelets attached directly along the main axis, without lateral spike-bearing branchlets or secondary rachis axes. |
| PE00489052 | *Elymus dahuricus* Turcz. | Xinjiang Uygur Autonomous Region, China | PE | No | None | Not supported | This specimen preserves a mature elongated spike of *Elymus dahuricus* with a clearly recognizable simple-spike architecture. The spikelets are arranged directly along a single continuous rachis, and the inflorescence axis remains completely unbranched. Although several culms and elongated leaves are present on the herbarium sheet, these structures represent separate vegetative organs rather than lateral spike-bearing branches. |
| PE00489038 | *Elymus dahuricus* Turcz. | Xinjiang Uygur Autonomous Region, China | PE | No | None | Not supported | This specimen preserves multiple mature flowering culms of *Elymus dahuricus*, with two well-developed spikes clearly visible on the herbarium sheet. Although the presence of several spike-bearing structures may superficially suggest increased inflorescence complexity, detailed examination shows that each spike develops independently from a separate culm rather than from branching of a common rachis. Each spike maintains a single continuous rachis with spikelets attached directly along the main axis, and no lateral spike-bearing branchlets or secondary rachis axes are observed. The specimen therefore represents the normal multi-culm simple-spike condition of *Elymus dahuricus*, rather than a compound branched inflorescence. |
| 00489026 | *Elymus dahuricus* Turcz. | Xinjiang Uygur Autonomous Region, China | PE | No | None | Not supported | This specimen preserves several mature flowering culms of *Elymus dahuricus*, including a clearly visible elongated simple spike. Although multiple culms occur within the same herbarium specimen and create a visually complex arrangement, the spike-bearing structures are independent shoots rather than branches produced from a common rachis. The preserved spike maintains a single continuous rachis with spikelets directly attached along the main axis, without lateral spike-bearing branchlets or secondary rachis axes. The specimen therefore represents the normal multi-culm simple-spike condition of *Elymus dahuricus* and shows no structural transition toward the compound branched-spike architecture characteristic of *Elymus chaobotryus*. |
| HNWP 44668 | *Elymus dahuricus* Turcz. | Xinjiang Uygur Autonomous Region, China | HNWP | No | None | Not supported | This specimen preserves a multi-culm individual of *Elymus dahuricus* with several mature simple spikes. Although numerous spike-bearing structures occur within the same herbarium sheet and create a visually complex arrangement, each spike develops independently from a separate flowering culm rather than through branching of a common rachis. The preserved spikes maintain single continuous rachises with spikelets attached directly along the main axes, without lateral spike-bearing branchlets or secondary rachis development. The specimen therefore represents the normal multi-culm simple-spike condition of *Elymus dahuricus* and provides no evidence of transition toward the compound branched-spike architecture characteristic of *Elymus chaobotryus*. |
| BJFC00004732 | *Elymus dahuricus* Turcz. ex Griseb. | Xinjiang Uygur Autonomous Region, China | BJFC | No | None | Not supported | This specimen preserves a relatively isolated mature spike of *Elymus dahuricus*, allowing direct assessment of its inflorescence architecture. The spike consists of a single continuous rachis bearing spikelets directly along the main axis, without lateral spike-bearing branchlets or secondary rachis development. The elongated spike form represents normal variation within the simple-spike morphology of *Elymus dahuricus* rather than a modification toward compound spike formation. The specimen provides no evidence of transitional morphology toward the rachis-branched inflorescence architecture characteristic of *Elymus chaobotryus*. |
| PE00489054 | *Elymus dahuricus* Turcz. | Xinjiang Uygur Autonomous Region, China | PE | No | None | Not supported | This specimen preserves a multi-culm individual of *Elymus dahuricus* with several mature simple spikes. Each spike represents an independent unbranched inflorescence arising from a separate culm. The preserved spikes maintain single continuous rachises with spikelets directly attached along the main axes, without lateral spike-bearing branchlets or secondary rachis development. The specimen therefore illustrates the normal variation of whole-plant architecture in *Elymus dahuricus* and provides no evidence of structural transition toward the compound branched-spike architecture characteristic of *Elymus chaobotryus*. |
| PE00489039 | *Elymus dahuricus* Turcz. | Xinjiang Uygur Autonomous Region, China | PE | No | None | Not supported | This specimen preserves a multi-culm individual of *Elymus dahuricus* with several mature simple spikes distributed across the herbarium sheet. Each spike represents an independent unbranched inflorescence arising from a separate culm. The spikelets remain directly attached along single continuous rachises, and no rachis-derived lateral spike-bearing branchlets or secondary spike axes are present. The specimen therefore demonstrates the diversity of whole-plant architecture within *Elymus dahuricus* without any modification of the inflorescence structure. No transitional features toward the compound branched-spike architecture characteristic of *Elymus chaobotryus* are observed. |
| PE00489029 | *Elymus dahuricus* Turcz. | Xinjiang Uygur Autonomous Region, China | PE | No | None | Not supported | This specimen preserves a robust individual of *Elymus dahuricus* with multiple flowering culms and a clearly visible mature simple spike. The reproductive structure remains organized as a single-axis spike. Spikelets are attached directly along one continuous rachis, with no lateral spike-bearing branchlets or secondary rachis axes observed. No structural evidence indicates transition toward the compound branched-spike architecture characteristic of *Elymus chaobotryus*. |
| PE00489027 | *Elymus dahuricus* Turcz. | Xinjiang Uygur Autonomous Region, China | PE | No | None | Not supported | This specimen preserves an individual of *Elymus dahuricus* with a clearly visible isolated mature simple spike. The spike consists of a single continuous rachis bearing spikelets directly along the main axis, without lateral spike-bearing branchlets or secondary rachis development. The specimen therefore represents a slender unbranched spike form of *Elymus dahuricus* and provides no evidence of transition toward the compound branched-spike architecture characteristic of *Elymus chaobotryus*. |
| KUN1401470 | *Elymus dahuricus* Turcz. ex Griseb. | Xinjiang Uygur Autonomous Region, China | KUN | No | None | Not supported | This specimen preserves a relatively complete individual of *Elymus dahuricus* with a clearly visible mature simple spike. The spike exhibits a single continuous rachis bearing spikelets directly along the main axis, without lateral spike-bearing branchlets or secondary rachis development. The specimen therefore represents the typical unbranched spike morphology of *Elymus dahuricus* and provides no evidence of transition toward the compound branched-spike architecture characteristic of *Elymus chaobotryus*. |
| KUN337225 | *Elymus dahuricus* Turcz. | Xinjiang Uygur Autonomous Region, China | KUN | No | None | Not supported | This specimen preserves a vigorous individual of *Elymus dahuricus* with multiple flowering culms and several mature simple spikes. Each spike develops independently from a separate culm and retains a single continuous rachis structure. Spikelets are directly attached along the main rachis, with no lateral spike-bearing branchlets or secondary rachis axes observed. The specimen therefore represents a multi-spike growth form of *Elymus dahuricus* in which whole-plant architecture varies while inflorescence organization remains simple and unbranched. No evidence supports a transitional relationship toward the compound branched-spike architecture characteristic of *Elymus chaobotryus*. |
| KUN 337226 | *Elymus dahuricus* Turcz. | Xinjiang Uygur Autonomous Region, China | KUN | No | None | Not supported | This specimen preserves a multi-culm individual of *Elymus dahuricus* with a clearly visible mature simple spike. The inflorescence itself remains structurally independent and unbranched. The spike consists of a single continuous rachis bearing spikelets directly along the main axis, without lateral spike-bearing branchlets or secondary rachis development. The specimen therefore demonstrates variation in whole-plant growth form while maintaining the typical simple-spike architecture of *Elymus dahuricus*. No evidence supports a transitional relationship toward the compound branched-spike architecture characteristic of *Elymus chaobotryus*. |
| NAS00520432 | *Elymus dahuricus* Turcz. | Xinjiang Uygur Autonomous Region, China | NAS | No | None | Not supported | This specimen preserves a robust tufted individual of *Elymus dahuricus* with numerous flowering culms and multiple mature simple spikes. Each spike possesses a single continuous rachis with spikelets directly attached along the main axis, without lateral spike-bearing branchlets or secondary rachis development. The specimen therefore demonstrates extensive variation in vegetative architecture and tiller production within *Elymus dahuricus* while maintaining the typical simple-spike condition. No evidence supports a transitional relationship toward the rachis-branched compound spike architecture characteristic of *Elymus chaobotryus*. |
| BJFC00004731 | *Elymus dahuricus* Turcz. ex Griseb. | Xinjiang Uygur Autonomous Region, China | BJFC | No | None | Not supported | This specimen preserves an isolated mature spike of *Elymus dahuricus* with a clearly traceable unbranched inflorescence axis. The spike consists of a single continuous rachis bearing spikelets directly along the main axis, without lateral spike-bearing branchlets or secondary rachis development. The specimen therefore provides a clear example of the typical simple-spike morphology of *Elymus dahuricus* and shows no evidence of transition toward the compound branched-spike architecture characteristic of *Elymus chaobotryus*. |
| PE00489055 | *Elymus dahuricus* Turcz. | Xinjiang Uygur Autonomous Region, China | PE | No | None | Not supported | This specimen preserves a robust individual of *Elymus dahuricus* with multiple flowering culms and several mature simple spikes. The spikelets remain directly attached along single continuous rachises, and no lateral spike-bearing branchlets or secondary rachis axes are observed. The specimen therefore illustrates the range of whole-plant architectural variation in *Elymus dahuricus* while maintaining a stable simple-spike inflorescence pattern. No evidence supports a transitional relationship toward the compound branched-spike architecture characteristic of *Elymus chaobotryus*. |
| PE00489036 | *Elymus dahuricus* Turcz. | Xinjiang Uygur Autonomous Region, China | PE | No | None | Not supported | This specimen preserves a robust individual of *Elymus dahuricus* with multiple flowering culms and several mature spikes. The spikelets are directly attached along single continuous rachises, and no lateral spike-bearing branchlets or secondary rachis axes are observed. The specimen therefore demonstrates extensive variation in plant architecture while maintaining the stable unbranched spike condition characteristic of *Elymus dahuricus*. No evidence supports a transitional relationship toward the compound branched-spike architecture of *Elymus chaobotryus*. |
| PE 00489028 | *Elymus dahuricus* Turcz. | Xinjiang Uygur Autonomous Region, China | PE | No | None | Not supported | This specimen preserves a fertile individual of *Elymus dahuricus* with a typical simple spike and a multi-culm vegetative architecture. No lateral spike-bearing branchlets, secondary rachis axes, or compound spike organization are observed. The specimen therefore demonstrates variation in vegetative growth form while maintaining the characteristic simple-spike architecture of *Elymus dahuricus*. No evidence supports a transitional relationship toward the compound branched-spike architecture characteristic of *Elymus chaobotryus*. |
| PE 00489024 | *Elymus dahuricus* Turcz. | Xinjiang Uygur Autonomous Region, China | PE | No | None | Not supported | This specimen preserves a fertile individual of *Elymus dahuricus* with multiple flowering culms and two mature simple spikes. Each spike possesses a single continuous rachis bearing directly attached spikelets, without lateral spike-bearing branchlets or secondary rachis axes. The specimen therefore illustrates variation in perennial growth form and flowering culm number while maintaining the typical unbranched spike architecture of *Elymus dahuricus*. No evidence supports a transitional relationship toward the compound branched-spike architecture characteristic of *Elymus chaobotryus*. |
| SAUT201400543 | *Elymus dahuricus* Turcz. ex Griseb. | Xinjiang Uygur Autonomous Region, China | SAUT | No | None | Not supported | This specimen preserves a well-isolated flowering culm of *Elymus dahuricus*, allowing the complete relationship between the culm and the terminal spike to be evaluated without interference from overlapping shoots. The mature spike is supported by a single continuous rachis bearing directly attached spikelets, and neither rachis branching nor secondary spike axes are present. The gentle curvature of the culm represents natural growth and herbarium preservation rather than modification of the reproductive axis. Consequently, this specimen provides a particularly clear demonstration of the canonical simple-spike architecture of *Elymus dahuricus* and offers no evidence of morphological transition toward the compound branched-spike organization characteristic of *Elymus chaobotryus*. |
| PE00489034 | *Elymus dahuricus* Turcz. | Xinjiang Uygur Autonomous Region, China | PE | No | None | Not supported | This specimen preserves three mature flowering culms of *Elymus dahuricus*, each terminating in an independent simple spike. Every spike is supported by a single uninterrupted rachis bearing directly attached spikelets, with no lateral spike-bearing branchlets, secondary rachis axes, or compound spike organization observed. The specimen therefore illustrates that variation in culm architecture is not accompanied by variation in inflorescence architecture, reinforcing the stable simple-spike morphology characteristic of *Elymus dahuricus*. No evidence supports a transitional relationship toward the branched-spike condition of *Elymus chaobotryus*. |
| PE00489044 | *Elymus dahuricus* Turcz. | Xinjiang Uygur Autonomous Region, China | PE | No | None | Not supported | This specimen preserves two exceptionally long, mature spikes of *Elymus dahuricus*, both displaying a conspicuously pendulous habit. Structural examination demonstrates that both spikes possess a single uninterrupted rachis with spikelets inserted directly along the primary axis, without lateral spike-bearing branchlets, secondary rachis axes, or any indication of compound spike organization. The specimen clearly demonstrates that pendulous spike posture does not alter the underlying inflorescence architecture and remains fully consistent with the stable simple-spike morphology of *Elymus dahuricus*, providing no support for a transitional relationship with the branched-spike condition characteristic of *Elymus chaobotryus*. |
| PE00489043 | *lymus dahuricus* Turcz. | Xinjiang Uygur Autonomous Region, China | PE | No | None | Not supported | This specimen preserves a single well-developed fertile spike of *Elymus dahuricus*, providing a clear view of the complete inflorescence architecture. No lateral spike-bearing branchlets, secondary rachis axes, or compound spike organization are present. The specimen demonstrates that variation at the spike apex reflects normal developmental or preservation-related morphology rather than genuine branching, and it fully conforms to the stable simple-spike architecture characteristic of *Elymus dahuricus*. |
| N019106469 | *lymus dahuricus* Turcz. | Xinjiang Uygur Autonomous Region, China | N | No | None | Not supported | This specimen preserves two mature spikes of *Elymus dahuricus* that are mounted separately and remain free from overlap, providing an unusually clear view of the reproductive architecture. Each spike is supported by a single uninterrupted rachis bearing sessile spikelets directly along the primary axis, and neither displays lateral spike-bearing branchlets, secondary rachis axes, nor any indication of compound spike organization. The specimen therefore represents a clear reference example of the stable simple-spike morphology of *Elymus dahuricus* and offers no support for a transitional relationship with the branched-spike condition of *Elymus chaobotryus*. |
| PE00489031 | *Elymus dahuricus* Turcz. | Xinjiang Uygur Autonomous Region, China | PE | No | None | Not supported | This specimen preserves four mature spikes of *Elymus dahuricus* arising from a single tuft, allowing direct comparison among multiple flowering shoots within one individual. No lateral spike-bearing branchlets, secondary rachis axes, or compound spike organization are observed in any of the preserved spikes. The specimen highlights the remarkable structural uniformity of the simple-spike architecture across multiple reproductive culms and provides strong evidence against any transition toward the branched-spike condition characteristic of *Elymus chaobotryus*. |
| PE00489032 | *Elymus dahuricus* Turcz. | Xinjiang Uygur Autonomous Region, China | PE | No | None | Not supported | This specimen preserves three mature spikes of *Elymus dahuricus* arising from a single tuft. No lateral spike-bearing branchlets, secondary rachis axes, or compound spike organization are present. The specimen therefore represents a typical expression of *Elymus dahuricus* and provides no support for transitional morphology toward the branched-spike condition of *Elymus chaobotryus*. |
| BJFC00004724 | *Elymus dahuricus* var. *cylindricus* Franch. | Xinjiang Uygur Autonomous Region, China | BJFC | No | None | Not supported | This specimen preserves a single mature spike of *Elymus dahuricus* var. *cylindricus*. The inflorescence retains a typical simple-spike architecture composed of one continuous rachis bearing sessile spikelets directly along the main axis. No lateral spike-bearing branchlets, secondary rachis axes, or compound spike organization are present. The specimen therefore provides no evidence of transitional morphology toward the branched-spike architecture characteristic of *Elymus chaobotryus*. |
| FGC0002366 | *Elymus dahuricus* Turcz. | Xinjiang Uygur Autonomous Region, China | FGC | No | None | Not supported | This specimen preserves a well-developed flowering shoot of *Elymus dahuricus* with a single mature spike. The inflorescence is composed of one continuous rachis bearing sessile spikelets directly along its length. No lateral spike-bearing branchlets, secondary rachis axes, or compound spike organization are present. The specimen therefore conforms to the typical simple-spike morphology of *Elymus dahuricus* and provides no evidence of transitional morphology toward the compound branched-spike architecture characteristic of *Elymus chaobotryus*. |
| HNWP44509 | *Elymus dahuricus* Turcz. | Xinjiang Uygur Autonomous Region, China | HNWP | No | None | Not supported | This specimen preserves a mature flowering culm of *Elymus dahuricus* bearing a single simple spike. The inflorescence consists of one continuous rachis with sessile spikelets arranged directly along the main axis. Neither lateral spike-bearing branchlets nor secondary rachis axes are present, and no compound spike organization can be observed. The specimen therefore represents a typical expression of the simple-spike architecture of *Elymus dahuricus* and provides no evidence of transitional morphology toward the compound branched-spike condition characteristic of *Elymus chaobotryus*. |
| PE00489004 | *Elymus dahuricus* Turcz. | Gansu, China | PE | No | None | Not supported | This specimen preserves a mature flowering individual of *Elymus dahuricus* with a single well-developed spike. The inflorescence is composed of one continuous rachis bearing sessile spikelets directly along its length. No lateral spike-bearing branchlets, secondary rachis axes, or compound spike organization are observed. The specimen therefore represents a typical simple-spike form of *Elymus dahuricus* and provides no evidence of transitional morphology toward the compound branched-spike architecture characteristic of *Elymus chaobotryus*. |
| PE00488998 | *Elymus dahuricus* Turcz. | Gansu, China | PE | No | Very low | None | The specimen bears a single, continuous, unbranched spike. Spikelets are inserted regularly along the primary rachis, and no rachis node gives rise to lateral spike-bearing branchlets. No secondary spike axis, repeated branching, or compound spike framework is present. The slight curvature of the spike is attributable to natural bending or specimen preparation rather than branching. Overall, the specimen conforms to the typical spike morphology of *Elymus dahuricus* and shows no transitional features toward *E. chaobotryus*. |
| PE 00488997 | *Elymus dahuricus* Turcz. | Gansu, China | PE | No | Very low | None | The specimen exhibits a typical simple spike morphology of *Elymus dahuricus*. The inflorescence consists of a single continuous rachis with spikelets attached directly along the primary axis. No lateral spike-bearing branchlets, secondary rachises, or compound spike architecture are observed.  The curved appearance of the spike is interpreted as natural bending during maturation or specimen pressing rather than a branching feature.  No transitional characters toward *Elymus chaobotryus* are present. |
| PE 00488993 | *Elymus dahuricus* Turcz. | Gansu, China | PE | No | Very low | None | The specimen exhibits a typical simple spike morphology of *Elymus dahuricus*. The inflorescences consist of single continuous rachises with spikelets directly attached along the main axis. No lateral spike-bearing branchlets, secondary rachises, or compound spike structures are observed.  Although the spikes are curved and slightly drooping, this represents normal mature spike bending or pressing deformation rather than rachis branching.  No transitional morphology toward *Elymus chaobotryus* is present. |
| PE 00488979 | *Elymus dahuricus* Turcz. | Gansu, China | PE | No | Very low | None | The specimen represents a typical simple spike morphology of *Elymus dahuricus*. The inflorescences consist of single continuous rachises with spikelets directly attached along the main axis. No lateral spike-bearing branchlets, secondary rachises, or compound spike structures are observed.  The curved and drooping appearance of the spikes is interpreted as a normal mature spike feature or specimen pressing effect rather than true rachis branching.  No transitional characters toward *Elymus chaobotryus* are present. |
| PE00488974 | *Elymus dahuricus* Turcz. | Gansu, China | PE | No | Very low | None | The specimen represents a typical simple-spike morphology of *Elymus dahuricus*. The inflorescence consists of a single continuous rachis with spikelets directly attached along the main axis. No lateral spike-bearing branchlets, secondary rachises, or compound spike architecture are observed.  The specimen lacks diagnostic characters associated with *Elymus chaobotryus*, including rachis branching, lateral spike-bearing branches, and hierarchical inflorescence organization.  No transitional characters toward the branched-spike morphology are detected. |
| PE00488973 | *Elymus dahuricus* Turcz. | Gansu, China | PE | No | Very low | None | The specimen represents a typical simple-spike form of *Elymus dahuricus*. The inflorescence consists of a single rachis with spikelets arranged directly along the main axis. No lateral spike-bearing branches, secondary rachises, or compound spike architecture are observed.  The specimen lacks the diagnostic branched-spike characters of *Elymus chaobotryus*, including basal rachis branching and lateral spike-bearing branchlets.  No transitional morphology toward a compound spike structure is detected. |
| PE 00488972 | *Elymus dahuricus* Turcz. | Gansu, China | PE | No | Very low | None | The specimen represents a typical simple-spike morphology of *Elymus dahuricus*. The inflorescences consist of a single rachis with spikelets directly attached along the main axis. No lateral spike-bearing branches or secondary rachises are observed.  No transitional characters toward branched spike development are detected. |
| PE00488966 | *Elymus dahuricus* Turcz. | Gansu, China | PE | No | Very low | None | The specimen shows a typical unbranched spike of *Elymus dahuricus*. The inflorescence consists of a single continuous rachis with spikelets directly attached along the primary axis. No lateral spike-bearing branchlets, secondary axes, or compound spike architecture are observed. The curved appearance of the spike is due to natural bending during growth or specimen pressing rather than rachis branching.  The specimen therefore provides no evidence of transitional morphology toward *Elymus chaobotryus*. |
| KUN 337234 | *Elymus dahuricus* Turcz. | Gansu, China | KUN | No | Very low | None | The specimen represents a typical simple-spike form of *Elymus dahuricus*. The inflorescence is composed of a single rachis with spikelets directly attached along the main axis. No lateral spike-bearing branches, secondary rachises, or compound spike architecture are observed.  No transitional characters toward the branched compound spike morphology of *Elymus chaobotryus* are detected. |
| HNWP 166934 | *Elymus dahuricus* Turcz. | Gansu, China | HNWP | No | Very low | None | The specimen represents a typical simple-spike form of *Elymus dahuricus*. The inflorescence consists of a single rachis with spikelets directly attached along the main axis. No lateral spike-bearing branches or secondary rachises are observed.  The reproductive architecture is clearly different from the branched compound spike characteristic of *Elymus chaobotryus*. No intermediate morphological traits toward compound spike development are detected. |
| N019106478 | *Elymus dahuricus* subsp. *cylindricus* (Franch.) N. R. Cui | Gansu, China | N | No | Very low | None | The specimen represents a cylindrical simple-spike form of *Elymus dahuricus* subsp. *cylindricus*. The inflorescence consists of a single rachis with spikelets directly attached along the main axis.  No lateral spike-bearing branches, secondary rachises, or compound spike structures are observed. Therefore, the specimen does not show morphological affinity toward the branched spike architecture of *Elymus chaobotryus*. |
| FGC0002265 | *Elymus dahuricus* Turcz. | Gansu, China | FGC | No | Very low | None | The specimen represents a typical simple-spike form of *Elymus dahuricus*. The inflorescence consists of a single rachis with spikelets directly attached along the main axis.  No lateral spike-bearing branches, secondary rachises, or compound spike structures are observed. The inflorescence architecture is clearly different from the branched compound spike of *Elymus chaobotryus*. |
| FGC0002302 | *Elymus dahuricus* Turcz. | Gansu, China | FGC | No | Low | None | The specimen represents a typical simple-spike form of *Elymus dahuricus*. The inflorescence consists of a single elongated rachis with spikelets directly attached along the main axis.  No lateral spike-bearing branches or secondary rachises are observed. The architecture differs markedly from the branched compound spike characteristic of *Elymus chaobotryus*. No intermediate morphology toward compound spike development is detected. |
| FGC0002331 | *Elymus dahuricus* Turcz. | Gansu, China | FGC | No | Very low | None | The specimen represents a typical simple-spike form of *Elymus dahuricus*. The inflorescence consists of a single elongated rachis with spikelets directly attached along the main axis.  No lateral spike-bearing branches or secondary rachises are observed. The inflorescence architecture is clearly different from the branched compound spike of *Elymus chaobotryus*. No transitional structures toward a compound spike are detected. |
| PE02078299 | *Elymus dahuricus* Turcz. | Gansu, China | PE | No | Very low | None | The specimen represents a typical simple-spike form of *Elymus dahuricus*. The inflorescence is composed of a single elongated rachis with spikelets directly attached along the main axis.  No lateral spike-bearing branches, secondary rachises, or compound spike structures are observed. The specimen does not show intermediate morphology toward the branched spike architecture of *Elymus chaobotryus*. |
| PE00489002 | *Elymus dahuricus* Turcz. | Gansu, China | PE | No | Very low | None | The specimen represents a typical simple-spike morphology of *Elymus dahuricus*. The inflorescence consists of a single elongated rachis without lateral branches or secondary spike-bearing axes.  No morphological evidence of compound spike development or transitional architecture toward *Elymus chaobotryus* is observed. |
| PE00488986 | *Elymus dahuricus* Turcz. | Gansu, China | PE | No | Very low | None | The specimen represents a typical simple-spike form of *Elymus dahuricus*. Multiple culms and inflorescences are present, but all spikes are composed of a single rachis without lateral spike-bearing branches.  No compound spike architecture or intermediate branching morphology toward *Elymus chaobotryus* is observed. |
| PE 00488983 | *Elymus dahuricus* Turcz. | Gansu, China | PE | No | Low | None | The specimen represents a typical simple-spike morphology of *Elymus dahuricus*. Although the plant forms numerous tillers and several flowering culms, all inflorescences remain unbranched.  No lateral spike-bearing branches or compound spike architecture are observed. The specimen does not represent a transitional form toward the branched-spike morphology of *Elymus chaobotryus*. |
| PE 00488982 | *Elymus dahuricus* Turcz. | Gansu, China | PE | No | Low | None | The specimen represents a typical simple-spike form of *Elymus dahuricus*. The plant has multiple tillers and several flowering culms, but all inflorescences remain unbranched.  No lateral spike-bearing branches, compound spike structures, or intermediate branching characters toward *Elymus chaobotryus* are observed. |
| PE 00488981 | *Elymus dahuricus* Turcz. | Gansu, China | PE | No | Very Low | None | The specimen represents a typical unbranched simple-spike form of *Elymus dahuricus*.  No lateral spike-bearing branches, compound spike structures, or intermediate branching characters toward the branched-spike morphology of *Elymus chaobotryus* are observed. |
| HNWP 166547 | *Elymus dahuricus* Turcz. | Gansu, China | HNWP | No | Very Low | None | The specimen represents a typical simple-spike form of *Elymus dahuricus*. The inflorescences consist of single continuous rachises with spikelets directly attached along the main axis.  No lateral spike-bearing branchlets, secondary rachises, or compound spike structures are observed. The specimen does not show transitional morphology toward the branched spike architecture of *Elymus chaobotryus*. |
| N019106489 | *Elymus dahuricus* subsp. *cylindricus* (Franch.) N. R. Cui | Gansu, China | N | No | Very Low | None | The specimen represents a cylindrical simple-spike form of *Elymus dahuricus* subsp. *cylindricus*. The inflorescence consists of a single elongated cylindrical rachis with spikelets directly attached along the main axis.  No lateral spike-bearing branchlets, secondary rachises, or compound spike structures are observed. Therefore, the specimen does not show morphological affinity toward the branched spike architecture of *Elymus chaobotryus*.  The elongated and slightly curved appearance of the spikes is interpreted as a normal cylindrical spike feature and/or a result of herbarium pressing, rather than true rachis branching. |
| SAUT 201400541 | *Elymus dahuricus* Turcz. ex Griseb. | Gansu, China | SAUT | No | Very Low | None | The specimen represents a typical simple-spike form of *Elymus dahuricus*. The inflorescence consists of a single elongated rachis with spikelets directly attached along the main axis.  No lateral spike-bearing branchlets, secondary rachises, or compound spike structures are observed. The specimen does not show transitional morphology toward the branched spike architecture of *Elymus chaobotryus*.  The slightly curved appearance of the spike is interpreted as a normal mature spike feature and/or a result of herbarium pressing, rather than true rachis branching. |
| PE00488976 | *Elymus dahuricus* Turcz. | Gansu, China | PE | No | Very Low | None | The specimen represents a typical simple-spike form of *Elymus dahuricus*. The inflorescences consist of single elongated rachises with spikelets directly attached along the main axis.  No lateral spike-bearing branchlets, secondary rachises, or compound spike structures are observed. The specimen does not show transitional morphology toward the branched spike architecture of *Elymus chaobotryus*. |
| KUN 337233 | *Elymus dahuricus* Turcz. | Gansu, China | KUN | No | Very Low | None | The specimen represents a typical simple-spike form of *Elymus dahuricus*. The inflorescences consist of single elongated rachises with spikelets directly attached along the main axis.  No lateral spike-bearing branchlets, secondary rachises, or compound spike structures are observed. The specimen does not show transitional morphology toward the branched spike architecture of *Elymus chaobotryus*.  The slightly curved and drooping appearance of the spikes is interpreted as a normal mature spike feature and/or a result of herbarium pressing, rather than true rachis branching. The overall inflorescence architecture remains consistent with a simple spike form. |
| PE00488999 | *Elymus dahuricus* Turcz. | Gansu, China | PE | No | Very Low | None | The specimen represents a typical simple-spike form of *Elymus dahuricus*. The inflorescences consist of single continuous rachises with spikelets directly attached along the main axis.  No lateral spike-bearing branchlets, secondary rachises, or compound spike structures are observed. The specimen does not show transitional morphology toward the branched spike architecture of *Elymus chaobotryus*.  The curved and drooping appearance of the spikes is interpreted as a normal mature spike feature and/or a result of herbarium pressing, rather than true rachis branching. The overall inflorescence architecture remains consistent with a simple spike form. |
| KUN 337230 | *Elymus dahuricus* Turcz. | Gansu, China | KUN | No | Very Low | None | The specimen represents a typical simple-spike form of *Elymus dahuricus*. The inflorescence consists of a single continuous rachis with spikelets directly attached along the main axis.  No lateral spike-bearing branchlets, secondary rachises, or compound spike structures are observed. The specimen does not show transitional morphology toward the branched spike architecture of *Elymus chaobotryus*.  The overall inflorescence architecture remains consistent with a simple spike form. |
| PE00489047 | *Elymus dahuricus* Turcz. | Gansu, China | PE | No | Very Low | None | The specimen represents a typical simple-spike form of *Elymus dahuricus*. The inflorescences consist of single elongated rachises with spikelets directly attached along the main axis.  Several flowering culms are present, but all spikes remain unbranched. No lateral spike-bearing branchlets, secondary rachises, or compound spike structures are observed.  The specimen shows no transitional morphology toward the branched spike architecture of *Elymus chaobotryus*. |
| PE00488964 | *Elymus dahuricus* Turcz. | Gansu, China | PE | No | Very Low | None | The specimen represents a typical simple-spike form of *Elymus dahuricus*. The inflorescences consist of single elongated rachises with spikelets directly attached along the main axis.  No lateral spike-bearing branchlets, secondary rachises, or compound spike structures are observed. The specimen shows no transitional morphology toward the branched spike architecture of *Elymus chaobotryus*. |
| NWTC0094637 | *Elymus dahuricus* var. *violeus* C. P. Wang & H. L. Yang | Gansu, China | NWTC | No | Very Low | None | The specimen represents a typical simple-spike form of *Elymus dahuricus* var. *violeus*. The inflorescences consist of single elongated rachises with spikelets directly attached along the main axis.  Although multiple flowering culms are present, all visible spikes remain unbranched. No lateral spike-bearing branchlets, secondary rachises, or compound spike structures are observed.  The specimen shows no transitional morphology toward the branched spike architecture of *Elymus chaobotryus*. |
| N019106479 | *Elymus dahuricus* subsp. *cylindricus* (Franch.) N. R. Cui | Gansu, China | N | No | Very Low | None | The specimen represents a typical cylindrical simple-spike form of *Elymus dahuricus* subsp. *cylindricus*. The inflorescence consists of a single elongated rachis with spikelets directly attached along the main axis.  The compact cylindrical spike appearance is interpreted as a characteristic feature of this taxon rather than a transitional form. The specimen shows no morphological affinity toward the branched spike architecture of *Elymus chaobotryus*. |
| PE00488769 | *Elymus dahuricus* Turcz. | Inner Mongolia Autonomous Region, China | PE | No | Very Low | None | The specimen represents a typical simple-spike form of *Elymus dahuricus*. The inflorescences consist of single elongated rachises with spikelets directly attached along the main axis.  Although multiple flowering culms are present and the mature spikes show different degrees of curvature, all inflorescences remain unbranched. No lateral spike-bearing branchlets, secondary rachises, or compound spike structures are observed.  The curved and variable orientation of the spikes is interpreted as a normal mature inflorescence feature and/or herbarium pressing effect rather than true rachis branching. The specimen shows no transitional morphology toward the branched spike architecture of *Elymus chaobotryus*. |
| PE00488728 | *Elymus dahuricus* Turcz. | Inner Mongolia Autonomous Region, China | PE | No | Very Low | None | The specimen represents a typical simple-spike form of *Elymus dahuricus*. The inflorescences consist of single elongated rachises with spikelets directly attached along the main axis.  Although multiple flowering culms are present and some spikes show a curved or spreading appearance, all visible inflorescences remain unbranched. No lateral spike-bearing branchlets, secondary rachises, or compound spike structures are observed.  The curved and drooping appearance of the mature spikes is interpreted as a normal inflorescence feature and/or herbarium pressing effect rather than true rachis branching. The specimen shows no transitional morphology toward the branched spike architecture of *Elymus chaobotryus*. |
| 15855003x0014 | *Elymus dahuricus* Turcz. | Inner Mongolia Autonomous Region, China | IFP | No | Very Low | None | The specimen represents a typical simple-spike form of *Elymus dahuricus*. The inflorescences consist of single elongated rachises with spikelets directly attached along the main axis.  No lateral spike-bearing branchlets, secondary rachises, or compound spike structures are observed. The specimen does not show transitional morphology toward the branched spike architecture of *Elymus chaobotryus*. |
| 15855003x0049 | *Elymus dahuricus* Turcz. | Inner Mongolia Autonomous Region, China | IFP | No | Very Low | None | The specimen represents a typical simple-spike form of *Elymus dahuricus*. The inflorescence consists of a single elongated rachis with spikelets directly attached along the main axis.  Although the spike is slightly curved, this appearance is interpreted as a normal mature spike posture and/or a consequence of herbarium pressing rather than true rachis branching.  No lateral spike-bearing branchlets, secondary rachises, or compound spike structures are observed. The specimen does not show transitional morphology toward the branched spike architecture of *Elymus chaobotryus*. |
| IFP0250207 | *Elymus dahuricus* Turcz. | Inner Mongolia Autonomous Region, China | IFP | No | Very Low | None | The specimen represents a typical simple-spike form of *Elymus dahuricus*. The inflorescences consist of single elongated rachises with spikelets directly attached along the main axis.  The spikes show a slightly curved and drooping appearance, which is interpreted as normal variation in mature inflorescence posture and/or a consequence of herbarium pressing rather than true rachis branching.  No lateral spike-bearing branchlets, secondary rachises, or compound spike structures are observed. The specimen does not show transitional morphology toward the branched spike architecture of *Elymus chaobotryus*. |
| IFP0257959 | *Elymus dahuricus* Turcz. | Inner Mongolia Autonomous Region, China | IFP | No | Very Low | None | The specimen represents a typical simple-spike form of *Elymus dahuricus*. The inflorescence consists of a single elongated rachis with spikelets directly attached along the main axis.  The spike is slender and slightly curved, which is interpreted as a normal mature inflorescence posture and/or an effect of herbarium pressing rather than true rachis branching.  No lateral spike-bearing branchlets, secondary rachises, or compound spike structures are observed. The specimen does not show transitional morphology toward the branched spike architecture of *Elymus chaobotryus*. |
| IFP0255721 | *Elymus dahuricus* Turcz. | Inner Mongolia Autonomous Region, China | IFP | No | Very Low | None | The specimen represents a typical simple-spike form of *Elymus dahuricus*. The inflorescences consist of single elongated rachises with spikelets directly attached along the main axis.  The spikes are relatively dense and slightly curved, but the apparent variation in spike shape is interpreted as normal mature inflorescence variation and/or a consequence of herbarium pressing rather than true rachis branching.  No lateral spike-bearing branchlets, secondary rachises, or compound spike structures are observed. The specimen does not show transitional morphology toward the branched spike architecture of *Elymus chaobotryus*. |
| N019106448 | *Elymus dahuricus* Turcz. | Inner Mongolia Autonomous Region, China | N | No | Very Low | None | The specimen represents a typical simple-spike form of *Elymus dahuricus*. The inflorescence consists of a single elongated rachis with spikelets directly attached along the main axis.  The spike shows a slender and slightly curved appearance, which is interpreted as normal variation in mature spike morphology and/or a result of herbarium pressing rather than true rachis branching.  No lateral spike-bearing branchlets, secondary rachises, or compound spike structures are observed. The specimen does not show transitional morphology toward the branched spike architecture of *Elymus chaobotryus*. |
| BJFC00004738 | *Elymus dahuricus* Turcz. ex Griseb. | Inner Mongolia Autonomous Region, China | BJFC | No | Very Low | None | The specimen represents a typical simple-spike form of *Elymus dahuricus*. The inflorescence consists of a single elongated rachis with spikelets directly attached along the main axis.  The spike is slender and slightly curved, which is interpreted as normal mature inflorescence variation and/or an effect of herbarium pressing rather than true rachis branching.  No lateral spike-bearing branchlets, secondary rachises, or compound spike structures are observed. The specimen does not show transitional morphology toward the branched spike architecture of *Elymus chaobotryus*. |
| PE01856937 | *Elymus dahuricus* Turcz. | Inner Mongolia Autonomous Region, China | PE | No | Very Low | None | The specimen represents a typical simple-spike phenotype of *Elymus dahuricus* Turcz. The inflorescences are composed of a single rachis bearing directly attached spikelets.  No lateral spike-bearing branchlets, secondary rachises, or compound spike structures are observed. The slightly curved and drooping appearance of the spikes represents normal variation associated with inflorescence development and herbarium preservation.  This specimen provides no evidence of transitional morphology toward the branched spike architecture characteristic of *Elymus chaobotryus*. It should be classified as a **non-branched spike form** of *Elymus dahuricus*. |
| PE00488730 | *Elymus dahuricus* Turcz. | Inner Mongolia Autonomous Region, China | PE | No | Very Low | None | The specimen represents a typical unbranched spike form of *Elymus dahuricus* Turcz. The inflorescence consists of a single elongated rachis bearing directly attached spikelets. No lateral spike-bearing branchlets or secondary inflorescence axes are observed.  The curved and elongated appearance of the spike is considered normal variation caused by inflorescence development and herbarium pressing. The specimen does not exhibit any morphological transition toward the compound branched spike characteristic of *Elymus chaobotryus*. |
| IFP15855003x0004 | *Elymus dahuricus* Turcz. | Inner Mongolia Autonomous Region, China | IFP | No | Very Low | None | This specimen represents a typical unbranched spike form of *Elymus dahuricus* Turcz.  The inflorescence consists of a single elongated rachis with spikelets directly attached to the main axis. No lateral spike-bearing branchlets, secondary rachises, or compound spike structures are observed.  The slender and slightly curved spike morphology represents normal variation associated with spike elongation and herbarium preservation. There is no evidence of transitional development toward the branched spike morphology characteristic of *Elymus chaobotryus*. |
| N 019106447 | *Elymus dahuricus* Turcz. | Inner Mongolia Autonomous Region, China | N | No | Very Low | None | This specimen represents a typical simple spike phenotype of *Elymus dahuricus* Turcz.  The inflorescence consists of elongated solitary spikes with spikelets directly attached to the main rachis. No lateral spike-bearing branchlets or secondary rachises are observed.  Although the spikes are elongated and strongly curved due to drying and herbarium pressing, the fundamental inflorescence architecture remains unbranched. The specimen shows no transitional characters toward the compound branched spike morphology found in *Elymus chaobotryus*. |
| FGC0002353 | *Elymus dahuricus* Turcz. | Inner Mongolia Autonomous Region, China | FGC | No | Very Low | None | This specimen represents a typical unbranched spike form of *Elymus dahuricus* Turcz.  The inflorescence consists of a single elongated rachis bearing spikelets directly attached to the main axis. No lateral spike-bearing branchlets or secondary rachises are observed.  The dense and elongated appearance of the spike represents normal variation within *E. dahuricus*, influenced by spike development and herbarium preservation. The specimen does not show any transitional morphology toward the compound branched spike characteristic of *Elymus chaobotryus*. |
| IATM0003970 | *Elymus dahuricus* Turcz. ex Griseb. | Inner Mongolia Autonomous Region, China | IATM | No | Very Low | None | This specimen represents a typical simple spike phenotype of *Elymus dahuricus* Turcz. ex Griseb.  The inflorescence consists of a single elongated rachis bearing directly attached spikelets. No lateral spike-bearing branchlets or secondary inflorescence axes are observed.  The strongly curved and drooping appearance of the spike represents normal morphological variation associated with rachis flexibility, spike maturation, and herbarium preservation. It does not represent a branched spike structure.  The specimen shows no transitional characters toward the compound branched spike morphology characteristic of *Elymus chaobotryus*. |
| PE00488818 | *Elymus dahuricus* Turcz. | Inner Mongolia Autonomous Region, China | PE | No | Very Low | None | This specimen represents a typical simple spike phenotype of *Elymus dahuricus* Turcz.  The inflorescence consists of a single elongated rachis with spikelets directly attached along the axis. No lateral spike-bearing branchlets or secondary inflorescence axes are observed.  The specimen contains multiple culms, but each inflorescence unit remains unbranched. The elongated and slightly drooping spikes represent normal variation caused by spike maturation and herbarium preservation.  No characters associated with the branched spike morphology of *Elymus chaobotryus* are present. |
| PE00488729 | *Elymus dahuricus* Turcz. | Inner Mongolia Autonomous Region, China | PE | No | Very Low | None | This specimen represents a typical simple spike phenotype of *Elymus dahuricus* Turcz.  The inflorescences consist of single elongated rachises with spikelets directly attached along the main axis. No lateral spike-bearing branches or secondary rachis axes are visible.  Although several spikes occur on the sheet, they represent separate tillers rather than a branched inflorescence system.  The diagnostic compound spike architecture of *Elymus chaobotryus*, including lateral spike-bearing branchlets arising from rachis nodes, is absent. |
| IFP 15855003X0061 | *Elymus dahuricus* Turcz. | Inner Mongolia Autonomous Region, China | IFP | No | Very Low | None | This specimen represents a typical simple spike phenotype of *Elymus dahuricus* Turcz.  The inflorescence consists of a single elongated rachis with spikelets directly attached along the main axis. No lateral spike-bearing branches or secondary rachis axes are observed.  The apparent multiple spike structures are derived from different reproductive culms rather than branching within a single inflorescence.  No diagnostic characters associated with the compound spike morphology of *Elymus chaobotryus* are present. |
| N019106445 | *Elymus dahuricus* Turcz. | Inner Mongolia Autonomous Region, China | N | No | Very Low | None | This specimen represents a typical simple spike phenotype of *Elymus dahuricus* Turcz.  The inflorescence consists of a single elongated rachis with spikelets directly attached along the main axis. No lateral spike-bearing branches or secondary inflorescence axes are observed.  The preserved spike is slightly curved and drooping, which is consistent with normal variation in mature *E. dahuricus* spikes.  No diagnostic characters associated with the compound spike morphology of *Elymus chaobotryus* are present. |
| N019106446 | *Elymus dahuricus* Turcz. ex Griseb. | Inner Mongolia Autonomous Region, China | N | No | Very Low | None | This specimen represents a typical simple spike phenotype of *Elymus dahuricus* Turcz. ex Griseb.  The inflorescence consists of a single elongated rachis with spikelets directly attached along the main axis. No lateral spike-bearing branches or secondary inflorescence axes are observed.  Although several spike-like structures are present on the herbarium sheet, they represent different reproductive culms rather than branching within a single inflorescence.  The diagnostic compound spike characters of *Elymus chaobotryus*, including lateral spike-bearing branchlets arising from rachis nodes, are absent. |
| PE01027486 | *Elymus dahuricus* Turcz. | Sichuan,China | PE | No | Very Low | None | This specimen represents a typical simple spike phenotype of *Elymus dahuricus* Turcz.  The inflorescence consists of a single elongated rachis with spikelets directly attached along the main axis. No lateral spike-bearing branches or secondary inflorescence axes are observed.  Although multiple spike-like structures are present on the herbarium sheet, they represent separate reproductive culms rather than branching within a single inflorescence.  The diagnostic compound spike characters of *Elymus chaobotryus*, including lateral spike-bearing branchlets arising from rachis nodes, are absent. |
| PE 00489062 | *Elymus dahuricus* Turcz. | Sichuan,China | PE | No | Very Low | None | This specimen represents a typical simple spike phenotype of *Elymus dahuricus* Turcz.  The inflorescence consists of a single elongated rachis with spikelets directly attached along the main axis. No lateral spike-bearing branches or secondary inflorescence axes are observed.  Although multiple spike-like structures occur on the herbarium sheet, they represent separate reproductive culms rather than branching within a single inflorescence.  The diagnostic compound spike characters of *Elymus chaobotryus*, including lateral spike-bearing branchlets arising from rachis nodes, are absent. |
| KUN337215 | *Elymus dahuricus* Turcz. | Sichuan,China | KUN | No | Very Low | None | This specimen represents a typical simple spike phenotype of *Elymus dahuricus* Turcz.  The inflorescence consists of a single elongated rachis with spikelets directly attached along the main axis. No lateral spike-bearing branches or secondary inflorescence axes are observed.  Although several vegetative and reproductive structures are present on the herbarium sheet, no branching occurs within a single inflorescence.  The diagnostic compound spike characters of *Elymus chaobotryus*, including lateral spike-bearing branchlets arising from rachis nodes, are absent. |
| KUN 337216 | *Elymus dahuricus* Turcz. | Sichuan,China | KUN | No | Very Low | None | This specimen represents a slender and flexuous simple spike phenotype of *Elymus dahuricus* Turcz., collected from a mountain slope margin in Sichuan Province at 2440 m elevation.  The inflorescence is characterized by a narrow, elongated spike with a flexible rachis. Spikelets are continuously arranged along a single main axis and directly attached to the rachis, forming a typical simple spike architecture. The mature spike shows a distinctly curved to slightly drooping orientation, representing a morphological variation within the simple spike type.  No lateral spike-bearing branches or secondary inflorescence axes are developed from rachis nodes. Therefore, the compound spike architecture diagnostic of *Elymus chaobotryus*, including branchlets carrying additional spikelets, is completely absent.  Although multiple reproductive culms occur on the herbarium sheet, each culm bears an independent simple spike rather than a branched inflorescence. |
| SZ 02071516 | *Elymus dahuricus* Turcz. | Sichuan,China | SZ | No | Very Low | None | This specimen represents an elongated and pendulous simple spike phenotype of *Elymus dahuricus* Turcz., collected from a roadside habitat in Sichuan Province at 1347 m elevation.  The inflorescence is characterized by a very slender and elongated rachis, forming a flexible spike that curves downward at maturity. Spikelets are arranged continuously along a single main axis and directly attached to the rachis, resulting in a typical simple spike architecture.  Although the spike shows a pronounced drooping habit and a lax, elongated appearance, the rachis remains completely unbranched. No lateral spike-bearing branchlets or secondary inflorescence axes arise from rachis nodes.  Compared with the compound spike structure of *Elymus chaobotryus*, this specimen lacks the essential branching architecture, including rachis node-derived lateral branches bearing additional spikelets. The pendulous spike form observed here represents intraspecific variation within the simple spike phenotype of *E. dahuricus* rather than an intermediate form toward compound spikes. |
| PE00489130 | *Elymus dahuricus* Turcz. | Sichuan,China | PE | No | Very Low | None | This specimen represents an elongated curved simple spike phenotype of *Elymus dahuricus* Turcz.  The inflorescence is characterized by a long and flexible rachis forming a distinctly curved, pendulous spike. Spikelets are arranged continuously along a single main axis and directly attached to the rachis, producing a typical simple spike architecture.  Although the mature spike shows a pronounced arching and drooping appearance, the rachis remains completely uninterrupted and lacks any lateral spike-bearing branchlets. No secondary inflorescence axes or rachis node-derived branches are observed.  The curved spike habit represents morphological variation within the simple spike phenotype of *E. dahuricus* rather than a transition toward compound spike formation. In contrast, the diagnostic characters of *Elymus chaobotryus*, including lateral branches bearing additional spikelets, are completely absent. |
| PE 00489085 | *Elymus dahuricus* Turcz. | Sichuan,China | PE | No | Very Low | None | This specimen represents a robust and relatively dense simple spike phenotype of *Elymus dahuricus* Turcz.  The inflorescence is characterized by a sturdy, elongated rachis forming an upright spike. Spikelets are densely and continuously arranged along a single central axis, with each spikelet directly attached to the main rachis. The spike maintains a compact cylindrical appearance compared with the more lax and flexuous forms observed in some other populations.  No lateral spike-bearing branchlets or secondary inflorescence axes are developed from rachis nodes. Although multiple reproductive culms are present on the herbarium sheet, each culm bears an independent simple spike rather than a branched inflorescence.  The compact and robust spike morphology represents an intraspecific variation of the simple spike phenotype in *E. dahuricus*. The essential compound spike characters of *Elymus chaobotryus*, including rachis node-derived lateral branches carrying additional spikelets, are completely absent. |
| PE 00489072 | *Elymus dahuricus* Turcz. | Sichuan,China | PE | No | Very Low | None | This specimen represents an elongated and flexuous simple spike phenotype of *Elymus dahuricus* Turcz.  The inflorescence is characterized by a slender, elongated rachis forming a curved and flexible spike. Spikelets are continuously arranged along a single main axis and directly attached to the rachis, maintaining the typical simple spike architecture.  The mature spike displays a pronounced flexuous curvature and a relatively lax appearance, reflecting variation in rachis flexibility and spike posture within *E. dahuricus*. However, the rachis remains completely unbranched, with no lateral spike-bearing branchlets or secondary inflorescence axes arising from rachis nodes.  Although several reproductive culms are present on the herbarium sheet, each culm produces an independent simple spike rather than a compound inflorescence. The diagnostic characters of *Elymus chaobotryus*, including rachis node-derived lateral branches bearing additional spikelets, are absent. |
| PE 00489066 | *Elymus dahuricus* Turcz. | Sichuan,China | PE | No | Very Low | None | This specimen represents an erect and compact simple spike phenotype of *Elymus dahuricus* Turcz., collected from a shrub habitat at 3200 m elevation in Sichuan Province.  The inflorescence forms a relatively robust and upright spike with a continuous central rachis. Spikelets are regularly arranged along the main axis and directly attached to the rachis, producing a typical simple spike architecture.  Compared with the more flexuous and pendulous spike forms found in some populations of *E. dahuricus*, this specimen shows a straighter rachis and a denser spike structure. However, the inflorescence remains completely unbranched, with no lateral spike-bearing branchlets or secondary axes arising from rachis nodes.  The compound spike characters of *Elymus chaobotryus*, including rachis node-derived branches carrying additional spikelets, are absent. The upright and compact appearance represents morphological variation within the simple spike phenotype rather than an intermediate condition toward compound spikes. |
| N 019106506 | *Elymus dahuricus* var. *coleotrichus* Keng | Sichuan,China | N | No | Very Low | None | This specimen represents a slender and lax simple spike phenotype of *Elymus dahuricus* var. *coleotrichus* Keng, collected from open grassland in Sichuan Province.  The inflorescence is characterized by a narrow and flexible rachis forming an elongated, open spike. Spikelets are loosely arranged along a single continuous main axis and directly attached to the rachis, maintaining a typical simple spike architecture.  The spike shows a delicate and extended appearance, with prominent awns and a slightly flexuous rachis. However, despite its elongated and lax morphology, the inflorescence axis remains completely unbranched. No lateral spike-bearing branchlets or secondary inflorescence axes arise from rachis nodes.  The observed variation in spike laxity and rachis flexibility represents morphological diversity within the simple spike phenotype of *Elymus dahuricus*. The diagnostic compound spike characters of *Elymus chaobotryus*, including rachis node-derived lateral branches carrying additional spikelets, are absent. |
| WCSBG 014869 | *Elymus dahuricus* Turcz. | Sichuan,China | WCSBG | No | Very Low | None | This specimen represents a slender and open simple spike phenotype of *Elymus dahuricus* Turcz., collected from Sichuan Province at 1900 m elevation.  The inflorescence is characterized by a narrow and elongated spike with a flexible rachis. Spikelets are loosely arranged along a single continuous main axis and directly attached to the rachis, forming a typical simple spike architecture.  The spike exhibits a relatively open appearance with extended awns and a slightly flexuous rachis, representing a lightweight and lax form within the morphological variation of *E. dahuricus*. Despite its elongated and loosely arranged spike, the inflorescence axis remains completely unbranched.  No lateral spike-bearing branchlets or secondary inflorescence axes arise from rachis nodes. The diagnostic compound spike structure of *Elymus chaobotryus*, including rachis node-derived branches carrying additional spikelets, is absent. |
| PE 01027485 | *Elymus dahuricus* Turcz. | Sichuan,China | PE | No | Very Low | None | This specimen represents an elongated lax pendulous simple spike phenotype of *Elymus dahuricus* Turcz., collected from a gravelly riverbank habitat in Sichuan Province at 2900 m elevation.  The inflorescence is characterized by a slender and flexible rachis forming a long, curved, and distinctly pendulous spike. Spikelets are loosely arranged along a single continuous main axis and directly attached to the rachis, maintaining the typical simple spike architecture.  The mature spike shows a pronounced drooping posture and an elongated appearance, representing a highly flexible spike form within the morphological variation of *E. dahuricus*. Despite its strongly curved and pendulous habit, the rachis remains completely unbranched.  No lateral spike-bearing branchlets or secondary inflorescence axes arise from rachis nodes. The diagnostic compound spike features of *Elymus chaobotryus*, including rachis node-derived lateral branches carrying additional spikelets, are absent. |
| PE 01027482 | *Elymus dahuricus* Turcz. | Sichuan,China | PE | No | Very Low | None | This specimen represents a slender and flexuous simple spike phenotype of *Elymus dahuricus* Turcz., collected from a gravelly riverbank habitat in Sichuan Province at 2900 m elevation.  The inflorescence is characterized by a slender rachis forming a curved and flexible spike. Spikelets are arranged along a single continuous main axis and directly attached to the rachis, maintaining the typical simple spike architecture.  The spike shows a relatively delicate appearance with a flexuous rachis and moderately lax spikelet arrangement. This morphology represents a flexible and elongated form within the natural variation of *E. dahuricus*. However, the inflorescence axis remains completely unbranched.  No lateral spike-bearing branchlets or secondary inflorescence axes arise from rachis nodes. The diagnostic compound spike characters of *Elymus chaobotryus*, including rachis node-derived branches carrying additional spikelets, are absent. |
| PE 00489074 | *Elymus dahuricus* Turcz. | Sichuan,China | PE | No | Very Low | None | This specimen represents an elongated lax flexuous simple spike phenotype of *Elymus dahuricus* Turcz., collected from a roadside habitat in Sichuan Province at 3500 m elevation.  The inflorescence is characterized by a slender and flexible rachis forming a long, curved spike. Spikelets are loosely arranged along a single continuous main axis and directly attached to the rachis, maintaining the typical simple spike architecture.  The spike shows an open and delicate appearance with a flexuous rachis and slightly pendulous posture. This morphology represents a flexible spike form within the natural variation of *E. dahuricus*, especially under high-elevation environments. However, the inflorescence remains completely unbranched.  No lateral spike-bearing branchlets or secondary inflorescence axes are developed from rachis nodes. The diagnostic compound spike characters of *Elymus chaobotryus*, including rachis node-derived lateral branches carrying additional spikelets, are absent. |
| PE 00489073 | *Elymus dahuricus* Turcz. | Sichuan,China | PE | No | Very Low | None | This specimen represents a robust and compact simple spike phenotype of *Elymus dahuricus* Turcz., collected from a roadside habitat in Sichuan Province at 3500 m elevation.  The inflorescence is characterized by a relatively stout rachis forming a compact and well-developed spike. Spikelets are densely arranged along a single continuous main axis and directly attached to the rachis, representing a typical simple spike architecture.  Compared with the more elongated and flexuous spike forms found in other populations of *E. dahuricus*, this specimen shows a shorter, denser, and more robust spike structure. The rachis remains continuous and unbranched, with no lateral spike-bearing branchlets or secondary inflorescence axes.  The compact appearance reflects morphological variation within the simple spike phenotype rather than an intermediate condition toward compound spikes. The diagnostic characters of *Elymus chaobotryus*, including rachis node-derived lateral branches carrying additional spikelets, are absent. |
| SZ02027779 | *Elymus dahuricus* Turcz. | Sichuan,China | SZ | No | Very Low | None | This specimen represents a slender lax alpine simple spike phenotype of *Elymus dahuricus* Turcz., collected from Sichuan Province at an extremely high elevation of 4663 m.  The inflorescence is characterized by a delicate and elongated rachis forming a narrow spike. Spikelets are loosely arranged along a single continuous main axis and directly attached to the rachis, representing the typical simple spike architecture.  The specimen shows a compact alpine growth form, with several reproductive culms arising from a densely tufted base. The spike exhibits a lightweight appearance, slightly flexuous rachis, and relatively open spikelet arrangement, which may reflect adaptation to high-elevation environments.  Despite its slender and flexible appearance, the inflorescence axis remains completely unbranched. No lateral spike-bearing branchlets or secondary inflorescence axes arise from rachis nodes.  The diagnostic compound spike characters of *Elymus chaobotryus*, including rachis node-derived lateral branches carrying additional spikelets, are absent. |
| SAUT 201400819 | *Elymus nutans* Griseb. | Qinghai, China | SAUT | No | Very Low | None | This specimen represents a typical pendulous simple spike phenotype of *Elymus nutans* Griseb., collected from Qinghai Province at an elevation of 4300 m.  The inflorescence consists of a slender and flexible rachis with spikelets directly attached along the main axis. The spike is elongated, relatively lax, and strongly curved downward, showing the characteristic pendulous architecture commonly observed in *E. nutans*.  The specimen exhibits a high-altitude growth form with tufted perennial habit and multiple reproductive culms. The inflorescences are soft and flexible, with elongated awns and loosely arranged spikelets, forming a typical alpine pendulous spike.  Although the spike axis is elongated and curved, the rachis remains completely unbranched. No lateral spike-bearing branches, secondary axes, or rachis node-derived branchlets are observed.  The diagnostic compound spike features of *Elymus chaobotryus*, including lateral branches arising from rachis nodes and carrying additional spikelets, are absent. |
| 201400818 | *Elymus nutans* Griseb. | Qinghai, China | SAUT | No | Very Low | None | This specimen represents an elongated pendulous simple spike phenotype of *Elymus nutans* Griseb., collected from Qinghai Province at an elevation of 4300 m.  The inflorescence is characterized by a slender and flexible rachis forming a long, curved, and strongly drooping spike. Spikelets are arranged along a single continuous rachis and directly attached to the main axis, representing a typical simple spike architecture.  Compared with more compact spike forms within related *Elymus* taxa, this specimen shows a particularly elongated and flexible inflorescence, with a pronounced pendulous orientation and relatively loose spikelet arrangement. Such morphology represents a characteristic alpine form of *E. nutans* rather than a transitional condition toward compound spikes.  The rachis remains completely unbranched throughout the inflorescence. No lateral spike-bearing branches, secondary axes, or rachis node-derived branchlets are observed.  The diagnostic compound spike characters of *Elymus chaobotryus*, including lateral spike-bearing branches arising from rachis nodes and carrying additional spikelets, are absent. |
| 201400648 | *Elymus nutans* Griseb. | Qinghai, China | SAUT | No | Very Low | None | This specimen represents a flexuous pendulous simple spike phenotype of *Elymus nutans* Griseb., collected from Qinghai Province at an elevation of 3050 m.  The inflorescence is characterized by an elongated and flexible rachis forming a curved, arching spike. Spikelets are directly attached along a single continuous main axis and arranged in a relatively lax manner, representing the typical simple spike architecture of *E. nutans*.  Compared with extremely high-altitude populations showing strongly reduced and compact growth forms, this specimen exhibits a more elongated and expanded inflorescence, with a flexible rachis and pronounced curvature. The spike architecture reflects normal morphological variation within *E. nutans* populations rather than structural modification toward a compound spike.  Although the inflorescence appears complex due to its curved and overlapping appearance on the herbarium sheet, the rachis remains completely unbranched. No lateral spike-bearing branches, secondary axes, or rachis node-derived branchlets are observed.  The diagnostic compound spike features of *Elymus chaobotryus*, including lateral spike-bearing branches arising from rachis nodes and carrying additional spikelets, are absent. |
| 201400643 | *Elymus nutans* Griseb. | Qinghai, China | SAUT | No | Very Low | None | This specimen represents a multi-culm elongated pendulous simple spike phenotype of *Elymus nutans* Griseb., collected from Qinghai Province at an elevation of 3250 m.  The specimen is characterized by numerous reproductive culms arising from a perennial tufted base, with each culm bearing an independent elongated spike. Individual inflorescences consist of a single flexible rachis, with spikelets directly attached along the main axis.  The spikes are slender, curved, and strongly pendulous, showing the typical soft and flexible inflorescence architecture of *E. nutans*. Compared with specimens having fewer but longer individual spikes, this material exhibits a multi-spike growth form with several drooping inflorescences developed simultaneously.  Although multiple spikes are present on the herbarium sheet, they represent separate reproductive culms rather than branching within a single inflorescence. The rachis of each spike remains continuous and unbranched.  No lateral spike-bearing branches, secondary inflorescence axes, or rachis node-derived branchlets are observed.  The diagnostic compound spike characters of *Elymus chaobotryus*, including lateral branches arising from rachis nodes and carrying additional spikelets, are absent. |
| 201400640 | *Elymus nutans* Griseb. | Qinghai, China | SAUT | No | Very Low | None | This specimen represents a robust simple spike phenotype of *Elymus nutans* Griseb., collected from Qinghai Province at an elevation of 3150 m.  The specimen is characterized by a tufted perennial growth form with multiple reproductive culms arising from the basal crown. Each reproductive culm terminates in an independent elongated spike. The inflorescence consists of a single continuous rachis with spikelets directly attached along the main axis.  The spikes are relatively dense and cylindrical, showing a more compact spike architecture compared with lax and strongly pendulous forms of *E. nutans*. The rachis is straight to slightly curved, and conspicuous awns extend beyond the spikelets, producing the typical awned appearance of the species.  Although several spikes are present on the herbarium sheet, each spike represents an independent simple inflorescence produced by separate reproductive culms. No branching occurs within an individual spike.  The diagnostic compound spike characters of *Elymus chaobotryus*, including lateral spike-bearing branchlets arising from rachis nodes and forming secondary spike structures, are absent. |
| 201400635 | *Elymus nutans* Griseb. | Qinghai, China | SAUT | No | Very Low | None | This specimen represents a typical lax and pendulous simple spike phenotype of *Elymus nutans* Griseb., collected from Qinghai Province at an elevation of 3200 m.  The plant forms a perennial tussock with multiple reproductive culms arising from the basal crown. Each culm terminates in an independent elongated spike. The inflorescence axis is slender and flexible, resulting in distinctly curved to pendulous spikes. Spikelets are arranged directly along the continuous rachis, producing a typical simple spike architecture.  Compared with more compact and erect forms of *E. nutans*, this specimen is characterized by relatively lax spike arrangement and strongly arched rachises. The elongated flexible spikes and pronounced drooping habit represent a distinctive morphological expression within the species.  Although several spikes are present on the herbarium sheet, they correspond to separate reproductive culms rather than branching within a single inflorescence. No lateral spike-bearing branches or secondary rachis structures are observed.  The diagnostic compound spike features of *Elymus chaobotryus*, including rachis node-derived lateral spike-bearing branchlets and a compound spike system, are completely absent. |
| 201400627 | *Elymus nutans* Griseb. | Qinghai, China | SAUT | No | Very Low | None | This specimen represents a curved and pendulous simple spike phenotype of *Elymus nutans* Griseb., collected from Qinghai Province at an elevation of 3700 m.  The specimen shows a perennial tussock habit with reproductive culms arising from the basal crown. Each reproductive culm produces a single elongated spike. The inflorescence consists of a continuous rachis with spikelets directly attached along the main axis.  A distinctive feature of this specimen is the strongly curved and flexible spike rachis. The mature spikes exhibit an arching posture, with the distal portion becoming pendulous. Compared with more compact and erect forms of *E. nutans*, this material shows a relatively lax spike arrangement and a more pronounced drooping architecture.  Although several spike structures occur on the herbarium sheet, each represents an independent simple inflorescence produced by separate reproductive culms. No lateral spike-bearing branchlets or secondary inflorescence axes are present within any spike.  The diagnostic compound spike features of *Elymus chaobotryus*, including rachis node-derived lateral spike branches and a branched spike architecture, are absent. |
| 201400589 | *Elymus nutans* Griseb. | Qinghai, China | SAUT | No | Very Low | None | This specimen represents a curved compact simple spike phenotype of *Elymus nutans* Griseb., collected from Qinghai Province.  The specimen shows a perennial tussock growth form with reproductive culms arising from the basal crown. Each culm terminates in a single elongated spike. The inflorescence architecture is composed of a continuous rachis with spikelets directly attached along the main axis.  The spikes are relatively compact and moderately dense, with a distinctly curved rachis and a slightly pendulous distal portion. Compared with lax and strongly drooping forms of *E. nutans*, this specimen exhibits a more condensed spike structure while maintaining the typical flexible rachis characteristic of the species.  Several reproductive culms are present on the herbarium sheet, but each inflorescence remains an independent simple spike. No lateral spike-bearing branches, secondary rachis axes, or internal branching structures are observed.  The diagnostic compound spike characters of *Elymus chaobotryus*, including rachis node-derived lateral spike branches and a branched inflorescence system, are absent. |
| 201400586 | *Elymus nutans* Griseb. | Sichuan, China | SAUT | No | Very Low | None | This specimen represents a typical lax and pendulous simple spike phenotype of *Elymus nutans* Griseb. from Sichuan Province.  The plant shows a perennial tussock growth form with multiple reproductive culms arising from the basal crown. Each reproductive culm terminates in a single spike composed of a continuous rachis bearing spikelets directly along the main axis.  The spike is slender, flexible, and strongly curved, showing the typical pendulous inflorescence architecture of *E. nutans*. Spikelets are arranged continuously along the rachis without any secondary axes or branching structures.  No rachis node branching, lateral spike-bearing branches, secondary spike axes, or compound rachis system are observed. Therefore, this specimen represents a typical simple spike morphology and does not show any transitional characters toward compound spike forms such as *Elymus chaobotryus*. |
| 201400904 | *Elymus sibiricus* L. | Sichuan, China | SAUT | No | Very Low | None | This specimen represents a typical simple spike phenotype of *Elymus sibiricus* L.  The inflorescence consists of a single continuous rachis with spikelets directly attached along the main axis. No lateral spike-bearing branches, secondary spike axes, or compound rachis systems are observed.  Although multiple reproductive culms are present on the herbarium sheet, each inflorescence remains independent and unbranched. The observed variation in spike length, curvature, and density represents normal morphological variation within *Elymus sibiricus*.  The diagnostic compound spike characters of *Elymus chaobotryus*, including rachis node branching and lateral spike-bearing branchlets, are absent. |
| 201400580 | *Elymus nutans* (Keng) Keng | Qinghai, China | SAUT | No | Very Low | None | This specimen represents a typical simple spike phenotype of *Elymus nutans* (Keng) Keng.  The inflorescence consists of a single elongated and flexible rachis with spikelets directly attached along the main axis. The spike is slender and strongly curved, showing the characteristic pendulous spike architecture of *E. nutans*.  No rachis node branching, lateral spike-bearing branches, secondary spike axes, or compound rachis systems are observed. Although the spike shows considerable curvature and lax arrangement, these features represent normal variation within the simple spike morphology of *E. nutans*.  The diagnostic compound spike characters of *Elymus chaobotryus*, including lateral spike-bearing branchlets arising from rachis nodes, are absent. |
| 01905015 | *Elymus nutans* Griseb. | Qinghai, China | PE | No | Very Low | None | This specimen represents a typical simple spike phenotype of *Elymus nutans* Griseb.  The inflorescence consists of a single elongated and flexible rachis with spikelets directly attached along the main axis. The spike is strongly curved and pendulous, showing the characteristic inflorescence architecture of *E. nutans*.  No rachis node branching, lateral spike-bearing branches, secondary spike axes, or compound rachis systems are observed.  Although the spike shows a curved and lax appearance, these characteristics represent normal variation within the simple spike morphology of *E. nutans*.  The diagnostic compound spike characters of *Elymus chaobotryus*, including lateral spike-bearing branchlets arising from rachis nodes, are absent. |
| 01904685 | *Elymus nutans* Griseb. | Qinghai, China | PE | No | Very Low | None | This specimen represents a typical simple spike phenotype of *Elymus nutans* Griseb.  The inflorescence consists of a single elongated rachis with spikelets directly attached along the main axis. The spike axis is slender and curved, showing the characteristic pendulous spike architecture of *E. nutans*.  No rachis node branching, lateral spike-bearing branches, secondary spike axes, or compound rachis systems are observed.  Although the spike is curved and relatively lax, these features represent normal morphological variation within *Elymus nutans*.  The diagnostic compound spike characters of *Elymus chaobotryus*, including lateral spike-bearing branches arising from rachis nodes, are absent. |
| 01904519 | *Elymus nutans* Griseb. | Qinghai, China | PE | No | Very Low | None | The inflorescence consists of a single elongated rachis with spikelets directly attached along the main axis. The spike axis is flexible and strongly curved, showing the characteristic pendulous spike architecture of *Elymus nutans*.  No rachis node branching, lateral spike-bearing branches, secondary spike axes, or compound rachis systems are observed.  Although the spike is long, curved, and lax, these characteristics represent normal morphological variation within *Elymus nutans*.  The diagnostic compound spike characters of *Elymus chaobotryus*, including lateral spike-bearing branchlets arising from rachis nodes, are absent. |
| 00489241 | *Elymus nutans* Griseb. | Qinghai, China | PE | No | Very Low | None | This specimen represents a typical simple spike phenotype of *Elymus nutans* Griseb.  The inflorescence consists of a single elongated rachis with spikelets directly attached along the main axis. The spike is slender and curved, showing the characteristic pendulous spike architecture of *Elymus nutans*.  No rachis node branching, lateral spike-bearing branches, secondary spike axes, or compound rachis systems are observed.  Although the inflorescence is flexible and curved, these features represent normal variation within *Elymus nutans*.  The diagnostic compound spike characters of *Elymus chaobotryus*, including lateral spike-bearing branches arising from rachis nodes, are absent. |
| 00489239 | *Elymus nutans* Griseb. | Qinghai, China | PE | No | Very Low | None | This specimen represents a typical simple spike phenotype of *Elymus nutans* Griseb.  The inflorescence consists of a single elongated rachis with spikelets directly attached along the main axis. The spike is slender, curved, and pendulous, showing the characteristic spike architecture of *Elymus nutans*.  No rachis node branching, lateral spike-bearing branches, secondary spike axes, or compound rachis systems are observed.  Although the spike axis is curved and the inflorescence appears lax, these features represent normal variation within *Elymus nutans*.  The diagnostic compound spike characters of *Elymus chaobotryus*, including lateral spike-bearing branches arising from rachis nodes, are absent. |
| 00489237 | *Elymus nutans* Griseb. | Qinghai, China | PE | No | Very Low | None | This specimen represents a typical simple spike phenotype of *Elymus nutans* Griseb.  The inflorescence consists of a single elongated rachis with spikelets directly attached along the main axis. The spike is slender, slightly curved, and pendulous, representing the typical spike architecture of *Elymus nutans*.  No rachis node branching, lateral spike-bearing branches, secondary spike axes, or compound rachis systems are observed.  Although the inflorescence is elongated and slightly lax, these features represent normal variation within *Elymus nutans*.  The diagnostic compound spike characters of *Elymus chaobotryus*, including lateral spike-bearing branches arising from rachis nodes, are absent. |
| 00489234 | *Elymus nutans* Griseb. | Qinghai, China | PE | No | Very Low | None | This specimen represents a typical simple spike phenotype of *Elymus nutans* Griseb.  The inflorescence consists of a single elongated rachis with spikelets directly attached along the main axis. The spike is slender, curved, and slightly pendulous, representing the typical spike architecture of *Elymus nutans*.  No rachis node branching, lateral spike-bearing branches, secondary spike axes, or compound rachis systems are observed.  Although the inflorescence shows a relatively lax arrangement and curved rachis, these features represent normal variation within *Elymus nutans*.  The diagnostic compound spike characters of *Elymus chaobotryus*, including lateral spike-bearing branches arising from rachis nodes, are absent. |
| 00489232 | *Elymus nutans* Griseb. | Qinghai, China | PE | No | Very Low | None | This specimen represents a typical simple spike phenotype of *Elymus nutans* Griseb.  The inflorescence consists of a single elongated rachis with spikelets directly attached along the main axis. The spike is slender, curved, and pendulous, representing the typical spike architecture of *Elymus nutans*.  No rachis node branching, lateral spike-bearing branches, secondary spike axes, or compound rachis systems are observed.  Although the spike is relatively elongated and strongly curved, these features represent normal morphological variation within *Elymus nutans*.  The diagnostic compound spike characters of *Elymus chaobotryus*, including lateral spike-bearing branches arising from rachis nodes, are absent. |
| 00489231 | *Elymus nutans* Griseb. | Qinghai, China | PE | No | Very Low | None | This specimen represents a typical simple spike phenotype of *Elymus nutans* Griseb.  The inflorescence consists of a single elongated rachis with spikelets directly attached along the main axis. The spike is slender, curved, and pendulous, representing the typical spike architecture of *Elymus nutans*.  No rachis node branching, lateral spike-bearing branches, secondary spike axes, or compound rachis systems are observed.  Although the inflorescence is elongated and curved, these features represent normal morphological variation within *Elymus nutans*.  The diagnostic compound spike characters of *Elymus chaobotryus*, including lateral spike-bearing branches arising from rachis nodes, are absent. |
| 00489225 | *Elymus nutans* Griseb. | Qinghai, China | PE | No | Very Low | None | This specimen represents a typical simple spike phenotype of *Elymus nutans* Griseb.  The inflorescence consists of a single elongated rachis with spikelets directly attached along the main axis. The spike is slender, slightly curved, and lacks any branching structure.  No rachis node branching, lateral spike-bearing branches, secondary spike axes, or compound rachis systems are observed.  The elongated spike and slightly pendulous orientation represent normal morphological variation within *Elymus nutans*.  The diagnostic compound spike characters of *Elymus chaobotryus*, including lateral spike-bearing branchlets arising from rachis nodes, are absent. |
| 00489222 | *Elymus nutans* Griseb. | Qinghai, China | PE | No | Very Low | None | This specimen represents a typical simple spike phenotype of *Elymus nutans* Griseb.  The inflorescence consists of a single elongated rachis with spikelets directly attached along the main axis. The spike is slender and strongly curved or pendulous, which is consistent with the typical morphology of *Elymus nutans*.  No rachis node branching, lateral spike-bearing branches, secondary spike axes, or compound rachis systems are observed.  Although the spike shows a pronounced drooping orientation, this represents normal variation within *Elymus nutans* rather than a branched spike phenotype.  The diagnostic compound spike characters of *Elymus chaobotryus*, including lateral spike-bearing branchlets arising from rachis nodes, are absent. |
| 00489220 | *Elymus nutans* Griseb. | Qinghai, China | PE | No | Very Low | None | This specimen represents a typical simple spike phenotype of *Elymus nutans* Griseb.  The inflorescence consists of a single elongated rachis with spikelets directly attached along the main axis. The spike is slender and slightly pendulous, consistent with the typical morphology of *Elymus nutans*.  No rachis node branching, lateral spike-bearing branches, secondary spike axes, or compound rachis systems are observed.  The drooping orientation of the spike represents normal variation within *Elymus nutans* and does not indicate a branched spike structure.  The diagnostic compound spike characters of *Elymus chaobotryus*, including lateral spike-bearing branchlets arising from rachis nodes, are absent. |
| 00489218 | *Elymus nutans* Griseb. | Qinghai, China | PE | No | Very Low | None | This specimen represents a typical simple spike phenotype of *Elymus nutans* Griseb.  The inflorescence consists of a single elongated rachis with spikelets directly attached along the main axis. The spike is slender and curved, showing the typical pendulous tendency of *Elymus nutans*.  No rachis node branching, lateral spike-bearing branches, secondary spike axes, or compound rachis systems are observed.  The curved spike architecture represents normal morphological variation within *Elymus nutans* and does not indicate a branched spike phenotype.  The diagnostic compound spike characters of *Elymus chaobotryus*, including lateral spike-bearing branchlets arising from rachis nodes, are absent. |
| 00489213 | *Elymus nutans* Griseb. | Qinghai, China | PE | No | Very Low | None | This specimen represents a typical simple spike phenotype of *Elymus nutans* Griseb.  The inflorescence consists of a single elongated rachis with spikelets directly attached along the main axis. The spike is slender and curved, showing the typical pendulous morphology of *Elymus nutans*.  No rachis node branching, lateral spike-bearing branches, secondary spike axes, or compound rachis systems are observed.  Although the inflorescence shows a curved and drooping appearance, this represents normal variation within *Elymus nutans* rather than a branched spike phenotype.  The diagnostic compound spike characters of *Elymus chaobotryus*, including lateral spike-bearing branchlets arising from rachis nodes, are absent. |
| 00489202 | *Elymus nutans* Griseb. | Qinghai, China | PE | No | Very Low | None | This specimen represents a typical simple spike phenotype of *Elymus nutans* Griseb.  The inflorescence consists of a single elongated rachis with spikelets directly attached along the main axis. The spike is strongly curved and pendulous, representing the typical inflorescence architecture of *Elymus nutans*.  No rachis node branching, lateral spike-bearing branches, secondary spike axes, or compound rachis systems are observed.  The curved and drooping appearance of the spike represents normal variation within *Elymus nutans* and is not associated with a branched spike structure.  The diagnostic compound spike characters of *Elymus chaobotryus*, including lateral spike-bearing branches arising from rachis nodes, are absent. |
| 019107072 | *Elymus nutans* Griseb. | Qinghai, China | N | No | Very Low | None | This specimen represents a typical simple spike phenotype of *Elymus nutans* Griseb.  The inflorescence consists of a single elongated rachis with spikelets directly attached along the main axis. The spike is slender and curved, showing the typical pendulous morphology of *Elymus nutans*.  No rachis node branching, lateral spike-bearing branches, secondary spike axes, or compound rachis systems are observed.  The curved spike appearance represents normal morphological variation within *Elymus nutans* and does not indicate a transitional form toward compound spike architecture.  The diagnostic compound spike characters of *Elymus chaobotryus*, including lateral spike-bearing branchlets arising from rachis nodes, are absent. |
| 019107070 | *Elymus nutans* Griseb. | Qinghai, China | N | No | Very Low | None | This specimen represents a typical simple spike phenotype of *Elymus nutans* Griseb.  The inflorescence consists of a single elongated rachis with spikelets directly attached along the main axis. The spike is slender and curved, showing the typical pendulous morphology of *Elymus nutans*.  No rachis node branching, lateral spike-bearing branches, secondary spike axes, or compound rachis systems are observed.  The curved spike architecture represents normal variation within *Elymus nutans* and does not indicate a transitional stage toward compound spike morphology.  The diagnostic compound spike characters of *Elymus chaobotryus*, including lateral spike-bearing branchlets arising from rachis nodes, are absent. |
| 019107069 | *Elymus nutans* Griseb. | Qinghai, China | N | No | Very Low | None | This specimen represents a typical simple spike phenotype of *Elymus nutans* Griseb.  The specimen shows multiple reproductive culms arising from a perennial tufted base. Each inflorescence consists of a single elongated rachis with spikelets directly attached along the main axis.  The spikes are slender, curved, and pendulous, which represents normal morphological variation of *Elymus nutans*.  No rachis node branching, lateral spike-bearing branches, secondary spike axes, or compound spike structures are observed.  Therefore, this specimen does not represent a transitional form toward *Elymus chaobotryus*. |
| 019107019 | *Elymus nutans* Griseb. | Qinghai, China | N | No | Very Low | None | This specimen represents a typical simple spike phenotype of *Elymus nutans* Griseb.  The plant forms a perennial tufted habit with multiple reproductive culms. Each inflorescence consists of a single elongated rachis with spikelets directly attached along the main axis.  The spikes are curved and pendulous, showing the characteristic morphology of *Elymus nutans*.  No rachis node branching, lateral spike-bearing branches, secondary inflorescence axes, or compound spike structures are observed.  Although multiple spikes occur on the specimen sheet, each spike represents an independent simple inflorescence rather than a branched compound spike. |
| 019107007 | *Elymus nutans* Griseb. | Qinghai, China | N | No | Very Low | None | This specimen represents a typical simple spike phenotype of *Elymus nutans* Griseb.  The specimen shows a perennial tufted grass habit with reproductive culms arising from a compact basal crown. The inflorescence consists of a single elongated rachis with spikelets directly attached along the main axis.  The spike is slender, curved, and pendulous, representing normal variation of *Elymus nutans*.  No rachis node branching, lateral spike-bearing branches, secondary inflorescence axes, or compound spike structures are observed.  The specimen therefore does not show any transitional morphology toward the branched spike condition of *Elymus chaobotryus*. |
| 019107002 | *Elymus nutans* Griseb. | Qinghai, China | N | No | Very Low | None | This specimen represents a typical simple spike phenotype of *Elymus nutans* Griseb.  The specimen shows a perennial tufted grass habit with slender reproductive culms. The inflorescence consists of a single elongated rachis with spikelets directly attached along the main axis.  The spikes are curved and pendulous, representing the typical morphology of *Elymus nutans*.  No rachis node branching, lateral spike-bearing branches, secondary inflorescence axes, or compound spike structures are observed.  The specimen does not show any transitional characters toward the branched spike morphology of *Elymus chaobotryus*. |
| 337302 | *Elymus nutans* Griseb. | Qinghai, China | KUN | No | Very Low | None | This specimen represents a typical simple spike phenotype of *Elymus nutans* Griseb.  The specimen shows a perennial tufted grass habit with slender reproductive culms. The inflorescence consists of a single elongated rachis with spikelets directly attached along the main axis.  The spike is curved and slightly pendulous, representing the typical morphology of *Elymus nutans*.  No rachis node branching, lateral spike-bearing branches, secondary inflorescence axes, or compound spike structures are observed.  The specimen does not show transitional characters toward the branched spike morphology of *Elymus chaobotryus*. |
| 337301 | *Elymus nutans* Griseb. | Qinghai, China | KUN | No | Very Low | None | This specimen represents a typical simple spike phenotype of *Elymus nutans* Griseb.  The specimen shows a perennial tufted grass habit with slender reproductive culms. The inflorescence consists of a single elongated rachis with spikelets directly attached along the main axis.  The spike is strongly curved and pendulous, representing the typical morphology of *Elymus nutans*.  No rachis node branching, lateral spike-bearing branches, secondary inflorescence axes, or compound spike structures are observed.  The specimen lacks any transitional characters toward the branched spike morphology of *Elymus chaobotryus*. |
| 337300 | *Elymus nutans* Griseb. | Qinghai, China | KUN | No | Very Low | None | This specimen represents a typical simple spike phenotype of *Elymus nutans* Griseb.  The specimen shows a perennial tufted grass habit with several slender reproductive culms. The inflorescence consists of a single rachis with spikelets directly attached along the main axis.  The spike is slightly curved with compact spikelet arrangement, representing the normal variation of *Elymus nutans*.  No rachis node branching, lateral spike-bearing branches, secondary inflorescence axes, or compound spike structures are observed.  The specimen lacks any transitional characters toward the branched spike morphology of *Elymus chaobotryus*. |
| 337299 | *Elymus nutans* Griseb. | Qinghai, China | KUN | No | Very Low | None | This specimen represents a typical simple spike phenotype of *Elymus nutans* Griseb.  The specimen shows a perennial tufted grass habit with slender reproductive culms. The inflorescence consists of a single elongated rachis with spikelets directly attached along the main axis.  The spike is elongated and slightly curved, representing the typical morphology of *Elymus nutans*.  No rachis node branching, lateral spike-bearing branches, secondary inflorescence axes, or compound spike structures are observed.  The specimen lacks any transitional characters toward the branched spike morphology of *Elymus chaobotryus*. |
| 1566440 | *Elymus nutans* Griseb. | Qinghai, China | KUN | No | Very Low | None | This specimen represents a typical simple spike phenotype of *Elymus nutans* Griseb.  The specimen shows a perennial tufted grass habit with several slender reproductive culms. The inflorescence consists of a single elongated rachis with spikelets directly attached along the main axis.  The spike is curved and pendulous, representing the typical morphology of *Elymus nutans* from alpine meadow habitats.  No rachis node branching, lateral spike-bearing branches, secondary inflorescence axes, or compound spike structures are observed.  The specimen does not show any transitional characters toward the branched spike morphology of *Elymus chaobotryus*. |
| 1459217 | *Elymus nutans* Griseb. | Qinghai, China | KUN | No | Very Low | None | This specimen represents a typical simple spike phenotype of *Elymus nutans* Griseb.  The specimen shows a perennial tufted grass habit with slender reproductive culms. The inflorescence consists of a single elongated rachis with spikelets directly attached along the main axis.  The spike is elongated, slightly curved and pendulous, representing the typical morphology of *Elymus nutans*.  No rachis node branching, lateral spike-bearing branches, secondary inflorescence axes, or compound spike structures are observed.  The specimen lacks any transitional characters toward the branched spike morphology of *Elymus chaobotryus*. |
| 1401640 | *Elymus nutans* Griseb. | Qinghai, China | KUN | No | Very Low | None | This specimen represents a typical simple spike phenotype of *Elymus nutans* Griseb.  The specimen shows a perennial tufted grass habit with several erect reproductive culms. The inflorescence consists of a single elongated rachis with spikelets directly attached along the main axis.  The spike is elongated, strongly curved and pendulous, representing the typical morphology of *Elymus nutans* from alpine meadow habitats.  No rachis node branching, lateral spike-bearing branches, secondary inflorescence axes, or compound spike structures are observed.  The specimen lacks any transitional characters toward the branched spike morphology of *Elymus chaobotryus*. |
| 1401595 | *Elymus nutans* Griseb. | Qinghai, China | KUN | No | Very Low | None | This specimen represents a typical simple spike phenotype of *Elymus nutans* Griseb.  The specimen shows a perennial tufted grass habit with several slender reproductive culms. The inflorescence consists of a single elongated rachis with spikelets directly attached along the main axis.  The spike is elongated, curved and pendulous, representing the typical morphology of *Elymus nutans* from Qinghai valley habitats.  No rachis node branching, lateral spike-bearing branches, secondary inflorescence axes, or compound spike structures are observed.  The specimen lacks any transitional characters toward the branched spike morphology of *Elymus chaobotryus*. |
| HNWP00019136 | *Elymus nutans* Griseb. | Qinghai, China | HNWP | No | Very Low | None | This specimen represents a typical simple spike phenotype of *Elymus nutans* Griseb.  The specimen shows a perennial tufted grass habit with slender reproductive culms. The inflorescence consists of a single elongated rachis with spikelets directly attached along the main axis.  The spike is elongated, curved and pendulous, representing the typical morphology of *Elymus nutans* from high-altitude Qinghai habitats.  No rachis node branching, lateral spike-bearing branches, secondary inflorescence axes, or compound spike structures are observed.  The specimen lacks any transitional characters toward the branched spike morphology of *Elymus chaobotryus*. |
| 99374 | *Elymus nutans* Griseb. | Qinghai, China | HNWP | No | Very Low | None | This specimen represents a typical simple spike phenotype of *Elymus nutans* Griseb.  The specimen shows a perennial tufted grass habit with multiple reproductive culms. The inflorescence consists of a single rachis with spikelets directly attached along the main axis.  The spike is compact and relatively erect, representing a high-altitude ecological form of *Elymus nutans*.  No rachis node branching, lateral spike-bearing branches, secondary inflorescence axes, or compound spike structures are observed.  The specimen lacks any transitional characters toward the branched spike morphology of *Elymus chaobotryus*. |
| 9482 | *Elymus nutans* Griseb. | Qinghai, China | HNWP | No | Very Low | None | This specimen represents a typical simple spike phenotype of *Elymus nutans* Griseb.  The specimen shows a perennial tufted grass habit with multiple reproductive culms. The inflorescences consist of single elongated rachises with spikelets directly attached along the main axes.  The spikes are strongly curved and pendulous, representing the typical high-altitude morphology of *Elymus nutans*.  No rachis node branching, lateral spike-bearing branches, secondary inflorescence axes, or compound spike structures are observed.  The specimen lacks any transitional characters toward the branched spike morphology of *Elymus chaobotryus*. |
| 74085 | *Elymus nutans* Griseb. | Qinghai, China | HNWP | No | Very Low | None | This specimen represents a typical simple spike phenotype of *Elymus nutans* Griseb.  The specimen shows a perennial tufted grass habit with multiple reproductive culms. The inflorescences consist of single elongated rachises with spikelets directly attached along the main axes.  The spikes are slender and slightly curved, representing the normal variation of *Elymus nutans*.  No rachis node branching, lateral spike-bearing branches, secondary inflorescence axes, or compound spike structures are observed.  The specimen lacks any transitional characters toward the branched spike morphology of *Elymus chaobotryus*. |
| 36388 | *Elymus nutans* Griseb. | Qinghai, China | HNWP | No | Very Low | None | This specimen represents a typical simple spike phenotype of *Elymus nutans* Griseb.  The specimen shows a perennial tufted grass habit with multiple reproductive culms. The inflorescences consist of single elongated rachises with spikelets directly attached along the main axes.  The spikes are slender, curved and slightly pendulous, representing the typical morphology of *Elymus nutans*.  No rachis node branching, lateral spike-bearing branches, secondary inflorescence axes, or compound spike structures are observed.  The specimen lacks any transitional characters toward the branched spike morphology of *Elymus chaobotryus*. |
| 198599 | *Elymus nutans* Griseb. | Qinghai, China | HNWP | No | Very Low | None | This specimen represents a typical simple spike phenotype of *Elymus nutans* Griseb.  The specimen shows a perennial tufted grass habit with several reproductive culms. The inflorescences consist of single rachises with spikelets directly attached along the main axes.  The spikes are slender and slightly curved, representing the normal variation of *Elymus nutans*.  No rachis node branching, lateral spike-bearing branches, secondary inflorescence axes, or compound spike structures are observed.  The specimen lacks any transitional characters toward the branched spike morphology of *Elymus chaobotryus*. |
| 198528 | *Elymus nutans* Griseb. | Qinghai, China | HNWP | No | Very Low | None | This specimen represents a typical simple spike phenotype of *Elymus nutans* Griseb.  The specimen shows a perennial tufted grass habit with multiple reproductive culms. The inflorescences consist of single rachises with spikelets directly attached along the main axes.  The spikes are slender, curved and slightly pendulous, representing the typical morphology of *Elymus nutans*.  No rachis node branching, lateral spike-bearing branches, secondary inflorescence axes, or compound spike structures are observed.  The specimen lacks any transitional characters toward the branched spike morphology of *Elymus chaobotryus*. |
| 197234 | *Elymus nutans* Griseb. | Qinghai, China | HNWP | No | Very Low | None | This specimen represents a typical simple spike phenotype of *Elymus nutans* Griseb.  The specimen shows a perennial tufted grass habit with multiple reproductive culms. The inflorescences consist of single rachises with spikelets directly attached along the main axes.  The spikes are relatively long and compact, representing the typical simple spike morphology of *Elymus nutans*.  No rachis node branching, lateral spike-bearing branches, secondary inflorescence axes, or compound spike structures are observed.  The specimen lacks any transitional characters toward the branched spike morphology of *Elymus chaobotryus*. |
| 186072 | *Elymus nutans* Griseb. | Qinghai, China | HNWP | No | Very Low | None | This specimen represents a typical simple spike phenotype of *Elymus nutans* Griseb.  The specimen shows a perennial tufted grass habit with several reproductive culms. The inflorescences consist of single elongated rachises with spikelets directly attached along the main axes.  The spikes are slender, curved and strongly pendulous, representing the typical morphology of *Elymus nutans*.  No rachis node branching, lateral spike-bearing branches, secondary inflorescence axes, or compound spike structures are observed.  The specimen lacks any transitional characters toward the branched spike morphology of *Elymus chaobotryus*. |
| 18178 | *Elymus nutans* Griseb. | Qinghai, China | HNWP | No | Very Low | None | This specimen represents a typical simple spike phenotype of *Elymus nutans* Griseb.  The specimen shows a perennial tufted grass habit collected from Qinghai at high elevation. The inflorescences consist of single elongated rachises with spikelets directly attached along the main axis.  The spikes are slender, curved and partially pendulous, representing the typical morphology of *Elymus nutans*.  No rachis node branching, lateral spike-bearing branches, secondary inflorescence axes, or compound spike structures are observed.  The specimen lacks any transitional characters toward the branched spike morphology of *Elymus chaobotryus*. |
| 178858 | *Elymus nutans* Griseb. | Qinghai, China | HNWP | No | Very Low | None | This specimen represents a typical simple spike phenotype of *Elymus nutans* Griseb.  The specimen shows a perennial tufted grass habit collected from Qinghai at an elevation of approximately 4500 m. The inflorescences consist of single elongated rachises with spikelets directly attached along the main axis.  The spikes are slender, curved and strongly pendulous, representing the typical morphology of *Elymus nutans*.  No rachis node branching, lateral spike-bearing branches, secondary inflorescence axes, or compound spike structures are observed.  The specimen lacks any transitional characters toward the branched spike morphology of *Elymus chaobotryus*. |
| 00000913 | *Elymus nutans* Griseb. | Qinghai, China | HNWP | No | Very Low | None | This specimen represents a typical simple spike phenotype of *Elymus nutans* Griseb.  The specimen shows a perennial tufted grass habit collected from Qinghai at an elevation of approximately 3850–3950 m. The inflorescences consist of single elongated rachises with spikelets directly attached along the main axis.  The spikes are slender and slightly curved, representing the typical morphology of *Elymus nutans*.  No rachis node branching, lateral spike-bearing branches, secondary inflorescence axes, or compound spike structures are observed.  The specimen lacks any transitional characters toward the branched spike morphology of *Elymus chaobotryus*. |
| 0212811 | *Elymus nutans* Griseb. | Qinghai, China | HNWP | No | Very Low | None | This specimen represents a typical simple spike phenotype of *Elymus nutans* Griseb.  The specimen shows a perennial tufted grass habit with multiple reproductive culms. The inflorescences consist of single elongated rachises with spikelets directly attached along the main axis.  The spikes are slender, curved and pendulous, representing the typical morphology of *Elymus nutans*.  No rachis node branching, lateral spike-bearing branches, secondary inflorescence axes, or compound spike structures are observed.  The specimen lacks any transitional characters toward the branched spike morphology of *Elymus chaobotryus*. |
| 201401009 | *Elymus sibiricus* L. | Qinghai, China | SAUT | No | Very Low | None | This specimen represents a typical simple spike phenotype of *Elymus sibiricus* L.  The specimen shows a perennial tufted grass habit with erect culms. The inflorescence consists of a single elongated rachis with spikelets directly attached along the main axis.  No rachis branching, lateral spike-bearing branches, secondary inflorescence axes, or compound spike structures are observed.  Compared with branched-spike taxa such as *Elymus chaobotryus*, this specimen lacks any morphological evidence of spike axis branching. |
| 201400958 | *Elymus sibiricus* L. | Qinghai, China | SAUT | No | Very Low | None | This specimen represents a typical simple spike phenotype of *Elymus sibiricus* L.  The specimen exhibits a perennial tufted grass habit. The inflorescence consists of a single elongated rachis with spikelets directly attached along the main axis.  No rachis branching, lateral spike-bearing branches, or secondary inflorescence axes are observed.  The specimen lacks morphological features associated with branched-spike taxa such as *Elymus chaobotryus*. |
| 00489661 | *Elymus sibiricus* L. | Qinghai, China | PE | No | Very Low | None | This specimen represents a typical simple spike phenotype of *Elymus sibiricus* L.  The inflorescence consists of a single elongated rachis with spikelets directly attached along the main axis. No rachis branching, lateral spike-bearing branches, or secondary inflorescence axes are observed.  The specimen lacks the compound spike architecture characteristic of branched-spike taxa such as *Elymus chaobotryus*. |
| 00489646 | *Elymus sibiricus* L. | Qinghai, China | PE | No | Very Low | None | This specimen represents a typical simple spike phenotype of *Elymus sibiricus* L.  The inflorescence consists of a single elongated rachis with spikelets directly attached along the main axis. No rachis branching, lateral spike-bearing branches, or secondary inflorescence axes are observed.  The specimen lacks the compound spike architecture characteristic of branched-spike taxa such as *Elymus chaobotryus*. |
| 019107146 | *Elymus sibiricus* L. | Qinghai, China | N | No | Very Low | None | This specimen represents a typical simple spike phenotype of *Elymus sibiricus* L.  The specimen shows a perennial tufted grass habit with slender reproductive culms. The inflorescence consists of a single elongated rachis with spikelets directly attached along the main axis.  The spike is curved and relatively loose, representing the typical morphology of *Elymus sibiricus*.  No rachis node branching, lateral spike-bearing branches, secondary inflorescence axes, or compound spike structures are observed.  The specimen does not show transitional characters toward the branched spike morphology of *Elymus chaobotryus*. |
| 019107092 | *Elymus sibiricus* L. | Qinghai, China | N | No | Very Low | None | This specimen represents a typical simple spike phenotype of *Elymus sibiricus* L.  The specimen shows a perennial tufted grass habit with multiple reproductive culms. The inflorescences consist of single elongated rachises with spikelets directly attached along the main axis.  The spikes are relatively robust and compact, with erect to slightly curved orientation and prominent awns.  No rachis node branching, lateral spike-bearing branches, secondary inflorescence axes, or compound spike structures are observed.  The specimen does not show transitional characters toward the branched spike morphology of *Elymus chaobotryus*. |
| 337259 | *Elymus sibiricus* L. | Qinghai, China | KUN | No | Very Low | None | This specimen represents a typical simple spike phenotype of *Elymus sibiricus* L.  The specimen shows a perennial tufted grass habit with numerous reproductive culms. The inflorescence consists of a single elongated rachis with spikelets directly attached along the main axis.  The spikes are curved and weakly pendulous, with relatively loose spikelet arrangement and prominent awns.  No rachis node branching, lateral spike-bearing branches, secondary inflorescence axes, or compound spike structures are observed.  The specimen does not show transitional characters toward the branched spike morphology of *Elymus chaobotryus*. |
| 1401678 | *Elymus sibiricus* L. | Qinghai, China | KUN | No | Very Low | None | This specimen represents a typical simple spike phenotype of *Elymus sibiricus* L.  The specimen was collected from an alpine grassland at 4715 m elevation. It shows a perennial tufted grass habit with well-developed basal tillers and slender reproductive culms.  The inflorescence consists of a single elongated rachis with spikelets directly attached along the main axis.  The spikes are erect to slightly curved, relatively loose, and possess prominent awns.  No rachis node branching, lateral spike-bearing branches, secondary inflorescence axes, or compound spike structures are observed.  The specimen does not show transitional characters toward the branched spike morphology of *Elymus chaobotryus*. |
| 98297 | *Elymus sibiricus* L. | Qinghai, China | HNWP | No | Very Low | None | This specimen represents a typical simple spike phenotype of *Elymus sibiricus* L.  The specimen shows a perennial tufted grass habit with slender reproductive culms. The inflorescence consists of a single elongated rachis with spikelets directly attached along the main axis.  The spikes are slightly curved with relatively loose spikelet arrangement and prominent awns.  No rachis node branching, lateral spike-bearing branches, secondary inflorescence axes, or compound spike structures are observed.  The specimen does not show transitional characters toward the branched spike morphology of *Elymus chaobotryus*. |
| 98230 | *Elymus sibiricus* L. | Qinghai, China | HNWP | No | Very Low | None | This specimen represents a typical simple spike phenotype of *Elymus sibiricus* L.  The specimen shows a perennial tufted grass habit with multiple reproductive culms. The inflorescence consists of a single elongated rachis with spikelets directly attached along the main axis.  The spikes are strongly curved and pendulous, showing the typical inflorescence architecture of *Elymus sibiricus*.  No rachis node branching, lateral spike-bearing branches, secondary inflorescence axes, or compound spike structures are observed.  The specimen does not show transitional characters toward the branched spike morphology of *Elymus chaobotryus*. |
| 98198 | *Elymus sibiricus* L. | Qinghai, China | HNWP | No | Very Low | None | This specimen represents a typical simple spike phenotype of *Elymus sibiricus* L.  The specimen shows a perennial tufted grass habit with multiple reproductive culms. The inflorescence consists of a single elongated rachis with spikelets directly attached along the main axis.  The spikes are elongated, curved and pendulous, representing the typical inflorescence morphology of *Elymus sibiricus*.  No rachis node branching, lateral spike-bearing branches, secondary inflorescence axes, or compound spike structures are observed.  The specimen does not show transitional characters toward the branched spike morphology of *Elymus chaobotryus*. |
| 98203 | *Elymus sibiricus* L. | Qinghai, China | HNWP | No | Very Low | None | This specimen represents a typical simple spike phenotype of *Elymus sibiricus* L.  The specimen shows a perennial tufted grass habit with numerous reproductive culms. The inflorescence consists of a single elongated rachis with spikelets directly attached along the main axis.  The spikes are elongated, curved and slightly pendulous, showing the typical simple spike architecture of *Elymus sibiricus*.  No rachis node branching, lateral spike-bearing branches, secondary inflorescence axes, or compound spike structures are observed.  The specimen does not show transitional characters toward the branched spike morphology of *Elymus chaobotryus*. |
| 96203 | *Elymus sibiricus* L. | Qinghai, China | HNWP | No | Very Low | None | This specimen represents a typical simple spike phenotype of *Elymus sibiricus* L.  The specimen shows a perennial tufted grass habit with multiple reproductive culms. The inflorescences consist of elongated single rachises with spikelets directly attached along the main axis.  The spikes are slender, curved and slightly pendulous, representing the typical simple spike morphology of *Elymus sibiricus*.  No rachis node branching, lateral spike-bearing branches, secondary inflorescence axes, or compound spike structures are observed.  The specimen does not show transitional characters toward the branched spike morphology of *Elymus chaobotryus*. |
| 73594 | *Elymus sibiricus* L. | Qinghai, China | HNWP | No | Very Low | None | This specimen represents a typical simple spike phenotype of *Elymus sibiricus* L.  The specimen shows a perennial tufted grass habit with slender reproductive culms. The inflorescence consists of a single elongated rachis with spikelets directly attached along the main axis.  The spikes are curved and pendulous, representing the typical simple spike morphology of *Elymus sibiricus*.  No rachis node branching, lateral spike-bearing branches, secondary inflorescence axes, or compound spike structures are observed.  The specimen does not show transitional characters toward the branched spike morphology of *Elymus chaobotryus*. |
| 73453 | *Elymus sibiricus* L. | Qinghai, China | HNWP | No | Very Low | None | This specimen represents a typical simple spike phenotype of *Elymus sibiricus* L.  The specimen shows a perennial tufted grass habit with slender reproductive culms. The inflorescence consists of a single elongated rachis with spikelets directly attached along the main axis.  The spikes are curved and slightly pendulous, representing the typical simple spike morphology of *Elymus sibiricus*.  No rachis node branching, lateral spike-bearing branches, secondary inflorescence axes, or compound spike structures are observed.  The specimen does not show transitional characters toward the branched spike morphology of *Elymus chaobotryus*. |
| 46960 | *Elymus sibiricus* L. | Qinghai, China | HNWP | No | Very Low | None | This specimen represents a typical simple spike phenotype of *Elymus sibiricus* L.  The specimen shows a perennial tufted grass habit with numerous reproductive culms. The inflorescences consist of single elongated rachises with spikelets directly attached along the main axis.  The spikes are slightly curved with well-developed awns, representing the typical morphology of *Elymus sibiricus*.  No rachis node branching, lateral spike-bearing branches, secondary inflorescence axes, or compound spike structures are observed.  The specimen does not show transitional characters toward the branched spike morphology of *Elymus chaobotryus*. |
| 154785 | *Elymus sibiricus* L. | Qinghai, China | HNWP | No | Very Low | None | This specimen represents a typical simple spike phenotype of *Elymus sibiricus* L.  The specimen shows a perennial tufted grass habit collected from a high-altitude region of Qinghai Province. The inflorescences consist of single elongated rachises with spikelets directly attached along the main axis.  The spikes are curved with well-developed awns, representing the typical morphology of *Elymus sibiricus*.  No rachis node branching, lateral spike-bearing branches, secondary inflorescence axes, or compound spike structures are observed.  The specimen does not show transitional characters toward the branched spike morphology of *Elymus chaobotryus*. |
| 0231983 | *Elymus sibiricus* L. | Qinghai, China | HNWP | No | Very Low | None | This specimen represents a typical simple spike phenotype of *Elymus sibiricus* L.  The specimen shows a perennial tufted grass habit with slender reproductive culms. The inflorescence consists of a single elongated rachis with spikelets directly attached along the main axis.  The spike is curved and elongated, showing the typical morphology of *Elymus sibiricus*.  No rachis node branching, lateral spike-bearing branches, secondary inflorescence axes, or compound spike structures are observed.  The specimen does not show any transitional characters toward the branched spike morphology of *Elymus chaobotryus*. |
| 02023037 | *Elymus trachycaulus* (Link) Gould ex Shinners | United States of America | PE | No | Very Low | None | This specimen represents a typical simple spike phenotype of *Elymus trachycaulus* (Link) Gould ex Shinners.  The specimen shows a perennial grass habit with slender reproductive culms. The inflorescence consists of a single elongated rachis with spikelets directly attached along the main axis.  The spike is narrow and relatively loose, representing the typical morphology of *Elymus trachycaulus*.  No rachis node branching, lateral spike-bearing branches, secondary inflorescence axes, or compound spike structures are observed.  The specimen does not show any transitional characters toward the branched spike morphology of *Elymus chaobotryus*. |
| 01779626 | *Elymus trachycaulus* (Link) Gould ex Shinners | United States of America | PE | No | Very Low | None | This specimen represents a typical simple spike phenotype of *Elymus trachycaulus* (Link) Gould ex Shinners.  The specimen shows a perennial tufted grass habit with multiple reproductive culms and well-developed basal tufts. The inflorescence consists of a single elongated rachis with spikelets directly attached along the main axis.  The spikes are narrow and relatively loose, representing the typical morphology of *Elymus trachycaulus*.  No rachis node branching, lateral spike-bearing branches, secondary inflorescence axes, or compound spike structures are observed.  The specimen does not show any transitional characters toward the branched spike morphology of *Elymus chaobotryus*. |
| 01779627 | *Elymus trachycaulus* (Link) Gould ex Shinners | United States of America | PE | No | Very Low | None | This specimen represents a typical simple spike phenotype of *Elymus trachycaulus* (Link) Gould ex Shinners.  The specimen shows a perennial tufted grass habit with well-developed roots and several reproductive culms. The inflorescence consists of a single elongated rachis with spikelets directly attached along the main axis.  The spike is narrow and elongated, showing the characteristic simple spike morphology of *Elymus trachycaulus*.  No rachis node branching, lateral spike-bearing branches, secondary inflorescence axes, or compound spike structures are observed.  The specimen does not show any transitional characters toward the branched spike morphology of *Elymus chaobotryus*. |
| NAS00561249 | *Elymus trachycaulus* (Link) Gould ex Shinners | United States of America | NAS | No | Very Low | None | This specimen represents a typical simple spike phenotype of *Elymus trachycaulus* (Link) Gould ex Shinners.  The specimen shows a perennial tufted grass habit with dense basal tillers and multiple reproductive culms. The inflorescence consists of a single elongated rachis with spikelets directly attached along the main axis.  The spikes are narrow and elongated, representing the typical simple spike morphology of *Elymus trachycaulus*.  No rachis node branching, lateral spike-bearing branches, secondary inflorescence axes, or compound spike structures are observed.  The specimen does not show any transitional characters toward the branched spike morphology of *Elymus chaobotryus*. |
| 003504 | *Elymus breviaristatus* (Keng) Keng f. | Qinghai, China | HNWP | No | Very Low | None | This specimen represents a typical simple spike phenotype of *Elymus breviaristatus* (Keng) Keng f.  The specimen shows a perennial tufted grass habit with basal clustering and slender reproductive culms. The inflorescence consists of a single curved rachis with spikelets directly attached along the main axis.  The spike is narrow and curved, with short awns and no evidence of rachis branching.  No lateral spike-bearing branches, secondary inflorescence axes, or compound spike structures are observed.  The specimen does not show any transitional characters toward the branched spike morphology of *Elymus chaobotryus*. |
| 162422 | *Elymus breviaristatus* (Keng) Keng f. | Qinghai, China | HNWP | No | Very Low | None | This specimen represents a typical simple spike phenotype of *Elymus breviaristatus* (Keng) Keng f.  The specimen shows a perennial tufted grass habit with a well-developed basal crown and several reproductive culms. The inflorescence consists of a single elongated curved rachis with spikelets directly attached along the main axis.  The spike is curved and narrow, with short awns, representing the typical simple spike morphology of *Elymus breviaristatus*.  No rachis node branching, lateral spike-bearing branches, secondary inflorescence axes, or compound spike structures are observed.  The specimen does not show any transitional characters toward the branched spike morphology of *Elymus chaobotryus*. |
| 57771 | *Elymus breviaristatus* (Keng) Keng f. | Qinghai, China | HNWP | No | Very Low | None | This specimen represents a typical simple spike phenotype of *Elymus breviaristatus* (Keng) Keng f.  The plant shows a perennial tufted habit with numerous reproductive culms arising from a dense basal crown. The inflorescence consists of a single elongated curved rachis, with spikelets directly attached along the main axis.  No rachis node branching, lateral spike-bearing branches, secondary inflorescence axes, or compound spike structures are observed.  The specimen represents a typical non-branched spike morphology and does not show transitional characters toward the branched spike phenotype found in *Elymus chaobotryus*. |
| 73957 | *Elymus breviaristatus* (Keng) Keng f. | Qinghai, China | HNWP | No | Very Low | None | This specimen represents a typical simple spike phenotype of *Elymus breviaristatus* (Keng) Keng f.  The plant shows a perennial tufted growth form with multiple culms arising from the basal crown. The inflorescence consists of elongated simple spikes with spikelets directly attached to the main rachis.  No rachis node branching, lateral spike-bearing branches, secondary inflorescence axes, or compound spike structures are observed.  The apparent separation of inflorescence portions in the pressed specimen is caused by multiple curved spikes rather than true branching of a single inflorescence axis.  This specimen represents a typical non-branched spike morphology. |
| 00012653 | *Elymus breviaristatus* (Keng) Keng f. | Qinghai, China | HNWP | No | Very Low | None | This specimen represents a typical simple spike phenotype of *Elymus breviaristatus* (Keng) Keng f.  The specimen shows a perennial tufted growth form collected from Qinghai at 3966 m elevation. The inflorescences consist of elongated simple spikes, with spikelets directly attached to the main rachis.  No rachis node branching, lateral spike-bearing branches, secondary inflorescence axes, or compound spike structures are observed.  The specimen represents a typical non-branched spike morphology of *E. breviaristatus* and does not show transitional characters toward the branched spike phenotype found in *Elymus chaobotryus*. |
| 201400426 | *Elymus breviaristatus* (Keng) Keng f. | Qinghai, China | SAUT | No | Very Low | None | This specimen represents a typical simple spike phenotype of *Elymus breviaristatus* (Keng) Keng f.  The specimen shows a perennial tufted growth form with multiple culms. The inflorescences are composed of elongated simple spikes, with spikelets directly attached to the main rachis.  No rachis node branching, lateral spike-bearing branches, secondary inflorescence axes, or compound spike structures are observed.  The specimen represents the typical non-branched spike morphology of *E. breviaristatus* and differs clearly from the branched spike architecture observed in *Elymus chaobotryus*. |
| 1401424 | *Elymus breviaristatus* Keng ex Keng f. | Qinghai, China | KUN | No | Very Low | None | This specimen represents a typical simple spike phenotype of *Elymus breviaristatus* Keng ex Keng f.  The specimen was collected from Qinghai Province at an elevation of 4190 m. It shows a typical alpine tufted perennial grass morphology with short stature and multiple culms.  The inflorescence consists of elongated simple spikes. Spikelets are directly attached to the main rachis, and no rachis branching, lateral spike-bearing branches, or secondary inflorescence axes are observed.  The specimen represents the non-branched spike morphology of *E. breviaristatus*, clearly differing from the compound branched spike architecture of *Elymus chaobotryus*. |
| 337182 | *Elymus atratus* (Nevski) Hand.-Mazz. | Qinghai, China | KUN | No | Very Low | None | his specimen represents a typical simple spike phenotype of *Elymus atratus* (Nevski) Hand.-Mazz.  The specimen was collected from Qinghai Province at an elevation of 4400 m near forest margins. It shows a typical alpine perennial grass habit with slender culms and tufted growth.  The inflorescence consists of a simple elongated spike. Spikelets are directly attached to the main rachis, and no rachis branching, lateral spike-bearing branches, or secondary inflorescence axes are observed.  This specimen represents the non-branched spike morphology of *E. atratus*, clearly differing from the compound branched spike architecture observed in *Elymus chaobotryus*. |
| 17374 | *Elymus atratus* (Nevski) Hand.-Mazz. | Qinghai, China | HNWP | No | Very Low | None | This specimen represents a typical simple spike phenotype of *Elymus atratus* (Nevski) Hand.-Mazz.  The specimen was collected from Qinghai Province at an elevation of 4200 m. It shows an alpine perennial grass habit with tufted growth and a compact reproductive structure.  The inflorescence is composed of a single spike axis. Spikelets are directly attached to the rachis, and no rachis branching, lateral spike-bearing branches, or secondary inflorescence axes are observed.  This specimen represents the non-branched spike morphology of *Elymus atratus*, clearly distinct from the compound branched spike morphology found in *Elymus chaobotryus*. |
| 97645 | *Elymus atratus* (Nevski) Hand.-Mazz. | Qinghai, China | HNWP | No | Very Low | None | This specimen represents a typical simple spike phenotype of *Elymus atratus* (Nevski) Hand.-Mazz.  The specimen was collected from Qinghai Province at an elevation of 4500 m. It shows an alpine perennial grass morphology with tufted growth and compact reproductive structures.  The inflorescence consists of a single rachis with spikelets directly attached to the main axis. No rachis branching, lateral spike-bearing branches, or secondary inflorescence axes are observed.  This specimen represents the non-branched spike phenotype of *Elymus atratus*, clearly distinct from the compound branched spike architecture of *Elymus chaobotryus*. |
| 00002921 | *Elymus atratus* (Nevski) Hand.-Mazz. | Qinghai, China | HNWP | No | Very Low | None | This specimen represents a typical simple spike phenotype of *Elymus atratus* (Nevski) Hand.-Mazz.  The specimen was collected from Qinghai Province at an elevation of 3584 m in a shrub slope habitat. It shows a perennial tufted grass morphology with narrow leaves and slender reproductive culms.  The inflorescence consists of a single rachis with spikelets directly attached to the main axis. No rachis branching, lateral spike-bearing branches, or secondary inflorescence axes are observed.  This specimen represents the non-branched spike phenotype of *Elymus atratus*, clearly distinct from the compound branched spike architecture of *Elymus chaobotryus*. |
| 201400413 | *Elymus atratus* (Nevski) Hand.-Mazz. | Qinghai, China | SAUT | No | Very Low | None | This specimen represents a typical simple spike phenotype of *Elymus atratus* (Nevski) Hand.-Mazz.  The specimen was collected from Qinghai Province at an elevation of 3250 m. It is a perennial tufted grass with elongated culms and narrow leaves.  The inflorescence consists of a single rachis with spikelets directly attached to the main axis. No rachis branching, lateral spike-bearing branches, or secondary inflorescence axes are observed.  This specimen represents the non-branched spike morphology of *Elymus atratus*, clearly distinct from the compound branched spike architecture found in *Elymus chaobotryus*. |
